# Demographic and traditional knowledge perspectives on the current status of Canadian polar bear subpopulations

**DOI:** 10.1002/ece3.2030

**Published:** 2016-03-23

**Authors:** Jordan York, Martha Dowsley, Adam Cornwell, Miroslaw Kuc, Mitchell Taylor

**Affiliations:** ^1^ Department of Geography and the EnvironmentLakehead University955 Oliver RoadThunder BayONP7B 5E1Canada; ^2^ Miroslaw KucPH 205‐942 Yonge StreetTorontoONM4W 3S8Canada

**Keywords:** Canada, climate change, co‐management, mark–recapture, polar bear, population viability analysis, RISKMAN, subpopulation status, traditional ecological knowledge, Traditional Ecological Knowledge, *Ursus maritimus*

## Abstract

Subpopulation growth rates and the probability of decline at current harvest levels were determined for 13 subpopulations of polar bears (*Ursus maritimus*) that are within or shared with Canada based on mark–recapture estimates of population numbers and vital rates, and harvest statistics using population viability analyses (PVA). Aboriginal traditional ecological knowledge (TEK) on subpopulation trend agreed with the seven stable/increasing results and one of the declining results, but disagreed with PVA status of five other declining subpopulations. The decline in the Baffin Bay subpopulation appeared to be due to over‐reporting of harvested numbers from outside Canada. The remaining four disputed subpopulations (Southern Beaufort Sea, Northern Beaufort Sea, Southern Hudson Bay, and Western Hudson Bay) were all incompletely mark–recapture (M‐R) sampled, which may have biased their survival and subpopulation estimates. Three of the four incompletely sampled subpopulations were PVA identified as nonviable (i.e., declining even with zero harvest mortality). TEK disagreement was nonrandom with respect to M‐R sampling protocols. Cluster analysis also grouped subpopulations with ambiguous demographic and harvest rate estimates separately from those with apparently reliable demographic estimates based on PVA probability of decline and unharvested subpopulation growth rate criteria. We suggest that the correspondence between TEK and scientific results can be used to improve the reliability of information on natural systems and thus improve resource management. Considering both TEK and scientific information, we suggest that the current status of Canadian polar bear subpopulations in 2013 was 12 stable/increasing and one declining (Kane Basin). We do not find support for the perspective that polar bears within or shared with Canada are currently in any sort of climate crisis. We suggest that monitoring the impacts of climate change (including sea ice decline) on polar bear subpopulations should be continued and enhanced and that adaptive management practices are warranted.

## Introduction

Our message is not simple or conventional or consistent with the dire warnings present in much of the polar bear literature since 2006 (Schliebe et al. 2006). We show that much of the scientific evidence indicating that some polar bear subpopulations are declining due to climate change‐mediated sea ice reductions is likely flawed by poor mark–recapture (M‐R) sampling and that the complex analysis models employed to overcome these capture issues apparently fail to provide accurate estimates of the demographic parameters used to determine subpopulation status. Our evidence is partly scientific (comparison to subsequent surveys), partly logical (the demographic estimates suggest a dramatic decline that has not occurred) and partly taken from Inuit and Inuvialuit traditional ecological knowledge (TEK). We do not attempt to describe why M‐R analysis appears to under‐estimate population numbers and survival rates when the sampling does not cover the entire subpopulation area, only to document that the logical projections that use demographic estimates from these analyses are not supported individually or collectively by subsequent surveys or TEK.

Our perspectives on climate warming and Arctic sea ice decline are developed from an empirical examination of the open‐source data on various indicators of these phenomena. We see reason for concern, but find no reliable evidence to support the contention that polar bears are currently experiencing a climate crisis. We suggest that the qualitative projections for dramatic reductions in population numbers and range are overly pessimistic given the response of polar bears, climate, and sea ice to the present. We qualify our demographic projections by considering the effects of increasing uncertainty that is inherent to stochastic projections and find that even projections based on sound estimates of vital rates eventually become too uncertain to provide accurate estimates of geometric mean population growth rate (*λ*). Our article considers published M‐R estimates of survival rates and population numbers, age structure estimates of recruitment, population viability analysis, aerial survey population estimates, and TEK for Canadian subpopulations. We also look empirically at reported Greenland harvest data and a climate‐related time series for sea ice, global temperature estimates, Arctic temperature estimates, and ocean temperature estimates. We choose to look at this information collectively rather than simply accept what others have written because we are concerned that the polarizing influence from climate politics may have generated perspectives about polar bear conservation that are more argumentative than objective. We felt it was necessary to adopt a system approach that included the components required for a comparative consideration of polar bear subpopulation status from demographic, environmental (climate), and TEK perspectives.

### Polar bears and the threat of climate warming

Polar bears have always been a symbol of the north and for many years were regarded as a conservation success story (Prestrud and Stirling 1994; Lunn et al. 2002). Recently, they have also become a poster species for “Second‐Wave” Environmentalists (Dearden and Mitchell 2009) seeking to convince policy makers and the public that anthropogenic global warming constitutes a climate crisis (Slocum 2004). Climate warming is predicted and observed to affect higher latitudes first and most (Intergovernmental Panel on Climate Change (IPCC), 2007, 2013), and Arctic sea ice during the open water season has been observed to be declining since satellite records began in 1978 (Parkinson et al. 1999; Comiso 2006; National Snow and Ice Data Center (NSIDC)). Sea ice is required for polar bear movements to feeding areas (Stirling and Derocher 1993; Ferguson et al. 2000, 2001; Amstrup 2003), to summer retreat areas onshore and on the multiyear pack ice (Stirling and Parkinson 2006; Durner et al. 2009), and to locate mates during breeding season (Ramsay and Stirling 1986; Stirling and Derocher 1993). Several studies have documented nutritional and recruitment impacts from sea ice reductions on polar bear subpopulations (Stirling et al. 1999, 2008; Obbard et al. 2006; Rode et al. 2007, 2010, 2014). Sea ice decline could negatively impact affected polar bear subpopulations.

Polar bears evolved from a common ancestor with the brown bear. The range of estimates for the age of polar bears as a species ranges from 4 million years based on deep nuclear genomic sequence data from both paternal and maternal linages (Miller et al. 2012) to 120 thousand years based on the mitochondrial genome (matrilineal) (Lindqvist et al. 2010). If polar bears have existed for the last 4 million years, they would have emerged during the mid‐Pliocene approximately 1.25 million years before the onset of northern hemisphere glacial cycles (Bartoli et al. 2005). If polar bears emerged any time prior to or during the previous glacial cycle, they would have persisted through the Eemian interglacial period. During the Eemian interglacial, mean annual temperatures were 4°C warmer than the current interglacial (Holocene) for northern latitudes (Müller 2009), and some northern locations reached temperatures as high as ~7.5°C warmer than the mean temperature for the same area over the last thousand years (Dahl‐Jensen et al. 2013). Both scenarios suggest that polar bears are able to mitigate impacts from sea ice decline to an extent not fully exhibited in modern times. Currently, the IPCC predicts globally averaged temperatures to warm ~2°C by 2100 and considers warming of ~4°C by 2100 to be possible although unlikely (Intergovernmental Panel on Climate Change 2013). Reduction in the heavy multiyear ice and increased productivity from a longer open water season may even enhance polar bear habitat in some areas (Stirling and Derocher 1993, 2012; Derocher et al. 2004; Rode et al. 2014). The majority of Canada's polar bears inhabit the Canadian Arctic archipelago (Obbard et al. 2010), where 5 of 13 subpopulations are currently and historically ice‐free in late summer and early fall (Lunn et al. 2002; Aars et al. 2006; Obbard et al. 2010). Given the persistence of polar bears through the current and previous interglacial periods, and their ability to accommodate extended retreats onshore and based on the empirical observations of climate and sea ice change (S7), it seems unlikely that polar bears (as a species) are at risk from anthropogenic global warming. However, some subpopulations may experience diminished range, reduced productivity and subsequent decline in numbers if sea ice declines occur as predicted (Stirling and Derocher 1993, 2012; Derocher et al. 2004). While there are many projections of climate change that suggest a nearly ice‐free Arctic to occur in the warmer months (i.e., September) (IPCC 2007, 2013, Durner et al. 2009; Amstrup et al. 2010; Mahlstein and Knutti 2012; Overland and Wang 2013), there are currently no global climate model (GCM) projections of climate change that suggest a totally ice‐free Arctic in any season or month.

**Figure 1:**
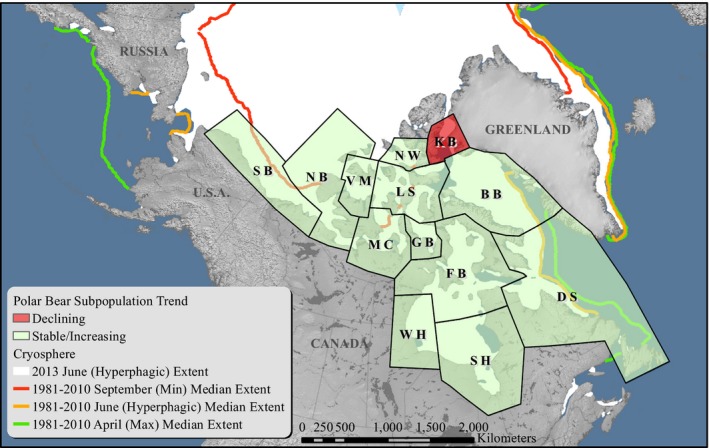
2013 Canadian polar bear subpopulation status, subpopulation boundaries, and minimum (September), maximum (April), and hyperphagic (June) sea ice extent. Boundaries of Canadian polar bear subpopulations are defined as: Canadian polar bear subpopulations are defined as: Baffin Bay (BB), Davis Strait (DS), Foxe Basin (FB), Gulf of Boothia (GB), Kane Basin (KB), Lancaster Sound (LS), M'Clintock Channel (MC), Northern Beaufort Sea (NB), Norwegian Bay (NW), Southern Beaufort Sea (SB), Southern Hudson Bay (SH), Viscount Melville Sound (VM), and Western Hudson Bay (WH). Data used for the production of this map were courtesy of NSIDC (http://nsidc.org/data/docs/noaa/g02135_seaice_index/) and Natural Earth (http://www.naturalearthdata.com/downloads/).

The nutritional and recruitment impacts from sea ice reductions on polar bear subpopulations are based on direct measures of individuals that would be less likely to be affected by partial (local) subsampling. However, polar bear subpopulation status estimates are derived mainly from M‐R estimates of subpopulation numbers and survival rates that are presumed to apply to the subpopulation as a whole (Aars et al. 2006; Obbard et al. 2010; Fig. 1). Nine of these subpopulation inventories (Baffin Bay “BB,” Davis Strait “DS,” Foxe Basin “FB,” Gulf of Boothia “GB,” Kane Basin “KB,” Lancaster Sound “LS,” M'Clintock Channel “MC,' Norwegian Bay “NW,” Viscount Melville Sound “VM”) covered essentially all of the area used by the subpopulation during the season of capture (Taylor et al. 2002, 2005, 2006a,b, 2008a,b, 2009; Peacock et al. 2011). These inventories were conducted by capture teams including territorial biologists and aboriginal hunters. The remaining four subpopulation inventories (Northern Beaufort “NB,” Southern Beaufort “SB,” Southern Hudson “SH” and Western Hudson “WH”) were conducted by provincial or federal agencies (i.e., Ontario Ministry of Natural Resources (MNR), United States Geological Survey (USGS) or Canadian Wildlife Services (CWS)), did not include aboriginal stakeholders as part of the regular capture teams, and did not capture polar bears throughout the entire subpopulation area (Regehr et al. 2006, 2007a,b; Obbard et al. 2007; Stirling et al. 2011).

In 2009, the International Union for Conservation of Nature/Species Survival Commission (IUCN/SSC) Polar Bear Specialists Group (PBSG) Status Report (Obbard et al. 2010) concluded that only 1 of 19 subpopulations is currently increasing, three are stable and eight are declining. For the remaining seven subpopulations, the 2009 PBSG concluded that the available data were insufficient to provide an assessment of trend (Obbard et al. 2010). Canada has or shares 13 of the 19 circumpolar subpopulations (Fig. 1), and the 2009 PBSG Status report lists Canada's subpopulations as: seven declining, four stable or increasing, and two data deficient. The Committee on the Status of Endangered Wildlife in Canada (COSEWIC) polar bear status report (Committee on the Status of Endangered Wildlife in Canada 2008) lists 7 of Canada's 13 subpopulations as stable/increasing, four as declining, and two as unknown. Vongraven and Richardson (2011) provide a status table “report card” that indicates that of 19 circumpolar subpopulations, seven are stable, five are increasing, and seven are data deficient. In December of 2013, the IUCN/SSC PBSG updated their status report listing 1 of 19 circumpolar subpopulations as increasing, five as stable, four as declining, and nine as data deficient (http://pbsg.npolar.no/en/status/status-table.html). For Canada's 13 subpopulations, the 2013 PBSG Status report lists one as increasing, five as stable, three as declining, and four as data deficient.

Until 2012, the PBSG considered PVA and TEK in the creation of their status reports. However, the PBSG status report did not consider PVA or TEK perspectives for their most recent status report (IUCN/PBSG, 2012). Rather they employed qualitative judgments based on the expert opinions of their members. The FB listing was changed from “data deficient” to “stable” based on recent aerial survey results indicating a stable/increasing trend (Stapleton et al. 2016). However, WH continues to be listed as “declining” in spite of a recent aerial survey that indicates no difference (trend not significant at *P*>0.05) and actually indicated a numerical increase (Stapleton et al. 2014). The SH subpopulation was listed as “stable” in 2009 in spite of PVA projections for decline and continues to be listed as stable, perhaps in response to the recent aerial survey that shows no change in numbers (Obbard et al. 2013). The DS subpopulation status was revised from “declining” to “stable” with no new research in DS to draw on. The LS and NW listings were also changed to “data deficient” based solely on the age of the subpopulation estimates of vital rates (IUCN/PBSG, 2013). Without a consistent (between subpopulations) rationale or consideration of all the information relevant to individual subpopulation status, the 2013 PBSG subpopulation status determinations are difficult to evaluate.

**Figure 2:**
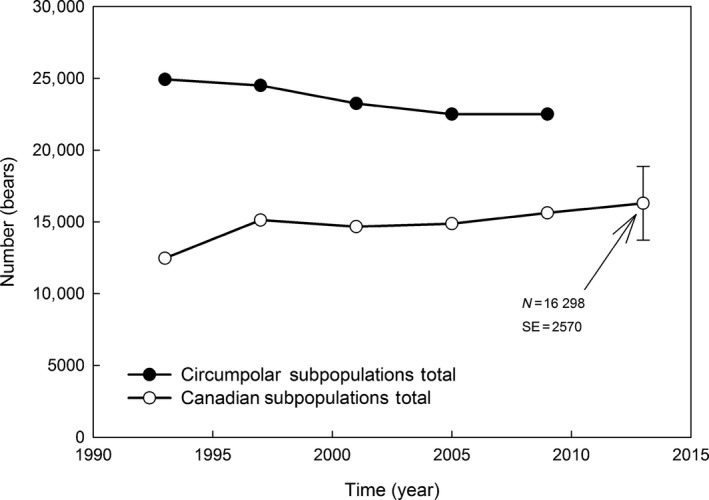
World and Canadian polar bear subpopulation trends for the 1993‐2013 period. Past estimates of abundance were taken from the International Union for Conservation of Nature Polar Bear Specialist Group (IUCN/PBSG) status reports (Wiig et al. 1993; Derocher et al. 1997a; Lunn et al. 2002; Aars et al. 2006; Obbard et al. 2010).

The main evidence for climate (reduced sea ice) effects on demography of subpopulations of polar bear is from the four subpopulations (NB, SB, SH, and WH) that were M‐R subsampled (Regehr et al. 2006, 2007a,b; Obbard et al. 2007; Stirling et al. 2011). The only subpopulation in Canada (or the world) where a decline was supposedly documented was the WH subpopulation (Regehr et al. 2007a,b). However, recent aerial surveys (Stapleton et al. 2016; Obbard et al. 2013) indicate that the SH and WH subpopulations have not declined, suggesting that SH and WH demographic rates and subpopulation numbers were under‐estimated by the previous M‐R work. The time series of scientific estimates of the circumpolar population and the Canadian subpopulations (Fig. 2) provide no support for a contemporary polar bear crisis (Wiig et al. 1993; Derocher et al. 1997a; Lunn et al. 2002; Aars et al. 2006; Obbard et al. 2010).

One measure of uncertainty in contemporary assessments of climate effects on polar bears is the divergence between scientific perspectives and aboriginal TEK on current subpopulation status (Dowsley 2005, 2007; Tyrrell 2006; Dowsley and Wenzel 2008; Henri et al. 2010; Lemelin et al. 2010). We summarize the demographic and TEK perspectives on the current status of all 13 Canadian subpopulations and explore reasons why the two perspectives differ for some subpopulations.

### Aboriginal right to hunt polar bear

To Inuit and First Nations, polar bears and polar bear hunting are an integral part of their culture and an important part of their traditional economy. Polar bears have been an integral part of the northern traditional economy since the fur trade expanded to the Canadian North in the early 20th century (Honderich 1991; Dowsley 2009b; Wenzel 2011). Aboriginal people (Inuit and First Nations) retain the right to harvest wildlife as both treaty and land claim rights, so long as their harvest is not a conservation concern. The Agreement on the Conservation of Polar Bears (ACPB) (Agreement on the Conservation of Polar Bears 1973) has been in effect since the mid‐1970s. The ACPB recognizes the traditional right to hunt and use polar bears by the indigenous societies of the signatory states. Inappropriate and unnecessary harvest and trade restrictions on polar bears wrongfully reduce the benefits of this cultural and economic resource for northern indigenous peoples (Wenzel 2011).

### Conservation threats to polar bears

Historically, the main conservation threat to polar bears was agreed to be hunting (Prestrud and Stirling 1994). In 2005, the IUCN/SSC Polar Bear Specialists Group (PBSG) recommended up‐listing the IUCN Red Book status to “threatened” based on a concern that declining sea ice might reduce polar bear stocks as much as 30% over three generations estimated to be 45 years, The Center for Biological Diversity (CBD) formally petitioned the US Fish and Wildlife Service (USFWS) to consider polar bears as a threatened species under the US Endangered Species Act (ESA) (Siegel and Cummings 2005). The CBD did not suggest that polar bears were in jeopardy from hunting practices; rather, they alleged that anthropogenic global warming and subsequent sea ice reduction was reducing polar bear habitat range wide. The CBD petition led to a USFWS range‐wide status review (Schliebe et al. 2006) which accepted uncritically the IPCC forecast for climate warming due to greenhouse gas emissions over the next century under expected emissions scenarios (Intergovernmental Panel on Climate Change 2007). The USGS produced a series of reports in 2007 in support of up‐listing polar bears to “threatened” status (Obbard et al. 2006; Amstrup et al. 2007; Hunter et al. 2007; Regehr et al. 2007a,b; Stirling et al. 2007). The US identified polar bears throughout their range as a “threatened” ESA “species at risk” in May of 2008 (US Department of the Interior: Fish and Wildlife Service 2008; Dowsley 2009b). Up‐listing had the effect of ending polar bear guided sport hunts from the US in Canada because of a provision of the US Marine Mammal Act (MMA) that automatically designates a US ESA “threatened” species as a MMA “depleted” species and importation is banned (Wenzel 2011). The US sport hunt in Canada was a quota‐based hunt, with quotas based on scientific estimates of sustainable yield (Freeman and Wenzel 2005). This event resulted in the annual loss of about 1.5–2 million dollars into Inuit traditional economy in Nunavut and the Northwest Territories (NWT) (Dowsley 2009b; Wenzel 2011). Canada's COSEWIC assessed polar bears in 2008 as a species of “special concern” in Canada, which was no change from the previous three designations (Committee on the Status of Endangered Wildlife in Canada 2008) using the correct generation time of 12 years.

There is no trend evident from the summed subpopulation numbers from the PBSG status reports (Fig. 2). Other indications of individual subpopulation decline are in conflict with aerial survey results, TEK, or subject to sampling ambiguity, with the exception of the KB subpopulation. We hypothesize that when polar bear subpopulation trends are evaluated by both M‐R sampling and TEK; notable differences are most likely due to errors in scientific methodology rather than mistaken TEK.

### Traditional ecological knowledge and scientific knowledge

Both Science and TEK consider that there is one underlying reality; and both knowledge systems are evidentiary (empirical) and experimental (interpretations of observations are tested and validated or rejected). Historically, the critical experiment for TEK holders was the employment of their knowledge in the various life sustaining activities that their survival depended on. The validation of TEK as a knowledge system was the persistence of both the people and the culture that provided the ontological context of the “traditional” knowledge. The term “science” can refer to both the body of knowledge produced by scientific inquiry and also the process of scientific inquiry itself. According to the Oxford English Dictionary, science is “a method or procedure consisting in systematic observation, measurement, and experiment, and the formulation, testing, and modification of hypotheses” (Oxford Dictionaries n.d.). Within that framework, there is a diversity of opinion regarding the appropriate way to do science (e.g., is induction allowed?), and thus there can be a lack of consensus on what does and does not constitute reliable scientific information. Similarly, there is diversity of opinion on what TEK is and how it should be documented and used (Cruikshank 1981; Usher 2000; Houde 2007; Dowsley and Wenzel 2008; Armitage and Kilburn 2015). Both systems sometimes make mistakes. Perhaps the most apparent difference between Science and TEK is that Science is held to be neutral, balanced and objective; while there are strong arguments that TEK cannot be fully understood outside the cultural context that developed and holds it (Usher 2000; Wenzel 2004; Dowsley and Wenzel 2008; Armitage and Kilburn 2015). However, some contemporary environmental scientists feel that researchers have a “moral responsibility” to advocate for the species and ecosystems they study. This view argues that advocacy is a duty because man has become the dominant species on earth and because man has impacted ecosystem services and reduced biodiversity (Chan 2008; Sodhi and Ehrlich 2011). When scientists are also environmental activists, can the scientific information provided be fully understood without considering the values of the science providers and the institutions that review and validate their work? Identification of an authoritative and generally accepted definition of Science and TEK is beyond the scope of this study. However, it is possible to compare the two systems empirically when the comparison is appropriately limited (Dowsley and Wenzel 2008). We limit our comparison to a single measure: the perception that a particular subpopulation of polar bears is increasing, declining or remaining relatively constant. We limit our scientific perspectives to polar bear subpopulations where M‐R sampling has been sufficient to estimate population numbers and rates of birth and death. We use PVA methods described below and the reported human removals to calculate the population trajectories of these subpopulations. We limit our consideration of TEK to Type I information (Usher 2000) on the population trend of polar bears in these same subpopulations. We follow Huntington's (2005) advice to focus most on the information provided rather than limiting ourselves to a single methodological protocol.

Our purpose is not to use Science to test TEK to determine whether it is accurate or not. We are also not testing Science to see whether it is accurate according to TEK standards. We compare scientific and TEK perspectives on polar bear subpopulation status to identify which subpopulations the two knowledge systems agree on, and which subpopulations they differ on. Secondarily, we look for any patterns in the agreed and contested comparisons that could explain why the two knowledge systems might not agree in some cases, and thus better understand what the true status of the various polar bear subpopulations might be. We ask whether there is any benefit (increased understanding of that one underlying reality) to asking why TEK agrees with Science on the status of some polar bear subpopulations, but disagrees with science on the status of others.

## Methods

### Subpopulation summaries

Subpopulation summaries were abstracted from the 2008 COSEWIC Polar Bear Status Report (Committee on the Status of Endangered Wildlife in Canada 2008) and 2009 PBSG Status Report (Obbard et al. 2010) and augmented/updated as required (S1). Methodologies for the PVA and TEK data considered for designations of polar bear subpopulation status are provided in the sections below.

### Traditional ecological knowledge

TEK relating to Canadian polar bear subpopulations is summarized from two types of sources. The first includes peer‐reviewed academic literature (e.g., Stirling and Andriashek 1992; Van De Velde et al. 2003; Tyrrell 2006; Dowsley 2007; Dowsley and Wenzel 2008; Lemelin et al. 2010) government reports (Farquharson 1976; Riewe 1976; CWS, 2009; PBTC, 2014; Joint Secretariat, 2015) or other commissioned reports or student theses (e.g., McDonald et al. 1997; Keith et al. 2005; Keith and Arqviq 2006; Committee on the Status of Endangered Wildlife in Canada 2008; Slavik 2009; 2009 PBSG Status Report (Obbard et al. 2010); Ghazal 2013; Slavik 2013; York 2014; York et al. 2015). The majority of these sources compiled TEK data through semi‐structured interviews focused on polar bears or related topics (such as ice conditions or hunting territories) with individuals selected by the communities as knowledgeable about polar bears or hunting more generally. This method is well established in TEK studies across the Arctic (Huntington 1998, 2000; Huntington et al. 2004). Two other methods were used in collecting data for this first set of sources. The first was the use of random phone interviews (used only in Kotierk 2010a,b); a method well established in social science research to collect general opinions of a population (Glasser and Metzger 1972). The other method was analytical workshop discussions (Calder 1977; Chambers 1994; Huntington 2005). This was used for the NTI (2007) report to collect information from a group of knowledge holders in Western Hudson Bay polar bear population area, and also used to develop the 12 Nunavut Polar Bear Memoranda of Agreement that identified sustainable harvest levels for 12 of Canada's 13 subpopulations. While this method may produce similar results to the semi‐structured interview, participant responses are sometimes constrained by the activity (e.g., MOUs) or dissenting views may be under‐represented in group scenarios (e.g., workshops).

The second type of TEK sources are summary reports (e.g., COSEWIC, 2008; PBTC, 2014), minutes of management meetings (Dowsley 2005; Dowsley and Taylor 2006a,b; Kativik Regional Government et al., 2010), and recorded comments made during interviews that were focused on other topics (Parks Canada, 2004). The TEK in these reports comes from individuals selected by their communities to represent the community at wildlife management and co‐management meetings. These individuals are typically the same acknowledged TEK holders that are interviewed in more structured studies. It is also relevant that that although there are typically some individuals who hold minority views on some TEK topics, one distinguishing feature of TEK is that it is held collectively rather than individually (Ottawa Traditional Knowledge Principles). Although summarized and incidental TEK may not be as robust as literature/report/workshop‐based information, we found no inconsistencies (other than individual variance) in the predominate TEK on polar bear subpopulation trend within or between these two TEK data types.

### Population viability analysis

Population viability analysis (PVA) is a widely applied methodology in conservation biology that has proven to be a useful tool for predicting the risks of extinction for a species or to evaluate the effectiveness of alternative management options, including harvest rates (Brook et al. 2000; Taylor et al. 2003a,b). Subpopulation estimates, recruitment rate estimates, survival rate estimates, and mean annual anthropogenic removals of both males and females were taken from the most recent published and literature and internal reports, including other status reports (COSEWIC, 2008; Taylor and Dowsley 2008; Obbard et al. 2010), specialist group minutes (Obbard et al. 2010; PBTC, 2011, 2012, 2013), academic presentations (IBA, 2011), and agency reports (S2, S3, S4). Sources for subpopulation‐specific estimates of abundance, survival, and recruitment are summarized in Table 1. Subpopulation five‐year mean annual anthropogenic removals were taken from Canadian Federal/Provincial Polar Bear Technical Meeting minutes (PBTC, 2008, 2009, 2010, 2011, 2012, 2013) and IUCN/SSC PBSG minutes (Aars et al. 2006; Obbard et al. 2010) that included harvest, defense, illegal, and accidental kills from all jurisdictions that shared a subpopulation (S4). The sex and age distribution of the harvest was estimated from long‐term harvest records unless a change in the historic sex/age distribution was indicated for an extended (>5 year) period.

**Table 1:** Sources for Canadian polar bear subpopulation‐specific estimates of abundance, survival, and recruitment

Subpop.	Source	Year of estimate	Recruitment rate estimate	Survival rate estimate	Estimate of abundance
Baffin Bay	Taylor et al. (2005)	1997	X	X	X
	Peacock et al. (2012)	2009		X	
Davis Strait	Peacock et al. (2013)	2007	X	X	X
Foxe Basin	Taylor et al. (2006b)	1994			X
	Stapleton et al. (2016)	2010			X
Gulf of Boothia	Taylor et al. (2009)	2000	X	X	X
Kane Basin	Taylor et al. (2008a)	1997	X	X	X
Lancaster Sound	Taylor et al. (2008b)	1997	X	X	X
M'Clintock Channel	Taylor et al. (2006a)	2000	X	X	X
Northern Beaufort Sea	Stirling Sodhi and Ehrlich (2011)	2006		X	X
	PBTC (2007)	N/A	X		
Norwegian Bay	Taylor et al. (2008b)	1997	X	X	X
Southern Beaufort Sea	Regehr et al. (2006)	2006	X	X	X
Southern Hudson Bay	Obbard et al. (2007)	2005		X	X
	PBTC (2007)	N/A	X		
	Obbard et al. (2013)	2012			X
Viscount Melville Sound	Taylor et al. (2002)	1999	X	X	X
Western Hudson Bay	Regehr et al. (2007a,b)	2004		X	X
	Stapleton et al. (2016)	2011			X
	PBTC (2007)	N/A	X		

### RISKMAN population viability analysis

We used RISKMAN version 2.0 PVA software (Taylor et al. 2003a,b) to estimate subpopulation trajectories and the probability of decline for each of Canada's 13 polar bear subpopulations under both harvested and unharvested scenarios. RISKMAN is a stochastic, (based on White 2000) demographic, individual‐based, age‐structured simulation model that was written as an explicit description of the three‐year reproduction cycle of polar bears (Taylor et al. 2002, 2005, 2006a,b; Taylor et al. 2008a,b, 2009; Peacock et al. 2013). Taylor et al. (2009) found that the proportion of total variance in survival rates that was parameter (as opposed to environmental) variance was estimated at approximately 92% for adults, 80% for subadults, and 100% for cubs. The nonparametric estimates for recruitment parameters did not allow partitioning parameter and environmental variance, and the only M‐R study to partition environmental and survival variance was the Gulf of Boothia (Taylor et al. 2009) study. Barber and Iacozza (2004) found no trends in Gulf of Boothia (GB) sea ice conditions or ringed seal habitat suitability indices in the interval 1980–2000, so the relative proportion of environmental variation may be reduced relative to other subpopulations or to contemporary conditions. We assumed that 75% of total uncertainty was due to parameter variance and 25% was due to environment variance for all subpopulations. We examined the sensitivity of subpopulation growth rate to how total variance was apportioned. Estimates of co‐variance were not available for most of the survival and recruitment estimates, and RISKMAN does not have the capacity to incorporate co‐variance estimates in stochastic simulations. However, RISKMAN does have a toggle that allows the user to assume independence (correlation coefficient (*R*) = 0) or complete positive correlation (correlation coefficient (*R*) = 1). We examined results for the bracketing cases (*R* = 0 and *R* = 1) to evaluate the effect of assuming independence in our simulations.

### Estimate of 2013 abundance and standing age distribution

We ran 5000 Monte Carlo iterations to obtain a distribution of subpopulation trajectories that extended from the last published subpopulation estimate to the present (i.e., 2013). We used mean harvest levels for the pre‐2013 simulation interval except for BB and NW where there was a qualitative change in harvest regime that identified two discrete intervals and required a stepwise simulation with interval‐specific mean harvest levels for the BB and NW pre‐2013 simulations. The resulting subpopulation number was used as the current estimate of abundance (*N*_2013_), and the resulting sex/age distribution was used as the 2013 standing age distribution for post‐2013 simulations. The standard error (SE) of the *N*_2013_ estimate was the standard deviation (SD) of the 5000 Monte Carlo iteration results for 2013.

### Subpopulation growth rates

We ran both deterministic and stochastic (5000 Monte Carlo iterations) simulations for a *t* = 20‐year period initiated with the estimated 2013 standing age distribution, and using the 2013 estimate of abundance (*N*_2013_) for the initial conditions for each subpopulation. For harvest simulations, we assumed the mean harvest level from the past 5 years (2007/2008–2011/2012) (S4: Table S1c) continued for that interval, and the harvested annual subpopulation geometric mean growth rates (*λ*_H_) were determined using the stochastic model. The geometric mean subpopulation growth rate for all subpopulations can be estimated as both the geometric mean Monte Carlo *λ*_t_ and also the *λ* that satisfied: *λ* ^t^ = *N*_t_/*N*_0_, where *N*_t_ and *N*_0_ were the Monte Carlo simulation mean values. We also monitored the number of Monte Carlo runs that were truncated for each subpopulation simulation. Our protocol for reporting geometric subpopulation growth rates when some Monte Carlo iterations were truncated is described below.

For some subpopulations (NB, SB, SH, WH), only total (includes harvest mortality) survival estimates were provided (Regehr et al. 2006, 2007a,b; Obbard et al. 2007; Stirling et al. 2011). For these subpopulations, simulations using “total survival” (includes harvest) rates were also conducted for a comparison to simulations using natural survival rates and annual harvest removals. Subpopulation‐specific total and harvest mortality rates were provided by various status reports (Committee on the Status of Endangered Wildlife in Canada 2008; Obbard et al. 2010), allowing us to estimate natural mortality and thus natural survival rates for these total (TOT) survival subpopulations.

Simulations were run for a 20‐year period because the long‐term standing sex/age distribution implications inherent for sex‐selective harvest of polar bears sometimes require 15+ years to become apparent (Taylor et al. 2008c). Our subpopulation status assessments are time referenced to 2013 and are based on the most recent subpopulation survival (S2: Tables S1 and S2) and recruitment rates (S3: Table S1) and 5‐year harvest rate averages (S4: Table S1c). This protocol does not imply that we believe that model projections are valid for 20 years or any specific time frame. The 2013 projection values provide an objective prediction of numbers and trend assuming that: (1) the initial or updated subpopulation estimate is unbiased (correct), (2) harvest numbers and demographic rate (SE) estimates are correct for the simulation interval to 2013, and (3) both harvest and demographic rates remain constant for the simulation interval beyond 2013.

### Subpopulation status and probability of decline

Our metric for subpopulation status was the unmodified (no expert correction) probability of decline over the simulation interval. The frequency of subpopulation simulations that declined over 20 years divided by the total number of Monte Carlo iterations was reported as the probability of decline. For subpopulations with total survival estimates (NB, SB, SH, WH), the probability of decline was also estimated with nonharvest simulations using “total” (includes harvest) rather than “natural” survival rate estimates.

### Subpopulation viability

To examine the current (2013) viability of each subpopulation under the most optimistic scenario (assuming no human removals), we ran both deterministic and stochastic (5000 Monte Carlo iterations) simulations initiated at stable‐age distribution using an initial total subpopulation of 10,000 (SE = 0). We report the mean geometric subpopulation growth rate for both deterministic and stochastic simulations where there are no human removals (zero harvest).

### “Truncated” Designation

We monitored the number and proportion of iteration runs that were truncated (*N*_t_ set to 0 when *N*_t_ ≤ 0). Truncated runs caused estimates of subpopulation growth rate *λ* to be biased (S5). Truncations occurred for a number of reasons. One cause was the occurrence of the initial random deviate for *N*_0_ ≤ 0. Another cause for truncation was when all individuals were lost to mortality (individual‐based model) or when the subpopulation could no longer satisfy a set harvest number at the observed sex ratio (quota). Truncation during a run occurred most frequently in nonviable subpopulations (mean survival and recruitment rates were insufficient for subpopulation persistence even with zero harvest), when the harvest quota was unsustainable, and when the coefficient of variation for initial subpopulation numbers and vital rates was relatively high. All truncations were pooled as a single category regardless of the reason they occurred.

As mentioned above, we estimated the geometric mean subpopulation growth rate for all subpopulations as both the geometric mean Monte Carlo *λ* ^t^ and also the *λ* that satisfied: *λ* ^t^ = *N*_t_/*N*_0_; and recorded the proportion of simulation iterations that were truncated. We report Monte Carlo and “*N*‐based” estimates of *λ* only when there were no truncations in the subpopulation simulation interval because of concerns these estimates would be biased (S5). Our PVA status estimates are the proportion of runs that declined over the simulation interval which were not affected by truncations (S5).

### Correspondence of PVA trends to TEK perspectives and sampling protocols

We used TEK estimates of status and recent subpopulation estimates from aerial surveys as a consistency check on the PVA subpopulation status determinations based on M‐R data. A Fisher's exact test (Microsoft (n.d.)) was used to compare PVA trends from M‐R demographic studies of polar bear subpopulations that were entirely surveyed versus partially surveyed with TEK views on correspondence (to nature) versus noncorrespondence. A nonparametric Mann–Whitney *U*‐test (SPSS ©, 2011) was used to compare partially versus entirely surveyed subpopulation estimates of unharvested subpopulation growth rate (subpopulation viability) and subpopulation status (probability of decline) because the Mann–Whitney U‐test has greater efficiency than the t‐test on non‐normal distributions and probability distributions are non‐normal. We excluded KB from this analysis because abundance and survival estimates may have been under‐estimated by source‐sink dynamics and because of a known and long term over harvest (Committee on the Status of Endangered Wildlife in Canada 2008; Taylor et al. 2008a; Obbard et al. 2010). We excluded BB from the subpopulation status (probability of decline) portion of this test because of the over‐estimation of Greenland harvest numbers (S1). A hierarchical cluster analysis using Ward's method applying squared Euclidean Distance as the distance or similarity measure (SPSS ©, 2011) was used to investigate the relationship of unharvested subpopulation growth rate to the probability of decline for harvested subpopulations, and the slopes of points within clusters were calculated by least squares regression. Recent subpopulation estimates from aerial surveys of the FB, and SH, and WH subpopulations (Obbard et al. 2013; Stapleton et al. 2014, 2016) were considered as an independent test of the validity of the trends indicated by the simulations using the mark–recapture estimates. A two sample *z*‐test was used to compare the simulation results (using natural survival and actual harvest estimates) to aerial survey estimates for the FB (entire area sampled) and SH and WH (partial area sampled) subpopulations.

## Results

**Figure 3:**
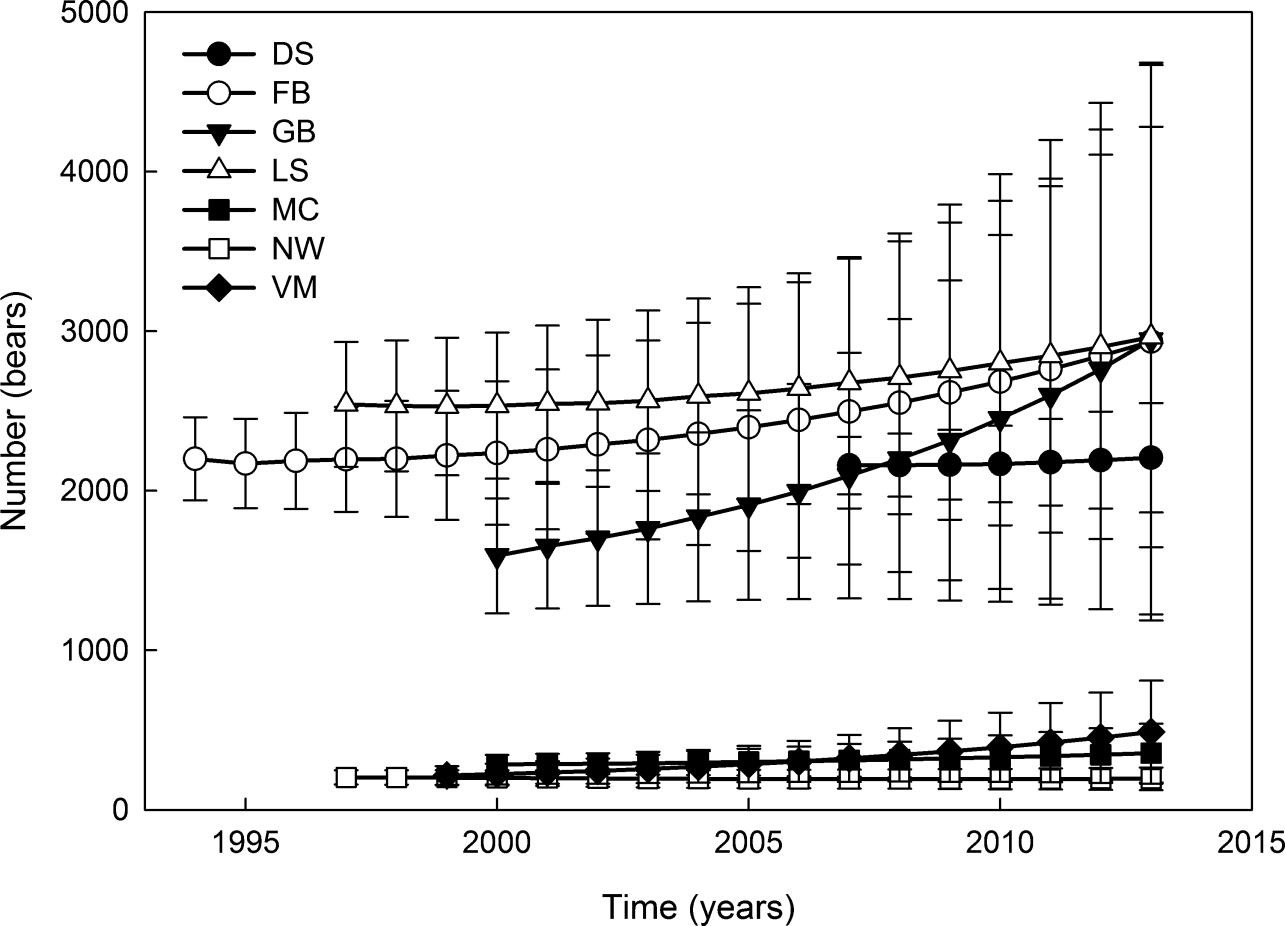
Stable/increasing subpopulation trajectories from the year of the most recent estimate of abundance to the present (2013). Davis Strait (DS), Foxe Basin (FB), Gulf of Boothia (GB), Lancaster Sound (LS), M'Clintock Channel (MC), and Viscount Melville Sound (VM) subpopulation trajectories (RISKMAN simulations) are time referenced to the year of the demographic estimate. Demographic estimates are from Peacock et al. (2013), Taylor et al. (2002, 2006a, 2006b, 2008b, 2009). Harvest numbers and the proportion of females in the harvest are provided in S4: Tables S1a, S1b, and S1c.

**Figure 4:**
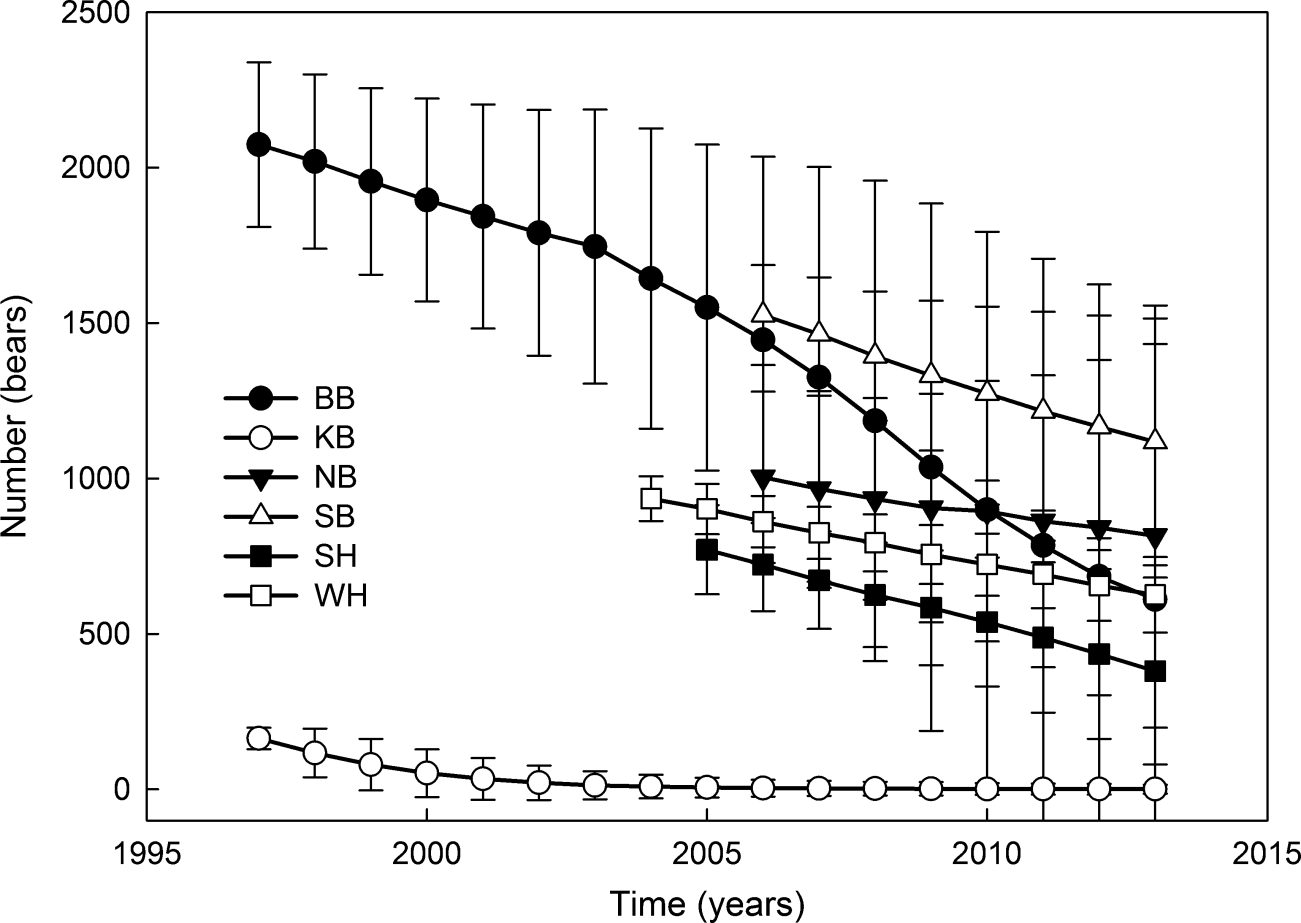
Declining subpopulation trajectories from the year of the most recent estimate of abundance to the present (2013). Baffin Bay (BB), Kane Basin (KB), Northern Beaufort Sea (NB), Norwegian Bay (NW), Southern Beaufort Sea (SB), Southern Hudson Bay (SH), and Western Hudson Bay (WH) subpopulation trajectories (RISKMAN simulations) are time referenced to the year of the demographic estimate. Demographic estimates are from Taylor et al. (2005, 2008a), Stirling Sodhi and Ehrlich (2011), Taylor et al. (2008b), Regehr et al. (2006), Obbard et al. (2007), and Regehr et al. (2007a,b). Harvest numbers and the proportion of females in the harvest are provided in S4: Tables S1a, S1b, and S1c.

Five‐year mean annual removals, proportion of removals that are female, subpopulation quotas, mean annual growth rates (and associated standard errors), the mean probabilities of decline (and associated standard errors), proportion of truncated runs, and TEK status summaries are listed for each subpopulation in Tables 2a and 2b. Seven of the 13 subpopulations (DS, FB, GB, LS, MC, NW, and VM) were identified as approximately stable or increasing (Tables 2b and 3; Fig. 3), while the remaining six (BB, KB, NB, SB, SH, and WH) were identified as declining (Tables 2b and 3; Fig. 4).

**Table 2a:** Estimates of abundance for polar bear subpopulations within or shared by Canada (BB – Baffin Bay, DS – Davis Strait, FB – Foxe Basin, GB – Gulf of Boothia, LS – Lancaster Sound, MC – M'Clintock Channel, NB – Northern Beaufort, NW – Norwegian Bay, SB – Southern Beaufort, SH – Southern Hudson Bay, VM – Viscount Melville Sound, and WH – Western Hudson Bay). Current estimates were generated using survival and recruitment rate estimates (S2: Tables S1 and S2; S3: Table S1), and harvest data from the PBTC for the period of the most recent abundance estimate to the 2011/2012 harvest season (S4: Tables S1a, S1b, and S1c)

Subpop.	Previous abundance estimate		Current abundance estimate			Human‐caused mortality		
	Year of estimate	N1 (SE)	N2 NAT (2013) (SE)^a^	N2 TOT (2013) (SE)^b^	Prop. of truncated runs	Permitted harvest (quota/year)^c^	5‐year mean harvest (bears/year)	Prop. female
BB^d^^,^ e ^,^ f	1997	2074 (265)	610.6418 (946.2684)	N/A	0.0008/0.6648	178 + Greenland	164	0.36
DS^g^^,^ h	2007	2158 (180)	2206.40 (342.8305)	N/A	0	54 + Quebec	81.2	0.36
FB^i^^,^ j ^,^ k	1994	2200 (260)	2934.90 (1748.80)/2697.60 (374.3645)^l^	N/A	0.0158/0.0036/0^l^	106 + Quebec	108.8	0.40
GB^m^	2000	1592 (361)	2945.7 (1722.0)	N/A	0.0052	74	59.8	0.39
KB^n^^,^ o	1997	164 (34.6)	0.6210 (14.4274)	N/A	0.9979	15	5	0.48
LS^p^	1997	2541 (391)	2963.5 (1316.8)	N/A	0.0076	85	84.6	0.31
MC^q^	2000	284 (59.3)	355.4872 (183.9414)	N/A	0	3	2.8	0.20
NB^r^	2006	1004 (275.5)	815.444 (616.8639)	555.104 (321.3018)	0.1088/0	65	32.4	0.41
NW^p^^,^ s	1997	203 (44)	194.3868 (70.6449)	N/A	0/0.0012	4	1.8	0
SB^t^^,^ u ^,^ v	2006	1526 (160.7)	1117.7 (396.8974)	1264.3 (404.8194)	0/0	80	36.8	0.33
SH^w^^,^ x	2005	771 (143.3)	380.6094 (300.4066)/937.9704(216.6548)	509.02 (66.9076)/899.5848 (192.7927)	0.2682/0.1626/0 0/0/0	55 + Quebec	57.2	0.33
VM^y^	1999	215 (57.5)	487.4612 (322.5756)	N/A	0.0402	7	4.4	0.19
WH^z^^,^ aa	2004	935 (72)	625.6672 (121.2577)/965.3274 (160.2103)	509.02 (60.9076)/880.0106 (136.16)	0/0/0/0/0/0	8 + Manitoba	21.6	0.33
Total	N/A	15 667 (796.3)	15 638.5190 (3091.3733)/16 298.2402 (2570.2325)^ab^	15 492.7538 (3035.8907)/16 060.7974 (2513.6153)^ab^	N/A	734	660.4	0.314

a N2 NAT (2013) is the 2013 estimate calculated using natural survival rates (S2: Table S2).

b N2 TOT (2013) is the 2013 estimate calculated using total survival rates (S2: Table S1).

c Maximum harvest that is presently allowed by jurisdictions with an identified quota, plus what is taken by nonquota jurisdictions.

d Taylor et al. (2005).

e The BB simulations used to determine a 2013 estimate of abundance were split into two separate trajectories (1: 1997–2003; 2: 2003–2013) to address a significant increase in the number of bears being harvested (S4).

f Dowsley and Wenzel (2008).

g Peacock et al. (2013).

h E. Peacock, unpubl. data.

i Taylor et al. (2006b).

j Comments at community consultations throughout Foxe Basin.

k Survival and recruitment rates were established as BB survival and recruitment (Taylor et al. 2005) except that FB adult litter production was 0.85 (see FB comments for meta‐analysis rationale).

l Simulations were also conducted using a recent aerial survey estimate from Stapleton et al. (2016).

m Taylor et al. (2009).

n Taylor et al. (2008a).

o Further simulations were not conducted because this subpopulation is clearly a harvest sink that can only persist from immigration from surrounding subpopulations.

p Taylor et al. (2008b).

q Taylor et al. (2006a).

r Stirling Sodhi and Ehrlich (2011).

s The NW simulations used to determine a 2013 estimate of abundance were split into two separate trajectories (1: 1997–2004; 2: 2004–2013) to address the absence of females in the harvest after the 03/04 harvest season (S4).

t Regehr et al. (2006).

u Hunter et al. (2007).

v Rode et al. (2007).

w Obbard et al. (2007).

x Simulations were also conducted using a recent aerial survey estimate from Obbard et al. (2013).

y Taylor et al. (2002).

z Regehr et al. (2007a, b.

aa Simulations were also conducted using a recent aerial survey estimate from Stapleton et al. (2016).

ab The 2013 Canadian polar bear population estimate was corrected to account for the recent aerial survey estimates (13).

**Table 2b:** The reported TEK status and the PVA probability of decline for each Canadian polar bear subpopulation were examined to determine subpopulation status. We also included the proportion of runs that were truncated during post‐2013 simulations for each Canadian subpopulation. Post‐2013 harvested subpopulation growth rates were not reported because truncations are known to bias estimates of subpopulation growth rates (S5). Post‐2013 simulations were run for a 20‐year period using the 2007/2008–2011/2012 mean annual removals (Table E3) to determine the probability of decline

Subpop.	Post‐2013 simulation results		TEK Reported Status
	PVA probability of decline (SE)	Prop. of truncated runs	
Baffin Bay	0.934 (0.0035)	0.9176	Abundant/Stable/Increasing^a^
Davis Strait	0.3894 (0.0069)	0.0056	Abundant/Stable/Increasing^j^
Foxe Basin	0.2892 (0.0064)/0.2224 (0.0059)^a^	0.180/0.0054^a^	Abundant/Increasing^c^
Gulf of Boothia	0.2016 (0.0057)	0.107	Abundant/Stable/Increasing^a^
Kane Basin	N/A	N/A	Overhunted/Declining^c^
Lancaster Sound	0.3632 (0.0068)	0.1312	Abundant/Stable^b^
M'Clintock Channel	0.3178 (0.0066)	0.0458	Recovering/Increasing^c^
Northern Beaufort Sea	0.8348 (0.0053)/0.9344 (0.0035)^f^	0.7328/0^f^	Abundant/Stable^d^
Norwegian Bay	0.4034 (0.0069)	0.0106	Low Density/Stable^e^
Southern Beaufort Sea	0.889 (0.0044)/0.779 (0.0059)^f^	0.5008/0^f^	Abundant/Stable^f^
Southern Hudson Bay	0.9816 (0.0019)/0.8772 (0.0046)^g^/0.0910 (0.0041)^f^/0.9586 (0.0028)^f^^,^ g	0.9696/0.7586^g^/0^f^/0^f^^,^ g	Abundant/Stable^h^
Viscount Melville Sound	0.1884 (0.0055)	0.1106	Recovering/Increasing^h^
Western Hudson Bay	0.9954 (0.0010)/0.9766 (0.0021)^i^/1 (0)^f^/1 (0)^f^^,^ i	0.747/0.1554^i^/0^f^/0^f^^,^ i	Abundant/Stable/Increasing^j^

The BB TEK status was summarized from Dowsley (2005, 2007, 2010), Dowsley and Taylor (2006a), Committee on the Status of Endangered Wildlife in Canada (2008), Dowsley and Wenzel (2008), Canadian Wildlife Service (2009), Polar Bear Technical Committee (2014).

The DS TEK status was summarized from Brice‐Bennett (1976), McDonald et al. (1997), Committee on the Status of Endangered Wildlife in Canada (2008), Canadian Wildlife Service (2009), Kativik et al. (2010), Kotierk (2010a,b), Polar Bear Technical Committee (2014).

Simulations were also conducted using a 2013 estimate simulated from a recent aerial survey estimate from Stapleton et al. (2016).

The FB TEK status was summarized from McDonald et al. (1997), Van De Velde et al. (2003), Keith and Arqviq (2006), Committee on the Status of Endangered Wildlife in Canada (2008), Canadian Wildlife Service (2009), Ghazal (2013), Polar Bear Technical Committee (2014),

a The GB TEK status was summarized from Van De Velde et al. (2003), Keith et al. (2005), Keith and Arqviq (2006), Committee on the Status of Endangered Wildlife in Canada (2008), Canadian Wildlife Service (2009), Polar Bear Technical Committee (2014).

The KB TEK status was summarized from Committee on the Status of Endangered Wildlife in Canada (2008), Canadian Wildlife Service (2009), Polar Bear Technical Committee (2014).

b The LS TEK status was summarized from Keith and Arqviq (2006), Committee on the Status of Endangered Wildlife in Canada (2008), Canadian Wildlife Service (2009), Polar Bear Technical Committee (2014).

c The MC TEK status was summarized from Keith et al. (2005), Keith and Arqviq (2006), Committee on the Status of Endangered Wildlife in Canada (2008), Canadian Wildlife Service (2009), Polar Bear Technical Committee (2014),.

Simulations were also conducted using total survival rates (S2: Table S1).

d The NB TEK status was summarized from Stirling and Andriashek (1992), Parks Canada (2004), Committee on the Status of Endangered Wildlife in Canada (2008), Canadian Wildlife Service (2009), Slavik (2009), Polar Bear Technical Committee (2014).

e The NW TEK status was summarized from Riewe (1976), Committee on the Status of Endangered Wildlife in Canada (2008), Canadian Wildlife Service (2009), Polar Bear Technical Committee (2014).

f The SB TEK status was summarized from Stirling and Andriashek (1992), Committee on the Status of Endangered Wildlife in Canada (2008), Slavik (2009), Polar Bear Technical Committee (2014).

g Simulations were also conducted using a 2013 estimate simulated from a recent aerial survey estimate from Obbard et al. (2013).

The SH TEK status was summarized from McDonald et al. (1997), Committee on the Status of Endangered Wildlife in Canada (2008), Canadian Wildlife Service (2009), Kativik et al. (2010), Lemelin et al. (2010), Polar Bear Technical Committee (2014).

h The VM TEK status was summarized from Farquharson (1976), Committee on the Status of Endangered Wildlife in Canada (2008), Canadian Wildlife Service (2009), Polar Bear Technical Committee (2014).

i Simulations were also conducted using a 2013 estimate simulated from a recent aerial survey estimate from Stapleton et al. (2016).

j The WH TEK status was summarized from McDonald et al. (1997), Dowsley and Taylor (2006b), Tyrrell (2006), Nunavut Tunngavik Incorporated (2007), Committee on the Status of Endangered Wildlife in Canada (2008), Dowsley and Wenzel (2008), Canadian Wildlife Service (2009), Dowsley (2010), Polar Bear Technical Committee (2014).

**Table 3:** The following table compares two methods for identifying subpopulation status. The first is based strictly on PVA, and the second is based strictly on TEK. We also propose a third method which is based on a correspondence between both PVA and TEK, where when they do not agree the status is considered to be “uncertain”. We also provide a summary of the primary evidence considered for each subpopulation

Subpopulation	PVA Results	TEK	Trend	Primary Evidence
Baffin Bay	Declining	Stable/Increasing	Uncertain	M‐R/TEK
Davis Strait	Stable/Increasing	Stable/Increasing	Stable/Increasing	M‐R/TEK
Foxe Basin	Stable/Increasing	Stable/Increasing	Stable/Increasing	Aerial Survey/M‐R/TEK
Gulf of Boothia	Stable/Increasing	Stable/Increasing	Stable/Increasing	M‐R/TEK
Kane Basin	Declining	Declining	Declining	M‐R/TEK
Lancaster Sound	Stable/Increasing	Stable/Increasing	Stable/Increasing	M‐R/TEK
M'Clintock Channel	Stable/Increasing	Stable/Increasing	Stable/Increasing	M‐R/TEK
Northern Beaufort Sea	Declining	Stable/Increasing	Uncertain	M‐R/TEK
Norwegian Bay	Stable/Increasing	Stable/Increasing	Stable/Increasing	M‐R/TEK
Southern Beaufort Sea	Declining	Stable/Increasing	Uncertain	M‐R/TEK
Southern Hudson Bay	Declining	Stable/Increasing	Uncertain	Aerial Survey/M‐R/TEK
Viscount Melville Sound	Stable/Increasing	Stable/Increasing	Stable/Increasing	M‐R/TEK
Western Hudson Bay	Declining	Stable/Increasing	Uncertain	Aerial Survey/M‐R/TEK

The trend estimate employing total survival estimates for each of these subpopulations was qualitatively the same as those using natural survival estimates and observed mean annual removal values (Table 2a). The range of deviance between the 2013 estimates of abundance based on natural survival rates to the 2013 estimates using total survival rates was 12.3–38.0%.

Three (NB, SB, and WH) of the six (BB, KB, NB, SB, SH, and WH) subpopulations that appeared to be declining also had demographic rate estimates insufficient for long‐term persistence (i.e., not viable subpopulations even with zero harvest) (Table 4). The SH subpopulation was projected as marginally viable but lacked sufficient productivity to sustain more than a miniscule fraction (one bear) of the historical annual kill (i.e., 48.625 bears per year) (Table 4). The projected decline in KB and projected stable/increase status for DS, FB, GB, LS, MC, NW, and VM were all consistent with TEK (Committee on the Status of Endangered Wildlife in Canada 2008; M. Taylor, pers. comm. 1986‐2008). TEK perspectives on subpopulation trend were in general agreement with 8 of Canada's 13 subpopulations, but differed from five of six that were projected to be declining (Tables 2b and 3). The probability that TEK status perspectives would differ more often from declining subpopulations than from stable/increasing subpopulations by chance was p<0.005 (Table 5). Similarly, a PVA versus TEK consistency comparison suggested that scientific perspectives on trend from subpopulations that had been partially surveyed were less likely to be supported by TEK (p<0.007) (Table 6).

**Table 4:** Canadian polar bear subpopulation viability based on PVA results generated from natural survival and recruitment rate estimates (S2: Tables S2; S3: Table S1). Each subpopulation was simulated from a stable‐age distribution from an initial subpopulation estimate of *N* = 10,000, SE = 0 for a 20‐year period under a harvest moratorium. The unharvested geometric subpopulation growth rate (*λ*_H = 0_), PVA probability of decline (*p*_decline_), and the number of truncations has been included

Subpopulation	Deterministic *λ*_H = 0_	Stochastic *λ*_H = 0_ (SE)	*p*_decline_ (SE)	TRUNC
Baffin Bay	1.0551	1.0547 (0.0274)	0.0026 (0.0007)	0
Davis Strait	1.0387	1.0385 (0.0175)	0.016 (0.0018)	0
Foxe Basin	1.0501	1.0491 (0.0196)	0.0076 (0.0012)	0
Gulf of Boothia	1.0646	1.0639 (0.0369)	0.0472 (0.0030)	0
Kane Basin	1.0064	1.0098 (0.0359)	0.4008 (0.0069)	0
Lancaster Sound	1.0247	1.0249 (0.0189)	0.0908 (0.0041)	0
M'Clintock Channel	1.0263	1.0245 (0.0345)	0.2054 (0.0057)	0
Northern Beaufort Sea	0.9947	0.9887 (0.0794)	0.5198 (0.0071)	0
Norwegian Bay	1.0077	1.0077 (0.0189)	0.3574 (0.0068)	0
Southern Beaufort Sea	0.9808	0.9795 (0.0415)	0.6734 (0.0066)	0
Southern Hudson Bay	1.0014	0.9999 (0.0397)	0.4876 (0.0071)	0
Viscount Melville Sound	1.0652	1.0621 (0.0426)	0.0732 (0.0037)	0
Western Hudson Bay	1.0004	0.9991 (0.0135)	0.5326 (0.0071)	0

**Table 5:** A Fisher's exact test comparison of Science versus TEK correspondence for PVA trends based on mark–recapture demographic studies of Canadian polar bear subpopulations declining suggested that scientific perspectives on trend from subpopulations that were declining were less likely to be supported by TEK (*P* < 0.005)

Sample Protocol	TEK Supports	TEK Disputed
Stable/Increasing	7	0
Declining	1	5

**Table 6:** A Fisher's exact test comparison of Science versus TEK correspondence for PVA trends based on mark–recapture demographic studies of Canadian polar bear subpopulations suggested that scientific perspectives on trend from subpopulations that had been partially surveyed were less likely to be supported by TEK (*P* < 0.007)

Sample Protocol	TEK Supports	TEK Disputed
Entire Subpopulation Area	8	1
Partial Subpopulation	0	4

**Figure 5:**
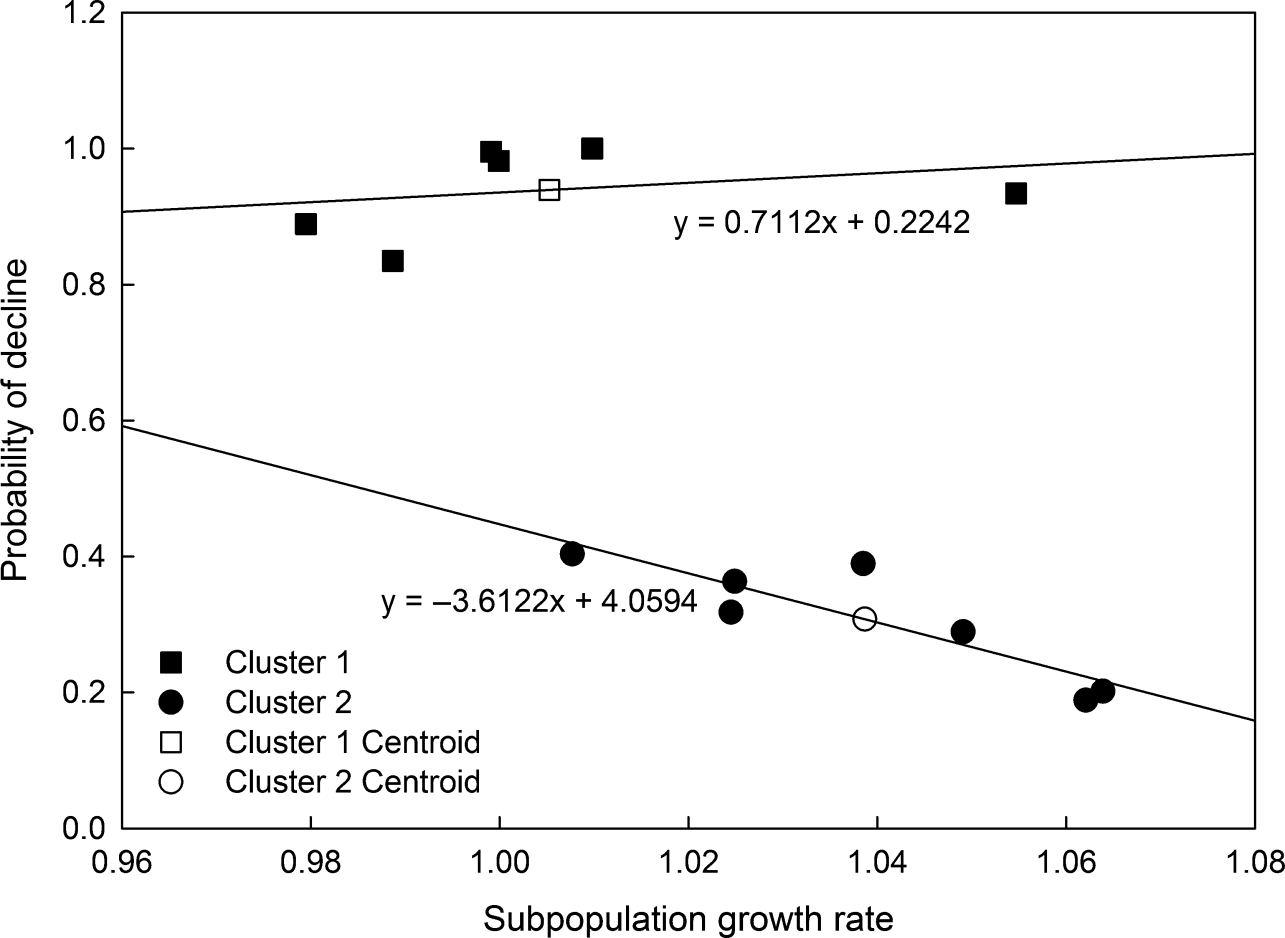
A hierarchical cluster analysis was used to investigate the relationship between probability of decline as estimated by population viability analysis (PVA) for harvested Canadian polar bear subpopulations and unharvested subpopulation growth rates. Two distinct clusters were identified (*P *≤ 0.027): Cluster 1 containing Baffin Bay (BB), Kane Basin (KB), Northern Beaufort Sea (NB), Southern Beaufort Sea (SB), Southern Hudson (SH), and Western Hudson Bay (WH); and Cluster 2 containing Davis Strait (DS), Foxe Basin (FB), Gulf of Boothia (GB), Lancaster Sound (LS), M'Clintock Channel (MC), Norwegian Bay (NW), and Viscount Melville Sound (VM).

Mann–Whitney *U*‐tests conducted using unharvested geometric subpopulation growth rates (Table 4) and the post‐2013 harvested probability of decline (Table 2b) revealed that unharvested subpopulation growth rates were less for subpopulations that had been partially sampled than for subpopulations that were entirely sampled (*P* ≤ 0.004), and PVA status assessments were more likely to indicate decline for subpopulations that had been partially sampled than for subpopulations that were entirely sampled (*P* ≤ 0.006) (Table 7). A hierarchical cluster analysis, based on subpopulation unharvested subpopulation growth rate (intrinsic productivity) and harvested subpopulation probability of decline (status), identified two distinct subpopulation clusters (Fig. 5) (post hoc *P* ≤ 0.027); Cluster one contains BB, KB, NB, SB, SH, WH, and Cluster two contains DS, FB, GB, LS, MC, NW, VM. The slopes within clusters were not significant, indicating no relationship between intrinsic productivity and probability of decline within clusters.

**Table 7:** Mann–Whitney *U*‐tests were used to compare distributions for the unharvested geometric subpopulation growth rate (*λ*_H = 0_) (Table 4), the probability of decline (*p*_decline_), and their associated rankings for partially and entirely mark–recapture sampled subpopulations (Table 2b). Estimates of unharvested subpopulation growth rate for *λ*_H = 0_ were lower (*P* ≤ 0.004), and estimates of the probability of decline for harvested subpopulations were higher (*P* ≤ 0.006) for partially sampled subpopulations

Subpopulation	*λ*_H = 0_	*λ*_H = 0_ Rank	*p*_decline_ (*λ*_H_)	*p*_decline_ Rank (*λ*_H_)	Sample protocol
Baffin Bay^a^	1.0547	3	0.9340	10	Entire
Davis Strait	1.0385	5	0.3894	6	Entire
Foxe Basin	1.0491	4	0.2892	3	Entire
Gulf of Boothia	1.0639	1	0.2016	2	Entire
Kane Basin^b^^,^ b	1.0098	8	1	13	Entire
Lancaster Sound	1.0249	6	0.3632	5	Entire
M'Clintock Channel	1.0245	7	0.3178	4	Entire
Northern Beaufort Sea	0.9887	12	0.8348	8	Partial
Norwegian Bay	1.0077	9	0.4034	7	Entire
Southern Beaufort Sea	0.9795	13	0.8890	9	Partial
Southern Hudson Bay	0.9999	10	0.9816	11	Partial
Viscount Melville Sound	1.0621	2	0.1884	1	Entire
Western Hudson Bay	0.9991	11	0.9954	12	Partial

a BB was excluded from the probability of decline portion of the Mann–Whitney *U*‐test.

KB was excluded from both Mann–Whitney *U*‐tests.

b Post‐2013 simulations for the KB subpopulation were not conducted because it was depleted by the 2013 estimate (*N* = 0; refer to Table 2a) and it appears to be a harvest sink that can only persist from immigration from surrounding subpopulations. Thus, a 1.0 probability of decline is assumed.

The sensitivity of simulation results to how total variance was partitioned and the effect of co‐variance were relatively minor (Tables 8 and 9).

**Table 8:** The effect of total variance settings on the geometric mean subpopulation growth rate (*λ* G) and probability of decline (*P*_decline_) was examined for each Canadian subpopulation. We examined the difference between total variance settings of 25% parameter variance/75% environmental variance; 100% parameter variance; and 100% environmental variance. Each subpopulation was simulated from a stable‐age distribution from an initial subpopulation estimate of *N* = 10,000, SE = 0 for a 20‐year period under a harvest moratorium. Canadian polar bear subpopulations are defined as: Baffin Bay (BB), Davis Strait (DS), Foxe Basin (FB), Gulf of Boothia (GB), Kane Basin (KB), Lancaster Sound (LS), M'Clintock Channel (MC), Northern Beaufort Sea (NB), Norwegian Bay (NW), Southern Beaufort Sea (SB), Southern Hudson Bay (SH), Viscount Melville Sound (VM), and Western Hudson Bay (WH)

Subpop.	Deterministic	100% Parameter			75% Parameter/25% Environmental			25% Parameter/75% Environmental			100% Environmental		
	*λ* G	*λ* G (SE)	*P*_decline_	TRUNC	*λ* G (SE)	*P*_decline_	TRUNC	*λ* G (SE)	*P*_decline_	TRUNC	*λ* G (SE)	*P*_decline_	TRUNC
BB	1.0551	1.0549 (0.0224)	0.0088 (0.0013)	0	1.0547 (0.0183)	0.0026 (0.0007)	0	1.0544 (0.0125)	0 (0)	0	1.0548 (0.0051)	0 (0)	0
DS	1.0387	1.0384 (0.0120)	0.0304 (0.0024)	0	1.0385 (0.0175)	0.016 (0.0018)	0	1.0383 (0.0111)	0.0004 (0.0003)	0	1.0385 (0.0048)	0 (0)	0
FB	1.0501	1.0492 (0.0220)	0.0044 (0.0009)	0	1.0491 (0.0196)	0.0076 (0.0012)	0	1.0496 (0.0120)	0 (0)	0	1.0494 (0.0054)	0 (0)	0
GB	1.0646	1.0664 (0.0412)	0.0584 (0.0033)	0	1.0639 (0.0369)	0.0472 (0.0030)	0	1.0622 (0.0269)	0.0202 (0.0020)	0	1.0640 (0.0093)	0 (0)	0
KB	1.0064	1.0103 (0.0395)	0.4202 (0.0070)	0	1.0098 (0.0359)	0.4008 (0.0069)	0	1.0059 (0.0238)	0.3978 (0.0069)	0	1.0049 (0.0110)	0.317 (0.0066)	0
LS	1.0247	1.0249 (0.0219)	0.1292 (0.0047)	0	1.0249 (0.0189)	0.0908 (0.0041)	0	1.0244 (0.0119)	0.0212 (0.0020)	0	1.0240 (0.0053)	0 (0)	0
MC	1.0263	1.0258 (0.0363)	0.2142 (0.0058)	0	1.0245 (0.0345)	0.2054 (0.0057)	0	1.0252 (0.0197)	0.1008 (0.0043)	0	1.0258 (0.0082)	0.0016 (0.0006)	0
NB	0.9947	0.9871 (0.0969)	0.521 (0.0071)	0.0006	0.9887 (0.0794)	0.5198 (0.0071)	0	0.9819 (0.0613)	0.5672 (0.0070)	0	0.9922 (0.0224)	0.6248 (0.0068)	0
NW	1.0077	1.0078 (0.0209)	0.356 (0.0068)	0	1.0077 (0.0189)	0.3574 (0.0068)	0	1.0074 (0.0114)	0.2598 (0.0062)	0	1.0071 (0.0050)	0.0796 (0.0038)	0
SB	0.9808	0.9788 (0.0482)	0.6428 (0.0068)	0	0.9795 (0.0415)	0.6734 (0.0066)	0	0.9798 (0.0257)	0.7834 (0.0058)	0	0.9797 (0.0109)	0.9706 (0.0024)	0
SH	1.0014	1.0003 (0.0442)	0.4682 (0.0071)	0	0.9999 (0.0397)	0.4876 (0.0071)	0	1.0006 (0.0247)	0.4804 (0.0071)	0	1.0005 (0.011)	0.4784 (0.0071)	0
VM	1.0652	1.0641 (0.0429)	0.0712 (0.0036)	0	1.0621 (0.0426)	0.0732 (0.0037)	0	1.0550 (0.0448)	0.1106 (0.0044)	0	1.0635 (0.0103)	0 (0)	0
WH	1.0004	0.9987 (0.0159)	0.5312 (0.0071)	0	0.9991 (0.0135)	0.5326 (0.0071)	0	0.9999 (0.0082)	0.5224 (0.0071)	0	1.0001 (0.0039)	0.4954 (0.0071)	0

**Table 9:** The effect of co‐variance *R* = 0 “independent” versus *R* = 1 “100% correlated” on the geometric mean subpopulation growth rate (*λ*_G_) and probability of decline (*P*_decline_) was examined for each Canadian subpopulation using total variance settings of 75% parameter variance/25% environmental variance. Each subpopulation was simulated from a stable‐age distribution from an initial subpopulation estimate of *N* = 10,000, SE = 0 for a 20‐year period under a harvest moratorium. Canadian polar bear subpopulations are defined as: Baffin Bay (BB), Davis Strait (DS), Foxe Basin (FB), Gulf of Boothia (GB), Kane Basin (KB), Lancaster Sound (LS), M'Clintock Channel (MC), Northern Beaufort Sea (NB), Norwegian Bay (NW), Southern Beaufort Sea (SB), Southern Hudson Bay (SH), Viscount Melville Sound (VM), and Western Hudson Bay (WH)

Subpop.	75% Parameter/25% Environmental; *R* = 0			75% Parameter/25% Environmental; *R* = 1		
	*λ*_G_ (SE)	*P*_decline_	TRUNC	*λ*_G_ (SE)	*P*_decline_	TRUNC
BB	1.0547 (0.0183)	0.0026 (0.0007)	0	1.0505 (0.0197)	0.0064 (0.0011)	0
DS	1.0385 (0.0175)	0.016 (0.0018)	0	1.0372 (0.0185)	0.0256 (0.0022)	0
FB	1.0491 (0.0196)	0.0076 (0.0012)	0	1.0503 (0.0187)	0.0046 (0.0010)	0
GB	1.0639 (0.0369)	0.0472 (0.0030)	0	1.0440 (0.0348)	0.0970 (0.0042)	0
KB	1.0098 (0.0359)	0.4008 (0.0069)	0	0.9685 (0.0365)	0.8074 (0.0056)	0
LS	1.0249 (0.0189)	0.0908 (0.0041)	0	1.0186 (0.0199)	0.1702 (0.053)	0
MC	1.0245 (0.0345)	0.2054 (0.0057)	0	1.0012 (0.0345)	0.4406 (0.0070)	0
NB	0.9887 (0.0794)	0.5198 (0.0071)	0	0.9292 (0.0699)	0.8626 (0.0049)	0
NW	1.0077 (0.0189)	0.3574 (0.0068)	0	1.0037 (0.0191)	0.4196 (0.0070)	0
SB	0.9795 (0.0415)	0.6734 (0.0066)	0	0.9751 (0.0404)	0.7168 (0.0064)	0
SH	0.9999 (0.0397)	0.4876 (0.0071)	0	0.9375 (0.0521)	0.9240 (0.0037)	0
VM	1.0621 (0.0426)	0.0732 (0.0037)	0	1.0495 (0.0415)	0.1018 (0.0043)	0
WH	0.9991 (0.0135)	0.5326 (0.0071)	0	0.9992 (0.0138)	0.5248 (0.0071)	0

## Discussion

The diversity of perspectives on the status of polar bears has never been greater or more polarized (Treseder and Carpenter 1989; Nageak et al. 1991; Prestrud and Stirling 1994; Obbard et al. 2010; Stirling and Derocher 2012). To some environmentalist Non‐Government Organizations (NGO) (e.g., Polar Bears International, CBD, World Wildlife Fund and Greenpeace), polar bears have become both an icon and poster species (Slocum 2004) for their efforts to influence governments and peoples to reduce carbon dioxide emissions, and thus limit or reduce the extent of anthropogenic global warming. To aboriginal people (Inuit and First Nations) polar bears and polar bear hunting remains an integral part of their culture; an important part of their traditional economy; and a constitutional, treaty and land claim right (Dowsley 2009b; Wenzel 2011). Although both groups agree that climate warming has caused a decline in sea ice, they disagree about what effects the changes in sea ice have had on polar bear numbers (Tables 2b and 3). Polar bear range states attempt to identify management policies that are responsive to both perspectives, but in practice, most polar bear management decisions are guided by agency researchers resulting in mainly science‐based policies (Obbard et al. 2010). This approach fails to reconcile when TEK and science are qualitatively different, or when there is concern that scientific perspectives are influenced by external concerns or if aboriginal perspectives are overly influenced by a desire to harvest more polar bears.

We suggest that the difference between scientific and TEK in this case is partly caused by institutional (science establishment) reluctance to accept TEK as a valid test of correspondence between scientific predictions and observable reality (Aars et al. 2006; Resolution # 1‐2005). We are aware that TEK, like science, has the potential to be incorrect (Gilchrist et al. 2005) and knowledge holders may not always be able to provide a clear consensus on a particular issue (Dowsley and Wenzel 2008). However, we did not find evidence for intentional misrepresentation of polar bear subpopulation numbers or trends from TEK, aboriginal organizations, or co‐management wildlife boards. The TEK we report for Canadian polar bear subpopulation trends was a consensus from all of the sources cited above, which we believe to be a comprehensive list of available sources on the TEK for polar bear subpopulation trends. Scientific studies have not always agreed with TEK on subpopulation trend (e.g., NB, SB, SH, and WH), but these studies have never provided any reasons to suspect that the available TEK was suspect or incorrect. Alternatively, we are aware of multiple occasions when TEK accurately identified polar bear subpopulation trends before new scientific studies had been conducted that corroborated the TEK (S6).

We suggest that the PVA evidence that polar bears are declining in the NB, SB, SH, and WH subpopulations may be unreliable because the M‐R sampling that these studies are based on was conducted in a manner that was inconsistent with the analysis model (Fletcher et al. 2012; Abadi et al. 2013). The most direct empirical evidence to support this contention is the recent Fall 2011 aerial surveys of the WH and SH subpopulations (Stapleton et al. 2016; Obbard et al. 2013) which documents an apparent increase (*p*_increaseWH_ ≥ 0.6767 and *p*_increaseSH_ ≥ 0.7876) for WH and SH polar bears in contrast to the M‐R results (Obbard et al. 2007; Regehr et al. 2007a,b) and various status reports (Committee on the Status of Endangered Wildlife in Canada 2008; Polar Bear Technical Committee (PBTC) 2009; Obbard et al. 2010; Vongraven and Richardson 2011). The WH subpopulation is often described as the “best known” or the “most intensively studied” (Derocher and Stirling 1995b:215; Stapleton et al. 2016:38) polar bear subpopulation. Regehr et al. (2007a,b) state that the WH polar bear subpopulation is in decline and that these (decline) results are reliable and require no qualification. For similar reasons, Ontario uplisted polar bears to “threatened” status under Ontario ESA (Ontario, 2007) in June 2009 (Committee on the Status of Species at Risk in Ontario 2009). However, TEK maintains that both the SH and WH subpopulation numbers have not declined (Tyrrell 2006; NTI, 2007; Lemelin et al. 2010). Recent aerial surveys (Stapleton et al. 2016; Obbard et al. 2013) support the local Inuit perspectives (i.e., no decline).

**Figure 6:**
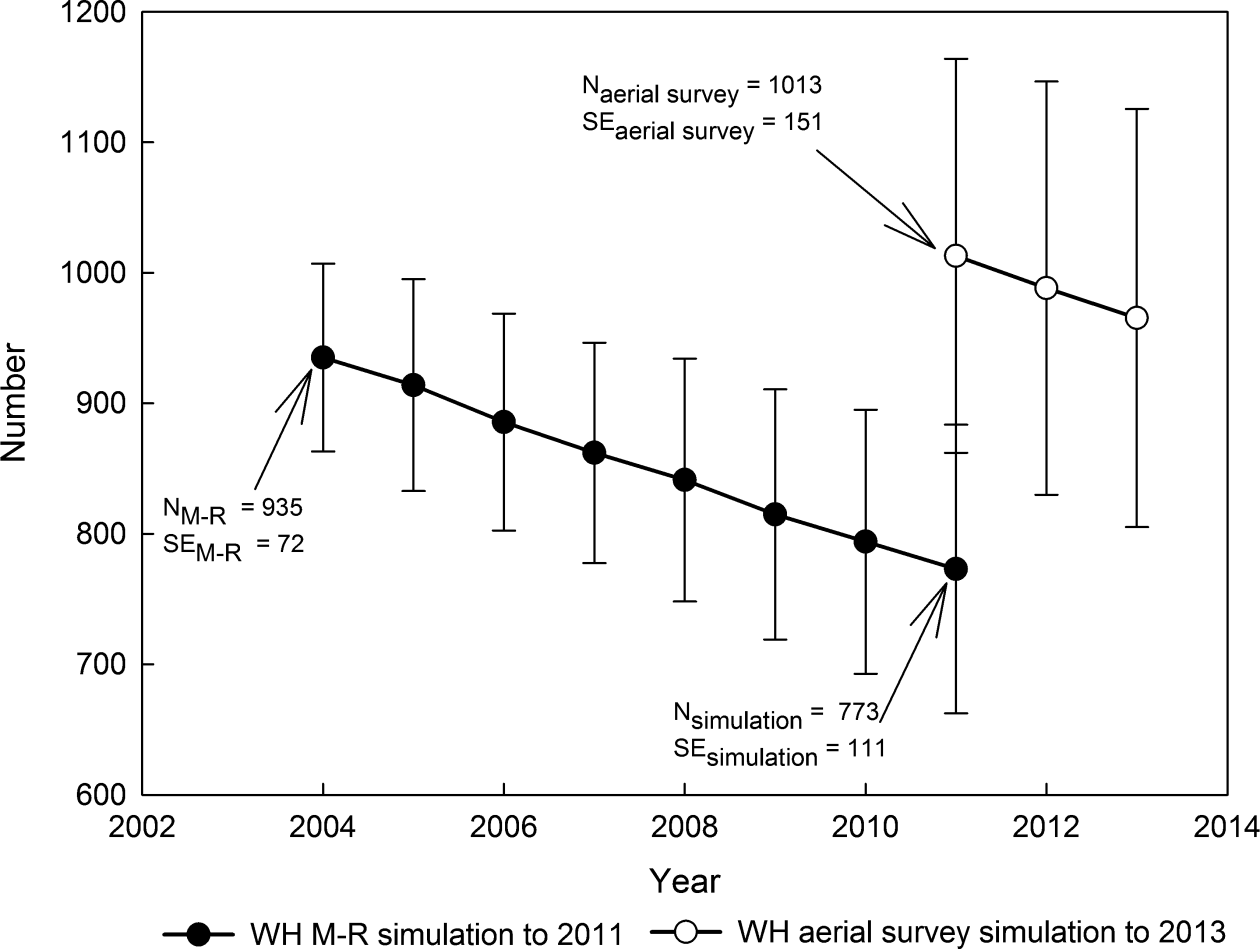
Comparison of the Western Hudson Bay (WH) subpopulation trajectory (RISKMAN simulation) from 2004 (mark–recapture estimate) to 2011 (aerial survey) estimate.

**Figure 7:**
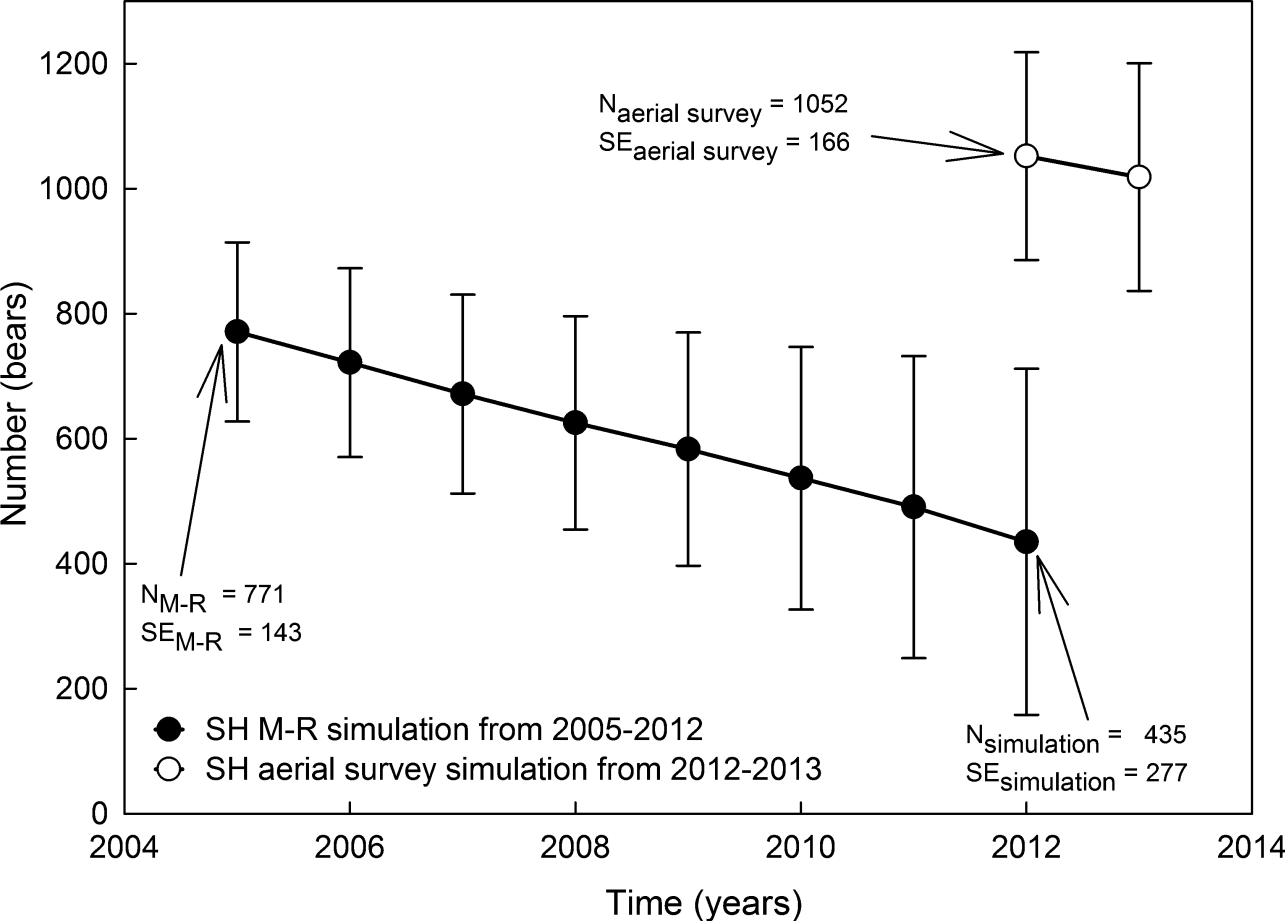
Comparison of the Southern Hudson Bay (SH) subpopulation trajectory (RISKMAN simulation) from 2005 (mark–recapture estimate) to 2012 (aerial survey) estimate.

A qualitative difference was identified for the comparison of simulation results (natural survival) to aerial survey for the FB (entire area sampled) than for SH and WH (partial area sampled) subpopulations (Table 10). Both the simulation and the aerial survey resulted in a numerical increase for FB; but the simulation resulted in a numerical decline for SH and WH, while the aerial survey indicated a numerical increase for the partially sampled subpopulations (Table 10). The difference between FB and SH/WH was also evident from the percent difference between the simulation and aerial estimates [100* (higher‐lower)/lower] values: FB (7.5%), SH (123%), WH (31%) (Table 10). However, the differences between the simulation and aerial survey estimates were not statistically significant for any the subpopulation comparisons (Table 10). A visual comparison of the M‐R‐based PVA trajectory and the aerial survey estimates for SH and WH (Stapleton et al. 2016; Obbard et al. 2013) are provided in Figures 6 and 7.

**Table 10:** Mark–recapture estimates (*N*), simulation estimates (Sim), and aerial survey estimates (Survey) of abundance are available for the Foxe Basin (FB), Southern Hudson Bay (SH), and Western Hudson Bay (WH) subpopulations. A two sample z‐test was used to compare the simulation results (natural survival) to aerial survey estimates for the FB (entire area sampled) and SH and WH (partial area sampled) subpopulations. While simulation results and aerial survey estimates appear numerically similar for FB (7.5% difference) and numerically different for SH (123% difference) and WH (31% difference), none of these differences were statistically significant (*P* > 0.05)

	Foxe Basin			
	*N*_0_ a	Sim_2010_ b	Survey_2010_ c	
Year	1994	2010	2010	
*N* (SE)	2200 (260)	2772.7 (1307.4)	2580 (278)	*P* ≤ 0.8854
	Southern Hudson Bay			
	*N*_0_ a	Sim_2012_ b	Survey_2012_ c	
Year	2005	2012	2012	
*N* (SE)	771 (143.3)	435.2 (276.8)	969 (202)	*P* ≤ 0.1193
	Western Hudson Bay			
	*N*_0_ a	Sim_2011_ b	Survey_2011_ c	
Year	2004	2011	2011	
*N* (SE)	935 (72)	773.0 (110.6)	1013 (151)	*P* ≤ 0.1198

a *N*_0_ represents the most recent estimate of abundance from mark–recapture studies.

b Sim_t_ represents the results of simulation from *N*_0_ to the year of the aerial survey.

c Survey_t_ represents the estimate from the most recent aerial survey; FB (Stapleton et al. 2016), SH (Obbard et al. 2013), WH (Stapleton et al. 2016).

**Figure 8:**
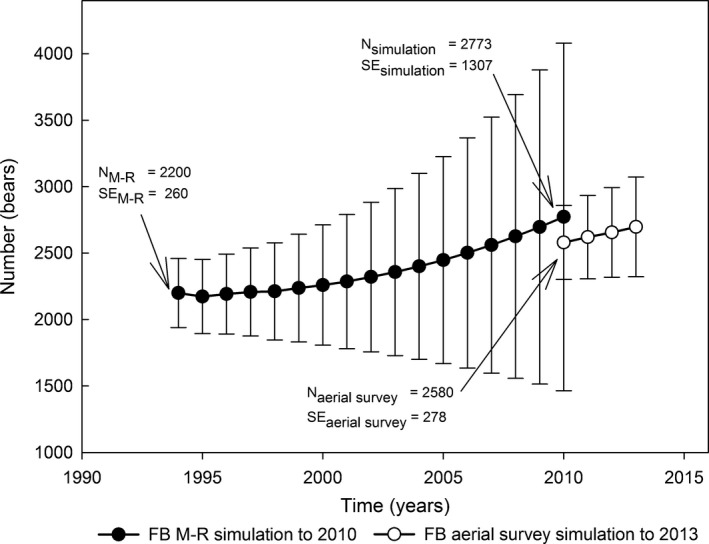
Comparison of the Foxe Basin (FB) subpopulation trajectory (RISKMAN simulation) from 1994 (tetracycline M‐R estimate) to 2010 (aerial survey) estimate using Baffin Bay (BB) birth and survival estimates (meta‐analysis) (S2: Table S2; S3: Table S1), and the mean annual FB harvest (S3: Tables S1a, S1b, and S1c).

We suggest that the lack of correspondence between PVA simulations results based on M‐R studies, aerial survey results, and TEK causes trend estimates for subpopulations that were partially M‐R sampled to be unreliable (Table 3). Given the correspondence between M‐R‐based PVA simulations, aerial surveys, and TEK (Tables 2b, 3, and 10; Figs. 6, 7, and 8), we suggest that TEK perspectives on polar bear subpopulation status, given historical harvest levels, provide both a consistency check and an accurate and reliable alternative status measure when scientific results are in doubt.

Stirling and Parkinson (2006) assert that seasonal subpopulations (BB, DS, FB, SH, WH) where polar bears seek onshore retreats during the open water season are also (in addition to WH) likely to decline. However, four of these subpopulations (DS, FB, SH, and WH) appear to have increased or remained at approximately historical levels since this study was published (FB: Stapleton et al. 2016; WH: Stapleton et al. 2016; DS: Peacock et al. 2013; SH: Obbard et al. 2013). Four of five seasonal polar bear subpopulations appear to have increased or remained constant, not declined as Stirling and Parkinson (2006) suggest. The BB subpopulation status is disputed (Tables 2b and 3; S1). It appears that the perception of decline stems from over‐reporting of the Greenland harvest (S1). In support of the TEK perspective, it seems unlikely that the BB subpopulation could have declined to less than half the number 15 years ago, without local hunters being aware of this decline (Table 2a; Fig. 4).

Concurrence with TEK and a low probability of decline (<0.5) suggests seven of the 13 subpopulations (DS, FB, GB, LS, MC, NW, and VM) are being harvested sustainably (Table 2b) and are not declining due to climate or any other effects. Of the remaining six subpopulations (BB, KB, NB, SB, SH, and WH), PVA simulations based on M‐R sampling indicate that these subpopulations are more likely in decline than stable or increasing, but only two of these subpopulations (BB and KB) employ M‐R estimates from subpopulations that were entirely sampled. Of the remaining four (NB, SB, SH, and WH), three subpopulations (NB, SB, and WH) have unharvested subpopulation growth rates that identify as nonviable (*λ*_H=0_ < 1.00), while the other one (SH) has an unharvested subpopulation growth rate estimated at less than 0.2% per year (Table 4).

Cluster analysis identifies one group of subpopulations (BB, KB, NB, SB, SH, WH) that had nonrandom/nonuniform capture sampling, or ambiguous harvest data, or was a source‐sink (not closed) subpopulation (S1), and that also had a high (> 0.5) probability of decline at current harvest levels; and a second group of subpopulations (DS, FB, GB, LS, MC, NW, VM) that were sampled throughout their seasonal range, had unambiguous harvest data, and were demographically closed subpopulations and had a low (<0.5) probability of decline. Within clusters, there was no relationship between productivity and probability of decline (Fig. 5). This suggests a qualitative difference between groups is methodological rather than ecological. We suggest that the difference is due to under‐estimation of subpopulation numbers and survival rates for NB, SB, SH, and WH; over‐estimation of Greenland harvest numbers for BB; and inappropriate application of a closed M‐R model to a subpopulation that could only persist with immigration from adjacent subpopulations (KB is nonviable with current and historical harvest rates) and apparently also has low productivity. Taylor et al. (2009) found the habitat in KB favorable for polar bears and cautioned that the KB abundance and survival estimates may have been affected (under‐estimated) by the source‐sink dynamics.

The expected number of individuals in KB at 2013 is zero (Table 2a) which is in agreement with TEK that KB has been subject to chronic long‐term overharvest and would not persist if it did not receive immigrants from adjacent subpopulations (Taylor et al. 2009). TEK and recent survey observations (Dyck, pers.com) confirm that polar bears are currently present in KB. The harvest rate for KB may have changed due to Greenland quotas implemented in January 2006 (Nunavut Wildlife Research Section, 2007) and climate warming‐related difficulties for Greenland hunters to reach KB from Thule. TEK for the remaining five “declining” subpopulations (Table 2b) indicates that they are stable or increasing. Except for speculation about eventual climate change effects (e.g., Stirling and Parkinson 2006; Amstrup et al. 2008; Stirling and Derocher 2012), the scientific perspective that BB is declining is based solely on PVA simulations that show that the joint Greenland/Nunavut harvest could not be sustained by the subpopulation (Taylor et al. 2005; Table 2b). In open water season, the BB subpopulation summered onshore on Baffin and Bylott Islands in the late 1990s (Taylor et al. 2001a). However, most of the bears harvested from this subpopulation are taken in the spring when the bears are on the sea ice (Lee and Taylor 1994). The Greenland harvest from BB was estimated from an unevaluated voluntary reporting system (Born 2007). It appears that the portion of the kill reported for west Greenland and assigned to BB subpopulation was over‐reported (S1). Over‐reporting in Greenland is possible because of the tradition of dividing polar bear skins among all the hunters that participate in the hunt (Born 2007), or possibly over‐reporting occurred in anticipation of a Greenland polar bear quota system. The M‐R estimates of the BB subpopulation numbers and productivity may have been under‐estimated; however, unlike the SH and WH summer retreat M‐R studies (Obbard et al. 2007; Regehr et al. 2007a,b), the entire subpopulation summer retreat area was sampled (Taylor et al. 2005). Until there is independent confirmation, the status of the BB subpopulation is best regarded as disputed, but our prediction based on TEK and the accuracy of other subpopulation estimates where sampling was representative is that subpopulation numbers will have remained about the same.

Some studies (Stirling and Parkinson 2006; Stirling and Derocher 2012) suggest that TEK is unduly optimistic because aboriginal people have become confused about the true trends of subpopulations in their area by seeing increased numbers of hungry bears congregating near their communities, then falsely generalizing a positive subpopulation trend from these local concentration sightings. The 2005 PBSG passed the only nonunanimous resolution in its history stating that “(The IUCN Polar Bear Specialist Group) recommends that polar bear harvests can be increased on the basis of local and traditional knowledge only if supported by scientifically collected information” (Aars et al. 2006:57). In other words, using TEK is accepted, but only if it agrees with scientific results. The converse (e.g., scientific results only accepted if TEK concurs) was not proposed. Thus, “precautionary” to the 2005 PBSG meant using scientific results if science and TEK differ, but accepting TEK only if it supports the scientific perspective.

We suggest that both management of natural resources action and the collective understanding of natural phenomena would be enhanced by simply comparing scientific and Type 1 and Type 2 (Usher 2000) traditional knowledge. When Science and TEK differ, both scientific and TEK perspectives should be critically reviewed to understand the reason for the disagreement. The review should be collegial and collaborative rather than defensive and acrimonious in order to remain faithful to the principles of both knowledge systems. This will require the systematic allocation of resources to both scientific research and TEK documentation. The biggest challenge will be to identify a program of study that can resolve differences between knowledge systems. TEK holders that are typically the primary users of natural resources and should be involved in scientific research, as much as possible. Scientific researchers should also support and participate in relevant TEK studies. With respect to the cultural and social value systems that underlie both scientific and traditional knowledge, we suggest that information about the environment and the use of the environment (Usher's (2000) Type 1 and Type 2 TEK) that is provided by both systems for management purposes should be as value free and culturally independent as possible. Considerations of social and cultural values and the knowledge systems employed (Usher's (2000) Type 3 and Type 4 TEK) are relevant to management decisions, but should be considered separately and in the context of the governance system. Reconciliation of social and cultural differences relevant to management options is a political issue and requires a political process (e.g., co‐management boards). However, conservation decisions should be mainly guided by objective, properly qualified, and value‐free information; regardless of its source. Viewed this way, application of the precautionary principle when information is incomplete or suspect would function as an interim fair protocol that considers all available information and balances conservation concerns with impacts to resource users (Government of Canada, 2003).

### Examples of TEK as a successful indicator of trend for polar bears

There are a number of previous incidences of TEK/science conflict in polar bear management where subsequent studies showed that TEK was correct and scientific results were incorrect (S6). There have also been instances where TEK proved to be conservative rather than exploitive prior to the availability of scientific information. In Baffin Bay, the 1993‐1997 subpopulation study (Taylor et al. 2005) showed the 1974‐1979 M‐R study estimates were mathematically impossible because the known harvest would have extinguished the subpopulation (unpublished NWT file report, 1980; Davis 1999). The 1974–1979 study estimated 350–600 bears for the whole subpopulation (unpublished NWT file report, 1980) which led to a quota reduction of 45/year and annual compensation payments of $1000,00 per bear until 1996 (Davis 1999). Baffin Bay Inuit disagreed that polar bears were so few in number. In 1993, a polar bear came into Clyde River, Nunavut, and became trapped in the school yard during community consultations on polar bear quotas (M. Taylor, pers. comm. 1986–2008). The bear was chased out of the community, and the Clyde River and Qikitarjuaq quotas were increased. The 1997 BB study (Taylor et al. 2005) was conducted jointly with local Inuit and estimated the subpopulation to number 2074 in 1997. The problem with the initial study was the failure to sample throughout the subpopulation area. The 1971–1976 capture crews were working in spring and could not search and capture past the floe edge because the pack ice was too unstable to immobilize polar bears safely. Thus, only a portion of the subpopulation was sampled (Schweinsburg et al. 1981; Taylor et al. 2005). We are aware of four instances (FB, KB, MC, and VM) where TEK identified a subpopulation decline before corroborating scientific information was available to confirm it (Taylor et al. 2002, 2006a,b, 2008a), and four instances where TEK identified stable or increasing subpopulations (DS, GB, LS, and NW) before a study confirmed it (Taylor et al. 2008b, 2009; Peacock et al. 2011, 2013; M. Taylor, pers. comm. 1986–2008; M. Dowsley, pers. comm. 2003–2012). Short descriptions of these eight instances are provided in S6.

We observed only one conflict (BB) between PVA simulations and TEK when the M‐R‐based demographic estimates were based on total area sampling (Table 3). In all cases, involving perceptions of trend in polar bear numbers that we are familiar with, when science and TEK did not agree, and subsequent research became available; the new results indicated that the TEK perspective on trend was correct (S6).

Scientific perceptions that polar bears are currently declining due to climate warming is based on observed declines in body condition (Stirling et al. 1999, 2008; Obbard et al. 2006; Rode et al. 2007, 2012), survival and subpopulation estimates that are suspect because of M‐R sampling problems (Table 6), and untested nutritional–ecological models (Molnár et al. 2008, 2010, 2011). TEK perspectives that polar bear subpopulations remain at or above historical levels appear to be supported by both PVA analysis where sampling is subpopulation wide and by recent aerial surveys of subpopulations where M‐R estimates were based on partial sampling of the subpopulation area (Obbard et al. 2013; Peacock et al. 2013; Stapleton et al. 2014, 2016; Table 2b). Inconsistencies between our status determinations and those prepared by various polar bear specialists groups and others appear to be due to an inconsistent use of published subpopulation demographic estimates and use of subjective status categories (e.g., “data deficient” for subpopulations where there are data) that we cannot explain. Harvested subpopulations that either do not have sufficient productivity to sustain themselves without harvest (e.g., NB, SB, WH) or would decline with occasional removals (e.g., SH) are sometimes identified as stable (e.g., NB and SH; Obbard et al. 2010; Vongraven and Richardson 2011), and subpopulations that are most probably increasing are identified as declining or data deficient (e.g., DS (decline) and FB (data deficient); Obbard et al. 2010). Determinations in other recent status reports contain a mixture of old subpopulation estimates and partially projected estimates with no explanation of why projection estimates were used for some subpopulations but not for others (Committee on the Status of Endangered Wildlife in Canada 2008; Polar Bear Technical Committee (PBTC) 2009; Obbard et al. 2010; Vongraven and Richardson 2011). Scientific information on declines in body condition associated with declines in sea ice was also based on a geographic subsampling of subpopulations; however, the body condition analysis assumptions did not require that every individual in the subpopulation was available for sampling, only that the individuals sampled were representative of the entire subpopulation (Rode et al. 2012). The scope of this review did not include a comparison of TEK versus scientific information on trends in polar bear body condition; but we would expect general agreement between both perspectives because the sampling for the scientific perspective seems appropriate.

### Climate, sea ice change, and population viability analysis

A demographic approach to population viability typically assumes that the mean and variance of survival and recruitment rates remain constant for the simulation period. When demographic parameters change progressively as a result of density effects or progressive environmental effects, demographic effects can be modeled as functions of the controlling variables when both the functional relationships are known and the future values of the controlling variables can be estimated (e.g., sea ice decline as per Molnár et al. 2011). Amstrup et al. (2007, 2008) suggested that ~67% of all polar bears would lost by 2050 if CO_2_ emissions were not curtailed due to sea ice loss. Stirling and Derocher (2012) review the evidence for climate warming and sea ice reduction effects on polar bear subpopulation numbers and vital rates. However, we found the evidence for sea ice mediated declines in subpopulation numbers and survival rates to be restricted to M‐R studies where only a portion of the subpopulation seasonal range had been sampled. Evidence of reduced body condition and reduced recruitment rates associated with sea ice decline in the BB, DS, SB, SH, and WH subpopulations (Stirling et al. 1999, 2008; Obbard et al. 2006; Rode et al. 2007, 2012) was unambiguous for SB, SH, and WH; however, evidence from BB was compromised because the body condition data that were compared were taken in different parts of the subpopulation area. Evidence for body condition decline as a function of sea ice reduction is ambiguous for DS because subpopulation density was increasing throughout the same period that sea ice was declining (Rode et al. 2012). Rode et al. (2014) found that adult females in the Chukchi Sea (CS) increased in body mass, had larger litters and heavier yearlings during a period of sea ice decline. RISKMAN has the capacity to model density effects, but the mechanism for density effects for polar bears has not been described or quantified for any subpopulation (Taylor 1994). We did not find sufficient development of relationships between sea ice and demographic rates, or density effects and demographic rates to incorporate these dimensions into our analyses. For further discussion on climate and sea ice effects on polar bears, refer to S7.

### Management considerations

We do not advocate polar bear management based on indefinite extrapolation of historical data. In addition to changing environmental conditions, the uncertainty associated with stochastic simulations increases with time. Monte Carlo estimates of geometric subpopulation growth rate are compromised (biased) when simulations must be truncated at zero. Large variances associated with subpopulation estimates (either simulation estimates or survey estimates) can result in Monte Carlo simulation truncations due to random variants ≤0 (S5). With few exceptions, the demographic data for reliable PVA for Canadian subpopulations are almost expired. There is a need to monitor all harvested subpopulations and periodically update the demographic information in order to estimate demographic performance, harvest sustainability, and subpopulation status. Surveys that provide only subpopulation estimates (e.g., aerial surveys) or do not provide the full complement of age‐structured survival and recruitment estimates (e.g., DNA M‐R) may not provide sufficient data to estimate current trends or project future subpopulation numbers. Environmental conditions change and adjustments to management are necessary for long‐term sustainability, especially when subpopulations are harvested near maximum sustainable rates. We advocate a more inclusive approach to polar bear management that would employ recent and reliable TEK to identify any lack of correspondence between TEK and scientific knowledge. Resolving these areas of disagreement could only enhance both science and TEK and improve management practices, but would obviously require simultaneous scientific research and enhanced TEK collection to function effectively. With respect to scientific monitoring of polar bear subpopulation trends, we recommend estimation of the full demographic compliment required to achieve accurate estimates of subpopulation status and guide harvest quotas.

### Future scientific research

As discussed above, our PVA model software (RISKMAN) does not have a way of incorporating a progressive decline in survival or recruitment as might be expected from a continuing decline in environmental conditions due to climate change, industrial development, tourism, or other factors that could result in negative demographic effects on the polar bear subpopulations. We chose a simulation period of 20 years to estimate the likelihood of a decline to allow for a demographic (standing age distribution) response to sex‐selective harvesting (Taylor et al. 2008c), but we do not suggest that conditions are likely to remain constant for that interval of time. Another limitation to current PVA simulation models is the lack of parameter co‐variance estimates and estimates of how total variance is partitioned into environmental and parameter components for survival and recruitment rate estimates (White 2000). We investigated the effect of our variance partitioning convention (parameter variance = 75%, environmental variance = 25%, co‐variance = 0) by exploring a range of partitioning assumptions for each subpopulation (Table 8). The effects of variance partitioning on PVA simulation results appeared to be minor, but may become more important if the environment becomes less stable (more variable) as predicted by climate models. We also examined the effect of co‐variance by comparing the change probability of decline for a set of simulations using parameter variance = 75%/environmental variance = 25% and covariance set to either *R* = 0 (independent) or *R* = 1 (100% correlated) (Table 9). No qualitative changes on PVA simulation results were found except for SH (decline) and KB (decline) when *R* = 1. The SH subpopulation was unable to sustain even occasional removals, and the KB demographic estimates were exceptionally uncertain due to small sample size and the source‐sink dynamics of this subpopulation. More accurate harvest reporting from shared subpopulations (especially those shared by Greenland and Quebec) and M‐R sampling of entire subpopulation areas would improve the accuracy and reliability of PVA simulations.

## Conflict of Interest

None declared.

## Supporting information


**S1: Figure S1.** Baffin Bay (BB) subpopulation trajectories from 1997 to 2013 comparing the effect of different BB survival rates (Taylor et al., 2005 [Natural]; Peacock et al., 2011 [Natural and Total]).

**S5: Figure S1.** The potential effect of truncated runs on subpopulation abundance was estimated from a series of RISKMAN simulations using increasing initial subpopulation variance (CV) for the Viscount Melville Sound (VM) subpopulation.

**S5: Figure S2.** The potential effect of truncated runs on geometric subpopulation growth rate estimated from a set of 100 Monte Carlo iterations for the Viscount Melville Sound (VM) subpopulation for 20 year period under a harvest moratorium.

**S7: Figure S1.** HadCRUT4 annual global temperature for the 1980–2013 period.

**S7: Figure S2.** REMSS annual Arctic temperature for the 1980–2013 period.

**S7: Figure S3.** NOAA‐NODC global ocean heat content (0–700 m) for the 1980–2013 period.

**S7: Figure S4.** NSIDC annual Arctic sea ice extent for the 1980–2013 period.

**S7: Figure S5.** Observed monthly sea ice extent (NSIDC) for the Arctic from January 1980 to December 2013.

**S7: Figure S6.** Annual global temperature (HadCRUT4) and monthly sea ice extent (NSIDC) for the Arctic during the January 1980 to December 2013 period.

**S7: Figure S7.** Global mean temperature near−term projections relative to 1986−2005 (From: Kirtmen et al., (2013) Figure 11.25).

**S7: Figure S8.** Arctic September sea ice extent (×106 km^2^) from observations and 13 IPCC AR4 climate models, together with the multi‐model ensemble mean (solid black line) and standard deviation (From Stroeve et al., 2007, Fig. 1).

**S7: Figure S9.** Arctic March sea ice extent (×106 km^2^) from observations and 18 IPCC AR4 climate models together with the multi‐model ensemble mean and standard deviation (From Stroeve et al., 2007, Fig. 2).

**Supplementary 1.** Ecological summaries of Canadian polar bear subpopulations
**S1: Table S1.** Baffin Bay (BB) mortality rates were applied to this RISKMAN stable‐age distribution as a consistency check between the Peacock et al., 2011 estimated survival rates and the BB reported harvest.
**S1: Table S2.** Baffin Bay (BB) mortality rates (natural, total, and harvest) based on the natural and total survival rates reported in Peacock et al., 2011.
**S1: Table S3.** Baffin Bay (BB) marked/unmarked bears by jurisdiction (Nunavut and Greenland) for the 1998–2009 period.
**S1: Table S4.** A comparison of Nunavut versus Greenland Baffin Bay recoveries for the 1998–2000, 1998–2001, 1998–2002, and 2003–2009 time bins
**Supplementary 2.** Canadian Polar Bear Subpopulation Survival Rates.
**S2: Table S1.** Mean (standard error [SE] in parentheses) of total (i.e., harvested) annual survival rates for age and sex classes of subpopulations of Canadian polar bears.
**S2: Table S2.** Mean (standard error [SE] in parentheses) of natural (i.e., unharvested) annual survival rates for age and sex classes of subpopulations of Canadian polar bears.
**Supplementary 3.** Canadian Polar Bear Recruitment Rates.
**S3: Table S1.** Estimated means (and standard errors [SE] in parentheses) of post‐den emergence litter size and age‐specific probabilities of litter production (LPR) for lone females or females with dispersing (2‐year‐old) cubs.
**Supplementary 4.** Total Human‐Caused Mortality Rates for Canadian Polar Bear Subpopulations.
**S4: Table S1a.** Total anthropogenic (harvest, defense, accidental, and illegal) mortality rates (Kill) and the proportion that were females (Prop F) for each Canadian subpopulation, summarized by harvest season for the 1993/1994 to 1999/2000 interval (York, 2012; PBTC, 2013).
**S4: Table S1b.** Total anthropogenic (harvest, defense, accidental, and illegal) mortality rates (Kill) and the proportion that were females (Prop F) for each Canadian subpopulation, summarized by harvest season for the 2000/2001 to 2006/2007 interval (York, 2012; PBTC, 2013).
**S4: Table S1c.** Total anthropogenic (harvest, defense, accidental, and illegal) mortality rates (Kill) and the proportion that were females (Prop F) for each Canadian subpopulation, summarized by harvest season for the 2007/2008 to the 2011/2012 interval (York, 2012; PBTC, 2013).
**Supplementary 5.** Effect of Truncated Iterations on Monte Carlo Estimates of Subpopulation Growth Rate (λ).
**S5: Table S1.** The effect of including/excluding truncated runs in the calculations of geometric mean subpopulation growth rates (λ).
**Supplementary 6.** Eight Instances Where TEK Identified a Polar Bear Subpopulation Trend or Biological Feature before Science Could Identify or Confirm It.
**Supplementary 7.** Evaluation of Global Temperature, Arctic temperature, Global Ocean Heat Content (0–700 m), Arctic Sea Ice Extent Trends, and the Effects of Climate Change on Canadian Polar Bears.
**S7: Table S1.** HadCRUT4 global temperature, REMSS Arctic temperature, NSIDC sea ice extent, and NOAA‐NODC ocean heat content data for the 1980–1996 period.
**S7: Table S2.** HADCRUT4 global temperature, REMSS Arctic temperature, NSIDC sea ice extent, and NOAA‐NODC ocean heat content data for the 1997–2013 period.
**S7: Table S3.** Examining Ho: slope = 0 for world ocean heat content, global atmospheric temperature, Arctic (60–82.5 N) atmospheric temperature, and annual Arctic sea ice extent vs. time.
**S7: Table S4.** Examining Ho: slope = 0 for monthly Arctic sea ice extent vs. time for the January 1980 to December 2013 period.
**S7: Table S5.** Examining Ho: slope = 0 for monthly Arctic sea ice extent vs. time for the period at which the relationship between the two variables was no longer significant.
**S7: Table S6.** Examining Ho: slope = 0 for monthly Arctic sea ice extent vs. time for the period prior to the breakpoint, at which the relationship between the two variables was no longer significant.
**S7: Table S7.** Examining: correlations for annual global temperatures vs. monthly Arctic sea ice extent for the 1980–2013 period.
**S7: Table S8.** Examining correlations for annual global temperatures vs. monthly Arctic sea ice extent for the period at which the relationship between the two variables was no longer significant.
**S7: Table S9.** Comparing Arctic atmospheric temperature means for the time periods identified by a breakpoint regression of Arctic temperatures since 1980.
