# Supplementary figures and images for: Demographic and traditional knowledge perspectives on the current status of Canadian polar bear subpopulations
Source: Ecol Evol. 2016 Mar 23;6(9):2897–924. doi: 10.1002/ece3.2030 (PMC4804000; doi:10.1002/ece3.2030)

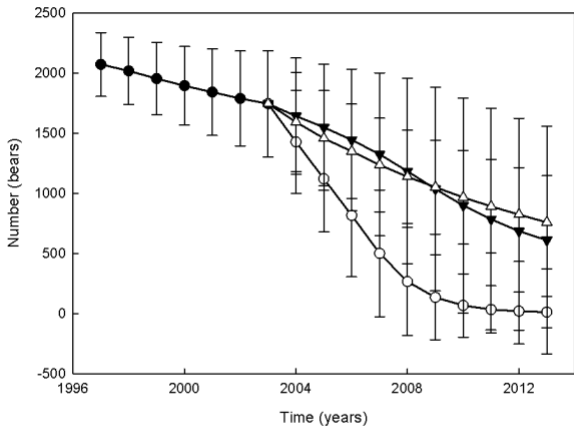

- 1997-2003 Taylor et al., 2005 NAT
- 2003-2013 Peacock et al., 2012 NAT
- ▼ 2003-2013 Taylor et al., 2005 NAT
- △ 2003-2013 Peacock et al., 2012 TOT

Supplement: Supplementary file 1 — S1: Figure S1. Baffin Bay (BB) subpopulation trajectories from 1997 to 2013 comparing the effect of different BB survival rates (Taylor et al., 2005 [Natural]; Peacock et al., 2011 [Natural and Total]). [file ECE3-6-2897-s001.pdf]

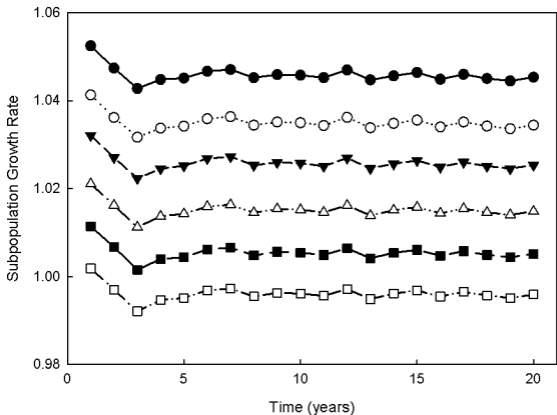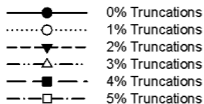

Supplement: Supplementary file 3 — S5: Figure S2. The potential effect of truncated runs on geometric subpopulation growth rate estimated from a set of 100 Monte Carlo iterations for the Viscount Melville Sound (VM) subpopulation for 20 year period under a harvest moratorium. [file ECE3-6-2897-s003.pdf]

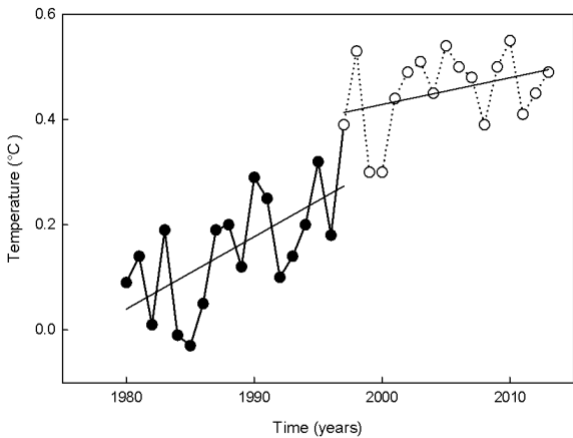

Supplement: Supplementary file 4 — S7: Figure S1. HadCRUT4 annual global temperature for the 1980–2013 period. [file ECE3-6-2897-s004.pdf]

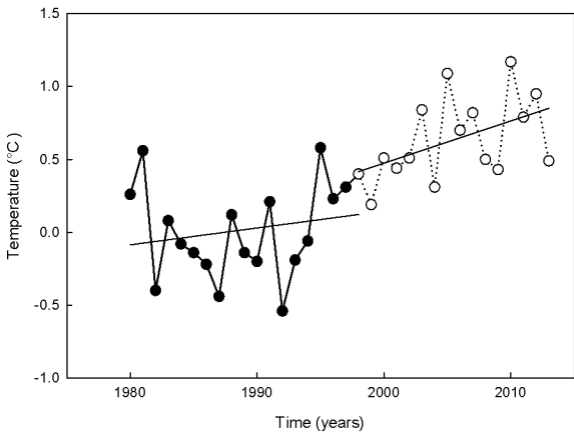

Supplement: Supplementary file 5 — S7: Figure S2. REMSS annual Arctic temperature for the 1980–2013 period. [file ECE3-6-2897-s005.pdf]

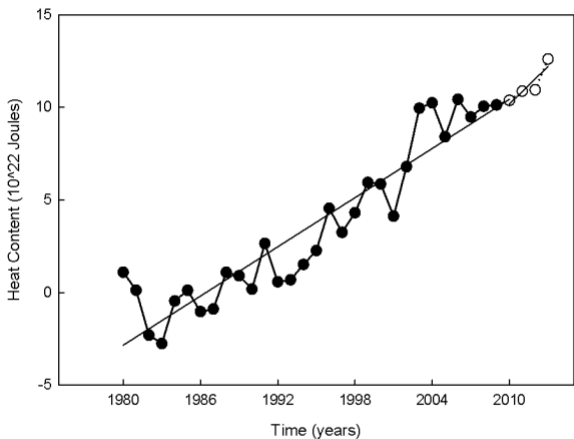

Supplement: Supplementary file 6 — S7: Figure S3. NOAA‐NODC global ocean heat content (0–700 m) for the 1980–2013 period. [file ECE3-6-2897-s006.pdf]

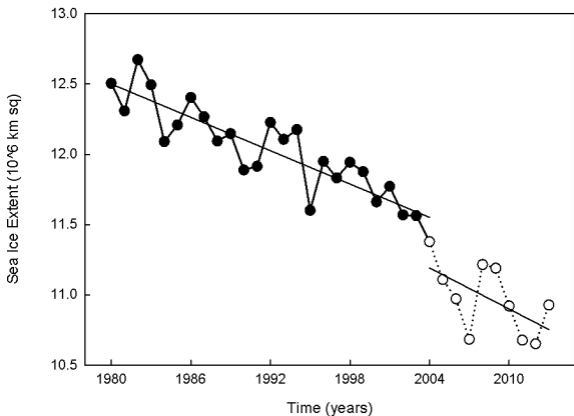

Supplement: Supplementary file 7 — S7: Figure S4. NSIDC annual Arctic sea ice extent for the 1980–2013 period. [file ECE3-6-2897-s007.pdf]

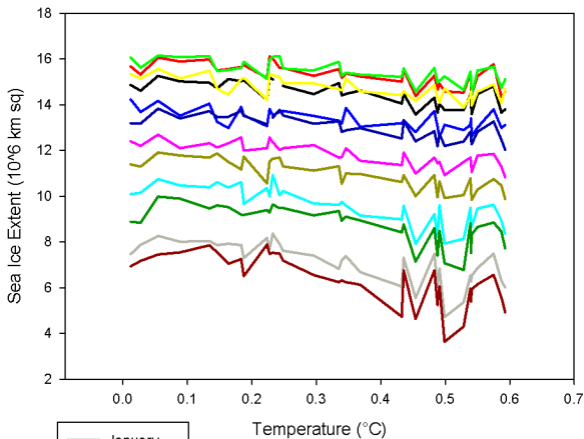

- January
- February
- March
- April
- May
- June
- July
- August
- September
- October
- November
- December

Supplement: Supplementary file 9 — S7: Figure S6. Annual global temperature (HadCRUT4) and monthly sea ice extent (NSIDC) for the Arctic during the January 1980 to December 2013 period. [file ECE3-6-2897-s009.pdf]

# Global mean temperature near-term projections relative to 1986–2005

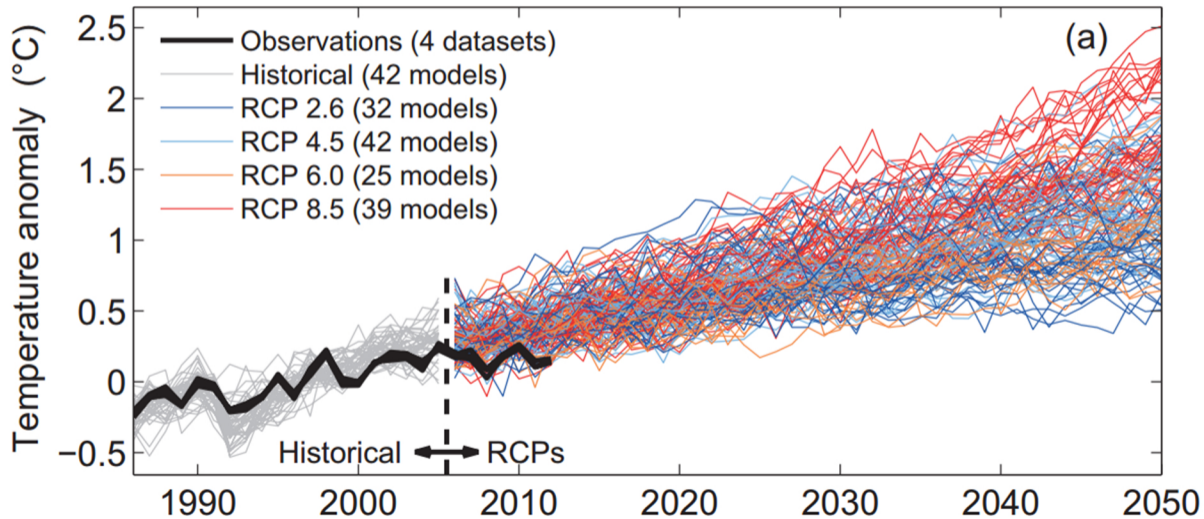

Supplement: Supplementary file 10 — S7: Figure S7. Global mean temperature near−term projections relative to 1986−2005 (From: Kirtmen et al., (2013) Figure 11.25). [file ECE3-6-2897-s010.pdf]

Arctic  
September Sea Ice Extent: Observations and Model Runs

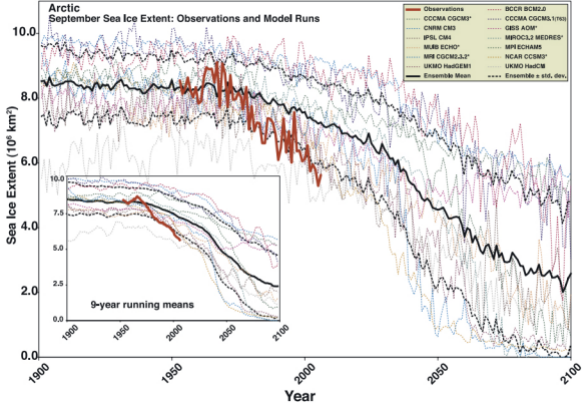

Supplement: Supplementary file 11 — S7: Figure S8. Arctic September sea ice extent (×106 km2) from observations and 13 IPCC AR4 climate models, together with the multi‐model ensemble mean (solid black line) and standard deviation (From Stroeve et al., 2007, Fig. 1). [file ECE3-6-2897-s011.pdf]

# Arctic March Sea Ice Extent: Observations and Model Runs

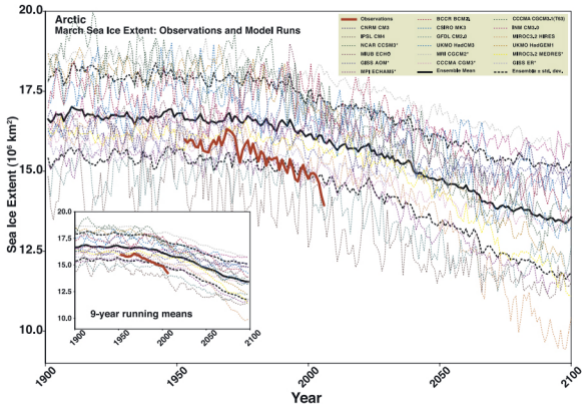

Supplement: Supplementary file 12 — S7: Figure S9. Arctic March sea ice extent (×106 km2) from observations and 18 IPCC AR4 climate models together with the multi‐model ensemble mean and standard deviation (From Stroeve et al., 2007, Fig. 2). [file ECE3-6-2897-s012.pdf]
