# Supplementary material for: Demographic and traditional knowledge perspectives on the current status of Canadian polar bear subpopulations
Source: Ecol Evol. 2016 Mar 23;6(9):2897–924. doi: 10.1002/ece3.2030 (PMC4804000; doi:10.1002/ece3.2030)
Supplement: Supplementary file 2 — S5: Figure S1. The potential effect of truncated runs on subpopulation abundance was estimated from a series of RISKMAN simulations using increasing initial subpopulation variance (CV) for the Viscount Melville Sound (VM) subpopulation. [file ECE3-6-2897-s002.pdf]

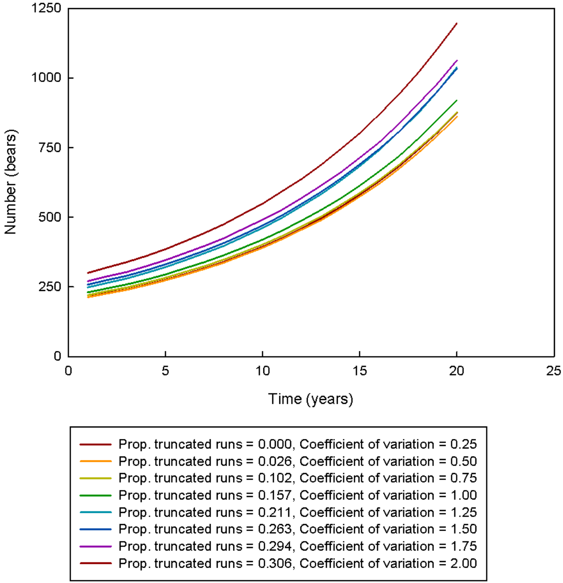
1250
1000
750
500
250
0
Number (bears)
0
5
10
15
20
25
Time (years)
Prop. truncated runs = 0.000, Coefficient of variation = 0.25
Prop. truncated runs = 0.026, Coefficient of variation = 0.50
Prop. truncated runs = 0.102, Coefficient of variation = 0.75
Prop. truncated runs = 0.157, Coefficient of variation = 1.00
Prop. truncated runs = 0.211, Coefficient of variation = 1.25
Prop. truncated runs = 0.263, Coefficient of variation = 1.50
Prop. truncated runs = 0.294, Coefficient of variation = 1.75
Prop. truncated runs = 0.306, Coefficient of variation = 2.00
