# Supplementary material for: Demographic and traditional knowledge perspectives on the current status of Canadian polar bear subpopulations
Source: Ecol Evol. 2016 Mar 23;6(9):2897–924. doi: 10.1002/ece3.2030 (PMC4804000; doi:10.1002/ece3.2030)
Supplement: Supplementary file 8 — S7: Figure S5. Observed monthly sea ice extent (NSIDC) for the Arctic from January 1980 to December 2013. [file ECE3-6-2897-s008.pdf]

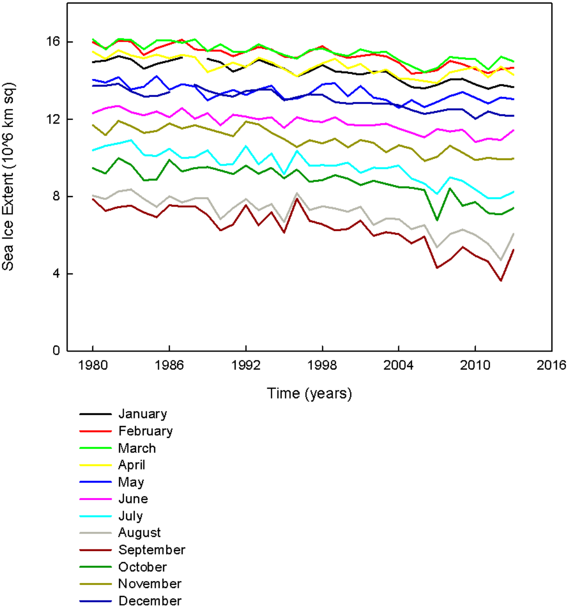

Sea Ice Extent (10^6 km sq)
0
4
8
12
16
1980
1986
1992
1998
2004
2010
2016
Time (years)
January
February
March
April
May
June
July
August
September
October
November
December
