# Supplementary material for: Demographic and traditional knowledge perspectives on the current status of Canadian polar bear subpopulations
Source: Ecol Evol. 2016 Mar 23;6(9):2897–924. doi: 10.1002/ece3.2030 (PMC4804000; doi:10.1002/ece3.2030)
Supplement: Supplementary file 13 — Supplementary 1. Ecological summaries of Canadian polar bear subpopulations S1: Table S1. Baffin Bay (BB) mortality rates were applied to this RISKMAN stable‐age distribution as a consistency check between the Peacock et al., 2011 estimated survival rates and the BB reported harvest. S1: Table S2. Baffin Bay (BB) mortality rates (natural, total, and harvest) based on the natural and total survival rates reported in Peacock et al., 2011. S1: Table S3. Baffin Bay (BB) marked/unmarked bears by jurisdiction (Nunavut and Greenland) for the 1998–2009 period. S1: Table S4. A comparison of Nunavut versus Greenland Baffin Bay recoveries for the 1998–2000, 1998–2001, 1998–2002, and 2003–2009 time bins Supplementary 2. Canadian Polar Bear Subpopulation Survival Rates. S2: Table S1. Mean (standard error [SE] in parentheses) of total (i.e., harvested) annual survival rates for age and sex classes of subpopulations of Canadian polar bears. S2: Table S2. Mean (standard error [SE] in parentheses) of natural (i.e., unharvested) annual survival rates for age and sex classes of subpopulations of Canadian polar bears. Supplementary 3. Canadian Polar Bear Recruitment Rates. S3: Table S1. Estimated means (and standard errors [SE] in parentheses) of post‐den emergence litter size and age‐specific probabilities of litter production (LPR) for lone females or females with dispersing (2‐year‐old) cubs. Supplementary 4. Total Human‐Caused Mortality Rates for Canadian Polar Bear Subpopulations. S4: Table S1a. Total anthropogenic (harvest, defense, accidental, and illegal) mortality rates (Kill) and the proportion that were females (Prop F) for each Canadian subpopulation, summarized by harvest season for the 1993/1994 to 1999/2000 interval (York, 2012; PBTC, 2013). S4: Table S1b. Total anthropogenic (harvest, defense, accidental, and illegal) mortality rates (Kill) and the proportion that were females (Prop F) for each Canadian subpopulation, summarized by harvest season for the 2000/2001 to 20 [file ECE3-6-2897-s013.docx]

Demographic and Traditional Knowledge Perspectives on the Current Status of Canadian Polar Bear Subpopulations

**Supplementary 1.** Ecological summaries of Canadian polar bear subpopulations derived from various status reports (COSEWIC, 2008; Taylor & Dowsley, 2008; Obbard et al., 2010) were updated to include information from recent studies.

*Baffin Bay (BB)*

Based on movements of adult females equipped with satellite radio-collars and recaptures of tagged animals, the area in which the BB subpopulation occurs is bounded by the North Water Polynya to the north, Greenland to the east and Baffin Island to the west (Taylor & Lee, 1995; Taylor et al., 2001a). A relatively distinct southern boundary at Cape Dyer (Baffin Island) is evident from the movements of tagged bears (Stirling et al., 1980) and recent movement data from polar bears monitored by satellite telemetry (Taylor et al., 2001a). A study of micro-satellite variation did not reveal any genetic differences between polar bears in BB and Kane Basin (KB), although bears of BB differed significantly from those of Davis Strait (DS) and Lancaster Sound (LS) (COSEWIC, 2008; Paetkau et al., 1999). An initial BB subpopulation estimate of 300–600 bears was made by the Government of the Northwest Territories from mark-recapture (M-R) data collected in spring of 1984–1989. However, both telemetry and hunter-kill returns have shown that an unknown proportion of the subpopulation was typically offshore during the spring and, therefore, unavailable for capture. A second study (1993–1997) was carried out annually during the months of September and October, when all polar bears were on shore in summer retreat areas on Bylot and Baffin islands (Taylor et al., 2005). Taylor et al. (2005) estimated the number of polar bears in BB (1998 estimate) at 2074 bears (SE = 266).

The BB subpopulation of polar bears is shared with Greenland, which until January 2006, did not limit the number of bears killed in a year. Based on M-R sampling and harvest recoveries of marked bears, Taylor et al. (2005) estimated the Greenland annual removal at 18–35 bears for the period 1993–1997. However, Born (2002) reported that the estimated the Greenland average annual catch of polar bears from BB was 73 bears per year over the period 1993–1998. Greenland also reported that the average kill by Greenland hunters in BB for the period 2002–2007 was 147 bears per year (range: 75–206 bears per year; COSEWIC, 2008; PBTC, 2008; Obbard et al., 2010). The current (2007-2012) combined five year average reported kill for BB is 167.4 (Table 2a).

The 2004 estimate of <1600 bears is based on subpopulation simulations that employ the pooled Canadian and Greenland harvest records since 1998 (PBTC, 2006, 2007; Obbard et al., 2010). Obviously, if the subpopulation was declining in 2004, and the number of removals was not reduced, there would be still fewer individuals in 2013. Greenland adopted a quota system effective 1 January 2006. However, the 100-bear West Greenland quota will likely include 75–85 bears taken per year in BB, with the remainder being taken from KB (5-25 annually) and DS (<5 annually). In response to community suggestions that polar bears increased in abundance in recent years, the Government of Nunavut increased its quota in BB from 64 to 105 bears in December of 2004. However, when the increased Greenland kill was reported in 2004, and in response to national and international pressure of over-hunting, a phased (10 per year) reduction of the Nunavut BB quota to 65 was initiated. The current 167.4 five-year average annual removal rate in 2012 (Table 2a; S4 Table S1c) is 21% less than the 2009 five-year average annual removal rate of 212 (Obbard et al., 2010). Greenland has thus far not reduced its West Greenland quota, or instituted a harvest program that validates which subpopulation West Greenland kills are assigned to. For many Greenland kills, the location of the kill is recorded as the location of the community that hunter lives in (Obbard et al., 2010).

Baffin Bay Inuit have reported higher or constant abundance of BB polar bears in recent years. TEK from three Baffin Bay communities (Pond Inlet, Clyde River and Qikiqtarjuaq) indicates that hunters and residents have been seeing more polar bears on the land and around communities in the past few years compared to 10–15 years ago (Dowsley, 2005). Significantly more people in the two northern communities experienced this increase compared to people in Qikiqtarjuaq (Dowsley, 2005). Bear encounters have increased, especially among Pond Inlet and Clyde River outpost camp residents, and safety concerns have grown for people on the land, as well as concerns about damaged property (Dowsley and Taylor, 2006a).

From the perspective of population viability analysis (PVA), the BB subpopulation is substantially over-harvested. The discrepancy between TEK and scientific data regarding the trajectory of the BB subpopulation of polar bears is a matter of concern. It has been suggested (Stirling and Parkinson, 2006) that local observations of increased abundance may be due to higher levels of bear activity in response to increased time spent on-shore by polar bears in response to climate warming in the region. Movements inland during summer have apparently increased in places in recent years, but numbers seen near communities have not increased (Dowsley, 2005). All three Baffin Bay communities have reported climate change impacts on the sea ice, such as less shore-fast ice, fewer icebergs and thinner ice, which some people (5/12 people who discussed the idea) thought might contribute to changes in polar bear distribution (Dowsley, 2005; Dowsley and Taylor, 2006a). However, no Inuit respondents expressed confusion about the reason for increased sightings of polar bears or felt that densities around communities were greater than densities elsewhere. The consensus among Baffin Bay Inuit is that they are seeing more bears because there are more bears (Dowsley, 2005; Dowsley & Taylor, 2006a), in spite of simulation results suggesting that the subpopulation should have been reduced to less than half of its number in 1997.

Peacock et al. (2012) use the harvest recoveries from the previous M-R studies to estimate natural and total survival rates for various time intervals from 1979 to 2009. Although all estimates of total survival have overlapping 95% confidence intervals, the most recent (2002-2009) estimates of the mean for both natural and total survival are numerically lower. Peacock et al. (2012) argues that the decline in the point estimates is real because it is consistent with simulation projections suggesting over-harvest and recent (1999-2008) sea ice decline in Baffin Bay.

Peacock et al. (2012) provide estimates of both natural and total survival, so it was possible to compare simulations from 1997-2013 using survival rates from Taylor et al. (2005) from 1997 to 2013 versus Taylor et al. (2005) survival estimates from 1997-2002, and Peacock et al. (2012) survival estimates from 2003 to 2013 (S1 Fig. S1). The PVA simulation using Peacock et al. (2012) survival rates for the interval 2003-2013 declines to essentially zero (99% of the runs were truncated by the end of the simulation). The Peacock et al. (2012) total survival rate PVA simulation provided a more believable trajectory (S1 Fig. S1), but when Peacock et al. harvest mortality rate (total survival rate-natural survival rate) was used to calculate actual harvest removals in the total survival simulation (S1 Fig. S1), the simulation harvest removals were substantially less than the reported harvest (S1 Tables S1 and S2). We examined the effect that the projected subpopulation decline from 1997 to 2003 had on this inconsistency by also conducting a simulation from 2003 to 2013 from the same 1997 stable age starting conditions (N=2074, SE=266). The discrepancy between annual harvest records and the harvest mortality implied by the Peacock et al. (2012) harvest mortality rate estimate was less for the higher initial subpopulation, however it was still substantial (S1 Table S2).

We explored the hypothesis that recoveries had been under-reported from the Greenland harvest data. We do not consider that harvest recoveries could have been over-reported from the Nunavut harvest data because harvest reporting to a wildlife officer is mandatory, and there is a payment for lip tattoos and ear tags. In Nunavut a vestigial premolar tooth is extracted from harvest and all killed polar bears to allow for aging. This tooth is extracted by the wildlife officer who also inspects for the lip tattoo and usually takes a tissue sample. Harvest reporting in Greenland was the voluntary *Piniarneq* system (filling in a form) until recently (Aars et al., 2006; Obbard et al., 2010). Greenland hunters have a tradition of sharing the hide and meat from harvested polar bears so it is possible that some kills were reported more than once. The *Piniarneq* system involves reporting the annual catch from all species, and reporting is linked to the issuing of hunting licenses for the subsequent year. The issues with this system were summarized in the Greenland Management report to the 2005 Polar Bear Specialist Group (Aars et al., 2006: 141) as follows: “Whether this leads to an under-reporting, over-reporting, or just arbitrary reporting in order to meet requirements when renewing licenses is not clear. An example of sources of error is the report in 2004 of 24 and 10 polar bears reported for Sisimiut and Maniitsoq, respectively (Table 22). Some of these (10 and 5) were reported by hunters with a “part-time” hunting license and are suspected to be of muskoxen. This is currently being investigated by the Greenlandic Ministry of Fisheries (O. Heinrich in litt. 2005).”

The Greenland kill increased dramatically in the early 2000’s. This dramatic increase in reported polar bear kills coincided with public discussions of shared quotas for Canadian and Greenland shared subpopulations of marine mammals. Concurrently the extent of Baffin Bay spring (April-June) sea ice declined (Peacock et al., 2012). Peacock et al.’s (2012) apparent over-estimation of harvest mortality based on recoveries could occur from non-reporting of marked animals in the harvest or from over-reporting of the harvest (which would necessarily be unmarked individuals). We used Fisher’s Exact Test (Faul et al., 2007) to compare the proportion marked in the Greenland BB reported kill to the proportion marked in the Nunavut BB reported kill for the time bin 2003-2009 identified in Peacock et al. (2012) to determine if these samples were drawn from the same subpopulation (S1 Tables S3 and S4). The proportion marked was greater in the Nunavut kill (p<0.0041). We also made the same Nunavut versus Greenland BB recoveries Fisher’s Exact Test comparison for the 1998-2002, 1998-2001, and 1998-2000 time bins. The null hypothesis that recoveries were drawn from the same subpopulation was rejected for the intervals 1998-2002 (p< 0.0103) and 1998-2001 p< 0.0424; but not rejected for the interval 1998-2000 (p<0.3579, power=(1-β)=0.67).

The Burnham (1993) recovery-recapture model used by Peacock et al. (2012) requires that all recoveries are reported and (obviously) that recovery data are correct. We were not able to determine if the divergence in 2003-2009 was due to a failure to report marked recoveries, or an over-reporting the Greenland BB polar bear harvest. However, we believe that over-reporting is the most likely cause of the differential in marked proportion of recoveries between Greenland and Nunavut because that explanation would also explain the inconsistency between simulation results suggesting a precipitous subpopulation decline versus the TEK and anecdotal scientific observations that polar bear numbers have not collapsed in BB.

For these reasons we retained the survival estimates time-referenced to 1997 (Taylor et al., 2005) for our simulations, and suggest that the Peacock et al. (2012) suggestion that survival rates had declined since 2003 is not demonstrated and may be an artifact of sampling issues. We predict that subsequent surveys of BB will confirm that the subpopulation has not declined as the PVA based on the reported kill data suggest it should be (Table 2b; S1 Fig. S1) because the Greenland kill for the interval 2001-2009 (at least) was somehow over-reported.

*Davis Strait (DS)*

Based on movements of tagged animals and, more recently, of adult females with satellite radio-collars, the DS subpopulation is comprised of bears from the Labrador Sea, eastern Hudson Strait, Davis Strait south of Cape Dyer, and along the eastern edge of the Davis Strait-southern Baffin Bay pack ice (Taylor et al., 2001a). When bears occur in the latter area they are subject to hunting by Greenlanders (Stirling & Kiliaan, 1980; Stirling et al., 1980; Taylor & Lee, 1995; Taylor et al., 2001a).

The initial subpopulation estimate of 900 bears for DS (Stirling et al., 1980) was based on a subjective correction from a M-R estimate of 726 bears, which was felt to be too low. Densities of bears were substantially higher in eastern DS than in the Foxe Basin (FB) subpopulation in the survey of the Quebec coast by Crête et al. (1991). In 1993, the Federal/Provincial/Territorial Polar Bear Technical Committee (PBTC) viewed the DS subpopulation estimate to be 1400 bears to account for bias in sampling created by the inability of researchers to survey the extensive area of offshore pack ice (COSEWIC, 2008). The first subpopulation study to cover the entire area was Peacock et al. (2013). Peacock et al. (2013) do not report the stochastic subpopulation growth rate but provide the estimates required to show that the DS subpopulation is increasing or stable at current harvest levels.

Stirling and Parkinson (2006) and Peacock et al. (2013) note that sea ice in Davis Strait does not increase to greater than 50% (which has been used to indicate break-up and freeze-up for other polar bear subpopulations) in some years. Stirling and Parkinson (2006) suggest that (like other seasonal subpopulations) the DS subpopulation is thus likely to experience negative effects from sea ice decline. The current DS subpopulation (N = 2158, SE = 180) has relatively low recruitment, but survival rate estimates are comparable to other polar bear subpopulations in Canada (Peacock et al., 2013; S2 and S3). The DS subpopulation appears to have increased from the mid-1970s (a period of climate warming) until the present, mostly due to reduced hunting, but also because of increased harp seal numbers. The data are insufficient to determine the trajectory of the historical subpopulation increase or identify when recruitment rates began to decline. Sea ice records show a recent decline in summer sea ice and earlier and more extended “open water” beginning in the mid-1990s. The data are insufficient to determine if density effects from the subpopulation increase, or declining sea ice, or both are responsible for the decline in recruitment. A decline in body condition is also suggested (Rode et al., 2012), but the data are insufficient to establish whether the decline is due to reductions in sea ice or increases in subpopulation density. At historical harvest levels (~67 per year) the subpopulation is projected to increase at about 0.8% per year (λ_H_=1.008). With no harvest the subpopulation would increase at about 3.6% per year (λ_N_=1.036). An increase in the harvest to ~82.4 per year would be approximately the maximum sustainable yield (λ_MSY_=1.000).

Genetic, demographic and space-use strata were detected in DS (Peacock et al., 2013). The southern strata (Labrador) appears to contain bears that are in relatively better condition (Rode et al., 2007), perhaps because these bears are closer to harp seal areas during the ice covered season when most polar bear feeding occurs. However, these indications of spatial stratification are individually and collectively insufficient to support identification of a distinct Labrador subpopulation. DS is best considered a single demographic unit for harvest management purposes based on available scientific data.

The change in status from the 2009 PBSG status report (Obbard et al., 2010) is due to an error in the 2009 PBSG status report PVA simulations.

*Foxe Basin (FB)*

Based on 12 years of M-R studies, tracking of female bears with conventional radios, and satellite tracking of adult females in Western and Southern Hudson Bay, the Foxe Basin (FB) subpopulation is thought to comprise a demographic unit in Foxe Basin, northern Hudson Bay, and the western end of Hudson Strait (Taylor & Lee, 1995; Taylor et al., 2001a). During the ice-free season, polar bears concentrate on Southampton Island and along the Wager Bay coast; however, significant numbers of bears also occur on the islands and coastal regions throughout the Foxe Basin area. Crête et al. (1991) found relatively few bears of the FB subpopulation along the Quebec shore during the ice-free season. A 1996 total abundance estimate of 2200 (SE = 260) was developed from a M-R analysis based on tetracycline biomarkers (Taylor & Lee, 1994; Taylor et al., 2006b). The marking effort was conducted during the ice-free season, and distributed throughout the entire area. The abundance estimate is believed to have been accurate, and was supported by TEK that the subpopulation had been reduced by harvest but was still abundant. Simulation studies suggest that harvest quotas prior to 1996 reduced the subpopulation from approximately 3000 in the early 1970s to 2100 bears in 1996. Harvest levels were reduced in 1996 to permit recovery of this subpopulation, provided that harvest in Quebec did not increase. Simulation studies using demographic rate estimates from BB (Taylor et al., 2005) and the observed mean harvest rate predict an increase in numbers to 2780 (SE = 806) by 2010.

TEK suggested that the FB subpopulation had increased since 1996 (McDonald et al., 1997). For example, on Southampton Island hunters often fill their quota in a matter of days (McDonald et al., 1997). However, TEK from the Ivujivik, Quebec area suggested a decrease in polar bear numbers. One hypothesis proposed to explain this observation is that ocean currents in the region are now weaker, allowing bears to become distributed more evenly on the ice during mid-winter rather than congregating at the mouth of Hudson Strait (McDonald et al., 1997). After consultations with Foxe Basin Inuit, Nunavut increased the harvest quota in 2004 to a level consistent with a subpopulation size of 2300 bears (109 bears per year). An aerial survey conducted in 2010 estimated the number of FB polar bears had increased to 2580, SE = 278 (Stapleton et al., 2016), but did not estimate current subpopulation growth rate.

Recruitment and survival rates have not been estimated for FB. Meta-analysis employing the adjacent BB demographic rates appeared to over-estimate FB subpopulation performance by a small and insignificant margin. We reduced BB adult litter production rates from 1.0 to 0.85 (i.e., 85% of available-to-reproduce females produced litters) to produce a simulation that resulted in ~2700 individuals in 2010, and used this empirically corrected (to match the Stapleton et al. (2016) aerial survey estimate) Baffin Bay demographic rates for the FB PVA (Tables 2a and 2b). While this meta-analysis approach is supported by the consistency of the simulation outcome with the aerial survey result, other combinations of recruitment and survival rates could have been identified that provided the same result. The qualitative result of subpopulation increase is not in doubt; however, PVA estimates of the associated uncertainty associated with simulation results should be viewed with caution.

Effects of climate change on the FB subpopulation of polar bears have not been evaluated scientifically. As Foxe Basin is immediately north of Western Hudson Bay and has experienced earlier timing of break-up of sea ice in similar fashion as the rest of Hudson Bay, Stirling and Parkinson (2006) predict a decline in FB polar bears similar to the report for the Western Hudson Bay (WH) subpopulation (Regehr et al., 2007a, 2007b), and various previous polar bear status reports (Obbard et al., 2010; PBTC, 2010). The 2009 PBSG status report (Obbard et al., 2010) lists FB as data deficient. However, the observed increase in number from 2200 in 1996, to 2580 in 2010; suggests that the subpopulation has increased as per the Nunavut harvest management goals during a period of climate warming. Unfortunately, aerial survey does not provide any estimate of current demographic performance.

*Gulf of Boothia (GB)*

Boundaries of the subpopulation of polar bears inhabiting the Gulf of Boothia were largely based on movements of tagged bears (Taylor & Lee, 1995), movements of collared females in the Gulf of Boothia and adjacent areas (Taylor et al., 2001a), and information from Inuit hunters about how local conditions influence the movements of polar bears. Hunting in the Gulf of Boothia increased from historic levels through the 1970s (Brice-Bennett, 1976); however, the initial quota established by the Government of the Northwest Territories in the Gulf of Boothia was less than the maximum sustainable yield. Local hunters reported that the subpopulation had increased during the 1980s after results of Furnell and Schweinsburg (1984) suggested GB abundance was about 300 bears (based on limited sampling of a small portion of the GB area). Based on Inuit knowledge, recognition of past sampling deficiencies, and an increased understanding of polar bear densities in other areas, the interim subpopulation estimate in the 1990s for the GB subpopulation was 900 bears (M. Taylor, pers. comm.1986-2008). Following completion of a M-R inventory in spring of 2000, the subpopulation was estimated to number 1528 bears (SE = 285; Taylor et al., 2008c). Recruitment and survival rates (S2 and S3) were estimated to be relatively high. In 2004, harvest quotas were increased by the Government of Nunavut to 74 bears per year.

*Kane Basin (KB)*

Based on movements of adult females equipped with satellite radio-collars and recaptures of tagged animals, the boundaries of the KB subpopulation include the North Water Polynya (to the south), and Greenland and Ellesmere Island to the west, north, and east (Taylor et al., 2001a). Polar bears in KB do not differ genetically from those in BB (Paetkau et al., 1999, their Tables 1 and 2). Prior to 1997, this subpopulation was essentially unharvested in Canadian territory because of its distance from Grise Fiord, the closest Canadian community, and because conditions for travel in the region are typically difficult. However, bears from this subpopulation have occasionally been harvested by hunters from Grise Fiord (since 1997) and harvest continues on the Greenland side of KB. In some years, Greenland hunters also harvest polar bears in the Canadian portion of Kane Basin (i.e., western Kane Basin and Smith Sound) (Rosing-Asvid and Born, 1990; Taylor et al., 2008a).

Few polar bears were encountered along the Greenland coast during M-R surveys between 1994 and 1997, presumably because of harvest pressure by Greenland hunters. The current and only estimate of the KB subpopulation is 164 bears (SE = 35; Taylor et al., 2008a). The best estimate of the Greenland kill is 10 bears per year during 1999–2003 (Born, 2005; Born and Sonne, 2005). However, the actual number being taken by Greenland hunters is uncertain (Rosing-Asvid, 2002; Born and Sonne, 2005) and must be validated before a reliable estimate of KB removals can be developed. The Canadian quota for this subpopulation is five bears per year. The annual combined Canadian and Greenlandic take of 10–15 bears from this subpopulation is unsustainable (Table 2a). Although the habitat appears suitable for polar bears on both the Greenland and Canadian sides of Kane Basin, the density of bears on the Greenland side is much lower than on the Canadian side. The estimates of recruitment and natural (no harvest) survival rates indicate that the subpopulation would most likely continue to decline to extinction even without hunting. The long-term persistence of the KB subpopulation could be understood as a source-sink situation with BB providing immigrants to keep the subpopulation from being exterminated. Another non-exclusive explanation is that the small sample size, influx of non-resident bears, and spatially selective over-hunting resulted in demographic rate estimates that may not be representative of a stable, well-managed subpopulation (Taylor et al., 2008a).

Co-management discussions regarding the hunting of polar bears have been ongoing between Greenland and Canada. Greenland enacted a quota system on 1 January 2006 (West Greenland harvest is not to exceed 100 bears per year, PBTC, 2006); however, because KB, BB, and DS are treated as a single unit for management purposes by Greenland, it is unclear whether reductions in the harvest of bears in KB will result from the establishment of this quota. The mean kill of polar bears in KB has been 10 bears per year for hunters of Greenland in recent years, and <1 for hunters of Nunavut (PBTC, 2006, 2010).

*Lancaster Sound (LS)*

The central and eastern portion of the LS subpopulation is characterized by high productivity and thus high densities of ringed seals and polar bears (Schweinsburg et al., 1982; Kingsley et al., 1985; Welch et al., 1992; Taylor et al., 2008b). Inuit hunters of Resolute, Grise Fiord, and Arctic Bay have all historically hunted polar bears in Lancaster Sound (Brody, 1976; Riewe, 1976). The western third of this region (eastern Viscount Melville Sound) is dominated by multi-year ice and apparently low biological productivity, leading to low densities of ringed seals (Kingsley et al., 1985). In the spring and summer, densities of polar bears in the western third of the area are relatively low; however, as break-up occurs, polar bears move west to summer on the multi-year pack (Taylor et al., 2001a, 2008b).

M-R data and data on movements of adult females fitted with satellite radio-collars have been collected for bears of LS (Taylor et al., 2001a, 2008b). The current abundance estimate of 2541 bears (SE = 391) is based on an analysis of M-R data current to 1997 (Taylor et al., 2008b). This estimate is considerably larger than the 1979 estimate of 1031 ± 236 bears (mean ± 95% CI) published by Schweinsburg et al. (1982); however, Schweinsburg et al. (1982) sampled from only a portion of the subpopulation area (study area) and Taylor et al. (2008b) covered the entire area. It is not possible to unambiguously determine if the difference in estimates is due to an under-estimate from the 1979 study caused by sampling only a portion of the area, or a subpopulation increase. However, harvest rates in this area have remained approximately constant, therefore the most likely explanation is that the 1979 estimate was biased low as other partial coverage estimates have been (e.g., BB, DS, GB). Schweinsburg et al. (1982) focused on a much smaller area that extended into northern Baffin Bay, compared to the entire LS area (Fig. 1) surveyed by Taylor et al. (2008b).

*M'Clintock Channel (MC)*

The current boundaries for the MC subpopulation are based on recoveries of tagged bears and movements of adult females with satellite radio-collars in adjacent areas (Taylor & Lee, 1995; Taylor et al., 2001a). These boundaries appear to be a consequence of large islands to the east and west, the mainland to the south, and the multi-year ice in Viscount Melville Sound to the north. A 6-year M-R study in the mid-1970s (Furnell and Schweinsburg, 1984) reported ~1100 animals for an area that overlapped both M’Clintock Channel and the Gulf of Boothia (Fig. 1). The PBTC “corrected” the MC estimate to 900 bears (COSEWIC, 2008) possibly based on approximate extrapolation of apparent densities from the Furnell and Schweinsburg (1984) study. During community consultations in 1993, local hunters suggested reducing the MC estimate to ~700 animals because of a perceived decline in abundance in the southern and western porting of the subpopulation at current harvest rates. The revised TEK estimate of 700 was accepted by the PBTC as an interim value until a new study could be completed, and subsequent MC polar bear quotas and status determinations were based on the TEK estimate. No confidence intervals were identified for either estimate.

Following completion of a M-R inventory in spring of 2000, the subpopulation was estimated to number only 284 bears (SE = 59.3; Taylor et al., 2006a). The legal harvest (averaging 34.0 bears per year from 1979–1999) for MC, which was based on the 700 estimate was not sustainable. The Government of Nunavut implemented a moratorium on hunting for the 2001/2002 and 2002/2003 hunting seasons. The current annual quota for MC was identified as a number that would permit some harvesting and still allow subpopulation growth (i.e., three bears per year). The subpopulation is presumed to be increasing (Tables 2b and 3).

Scientific data, which suggests low abundance of polar bears in MC due to over-harvest, is supported by TEK. Recently, hunters of Gjoa Haven reported that the number of bears near their community had declined over the past 30 years (Keith et al., 2005). Other areas where decreased numbers of polar bears have been reported include the Royal Geographical Society Islands, Pasley Bay, northern King William Island, Gateshead Island, Larsen Sound, and the M’Clintock Channel itself (Atatahak & Banci, 2001). Inuit suggest that polar bears are no longer present in the Queen Maud Gulf area (Keith et al., 2005). Inuit hunters also report a decline in the number of adult male bears in MC but that large males can be found further to the north (Atatahak & Banci, 2001; Keith et al., 2005). This finding is consistent with what one could expect from a male-selective over-harvest (Taylor et al., 2008d).

In addition to unsustainable harvesting, recent changes in habitat and disturbance by humans have been identified by Inuit as potential reasons for the reduced abundance of bears in MC (Keith et al., 2005). One noted habitat change has been the recent absence of multi-year ice and icebergs, which may reduce the quality of habitat because of tide crack (breathing hole) formation at the edges of these bergs and multi-year ice floes. Human disturbances such as the construction of Distant Early Warning (DEW) line sites, construction of Inuksuit, and noise from aircraft and snowmobiles are also thought to have contributed to the low density of bears around Gjoa Haven (Keith et al., 2005).

*Northern Beaufort Sea (NB)*

Studies of polar bears in the Northern Beaufort Sea have used telemetry and mark and recapture programs at regular intervals since the early 1970s (Stirling et al., 1975, 1988; DeMaster et al., 1980; Lunn et al., 1995). Results suggested that there were separate subpopulations in the Northern and Southern Beaufort Sea areas and not a single subpopulation as was initially thought (Stirling et al., 1988; Amstrup, 1995; Taylor & Lee, 1995; Bethke et al., 1996; Taylor et al., 2001a). An abundance estimate of 1200 polar bears in the late 1980s (Stirling et al., 1988) was believed to be unbiased, but Stirling et al. (2011) now estimate that the number throughout the 2000s was about 980 (SE = 77.5) in the NB subpopulation. The Stirling et al. (2011) constancy of NB subpopulation estimates is inconsistent with the trend from simulations using the survival estimates from the same paper and recruitment estimates from COSEWIC (2008). The historical permitted harvest (65) exceeds a generic estimated sustainable harvest (Taylor et al., 1987c) by 65-44= 21. However, the historical harvest has been less than 44 per year (COSEWIC, 2008; Table 2a) suggesting that the actual kill has remained within sustainable limits. The 2008 COSEWIC status report (COSEWIC, 2008) and the 2009 PBSG status report (Obbard et al., 2010) both list the NB subpopulation as constant, but also estimate the number at 1200. Using the same PVA criteria used for all other subpopulations, we identify the NB subpopulation as declining, but acknowledge that current estimates of survival and subpopulation numbers are ambiguous. The historic Inuvialuit harvest quota from NB was 59 and Nunavut harvest quota from NB was 6. NB and Southern Beaufort Sea (SB) boundary changes have recently been implemented and resulted in new quotas based on new subpopulation estimates that recognize the incomplete sampling effort.

Hunting of polar bears of the NB subpopulation has historically focused on the Amundsen Gulf (Usher, 1976; Farquharson, 1976), although the western coast and associated sea ice of Banks Island are also important for Inuit hunters (Usher, 1976). In a 2001 interview for the Paulatuuq Oral History project, an elder hunter suggested that the subpopulation in the area had been stable over the past 30 years (Parks Canada, 2004). One explanation for the inconsistency between simulation subpopulation trajectories and Stirling et al. (2011) subpopulation estimates is that Stirling et al. (2011) survival rates were biased low due to un-modeled capture heterogeneity due to incomplete sampling of the subpopulation area. Capture bias would also have causes the subpopulation estimates to be biased low. For this reason, the relationship between scientific perspectives and TEK is ambiguous for the NB.

Analyses, using data from satellite tracking of female polar bears and spatial modelling techniques, suggested that the boundary between the NB and the SB subpopulations needed to be adjusted by expanding the area occupied by bears from NB and reducing that of SB (Amstrup et al., 2004). After consultation between the Inuvialuit, the Inupiat, affected Inuvialuit communities and affected Nunavut Kitkmeot Inuit, the SB-NB boundary was shifted west to 133^o^. Stirling et al. (2011) estimated the NB subpopulation was about 980. However, the Inuvialuit Wildlife Management Advisory Committee (WMAC), guided by the suggestion in Stirling et al. (2011) that 1200-1300 (point estimates when 2006 data excluded) could more accurately reflected the current number of polar bears in the NB subpopulation, corrected the Stirling et al. (2011) estimate to 1400 to account for the suspected bias and for areas that were not surveyed during the study. The addition of the 310 bears due to the boundary change raised the final NB estimate to 1710. A new NB quota of 77 (Inuvialuit 71, Nunavut 6) was identified based on the historical estimate of maximum sustained yield (MSY) of 4.5% per year at a 33% female and 67% male sex ratio. This change indicates that NB TEK agrees with Stirling et al. (2011) that numbers have remained stable or increased. The 2013 PBTC status table (PBTC, 2013) designates the NB subpopulation as stable based on TEK. Additionally, the use of the historical MSY index suggests that TEK does not support a decline in subpopulation productivity in the NB. This change in quota occurred after the 2012/2013 harvest season so our analyses used the Stirling et al. (2011) estimates.

*Norwegian Bay (NW)*

The polar bear subpopulation of Norwegian Bay is bounded by multi-year ice to the west, islands to the north, east, and west, and polynyas to the south (Taylor et al., 2001a; Taylor et al., 2008b). Based on data from M-R studies and satellite radio-tracking of adult females, it appears that most bears concentrate along coastal tide cracks and ridges in the northern, eastern, and southern regions of Norwegian Bay (Taylor et al., 2001a). The preponderance of multi-year ice through most of the central and western areas contributes to low densities of ringed seals (Kingsley et al., 1985) and, consequently, low polar bear density. Grise Fiord hunters reported high concentrations of polar bears in Norwegian Bay during the early 1970s (Riewe, 1976). Taylor et al. (2008b) estimate the number in 1997 in the NW subpopulation was 190 bears (SE = 48.1). Estimates of survival rates (S2) for NW are derived from pooled LS and NW data because these two subpopulations are adjacent and because the number of bears captured in Norwegian Bay was too small for reliable survival estimates (Taylor et al., 2008b). Polar bears in Norwegian Bay are likely to benefit from a warming climate (at least over the short term), which may increase abundance of and accessibility to seals (Derocher et al., 2004).

The harvest quota for the NW subpopulation was reduced to four bears (3M: 1F) in 1996 and remains at this level today. The harvest has been all male for the last five years (Tables 2a; S4 Table S3) and mainly male since 1996 because most harvesting in Norwegian Bay is guided sport hunts. The PBSG status report (Obbard et al., 2010) identified the NW subpopulation as declining with high certainty; however, their PVA short-term simulations (five years) assumed that a portion of the harvest was female based on historical data and did not allow the subpopulation to increase as the females and thus subpopulation productivity increased (Taylor et al., 2008d). The COSEWIC (2008) status report listed the subpopulation as “stable” based on a consideration of the sex ratio of the harvest and the length of time required for the subpopulation trend from an all-male harvest to become clear. TEK from NW suggests no change in polar bear subpopulation numbers there (M. Taylor, pers. comm. 1986-2008).

*Southern Beaufort Sea (SB)*

The subpopulation of polar bears inhabiting the Southern Beaufort Sea is shared between Canada and the United States (Alaska). On the Canadian side of the border, the historical harvest of bears has been relatively light. The subpopulation experienced an increase in hunting activity in the late 1950s due to an increase in fur prices (Usher, 1976); however, by the mid-1970s polar bears were only killed opportunistically during hunts for other species by Aklavik and Inuvik hunters (Usher, 1976). Hunters of Tuktoyaktuk recall people from their community also hunting polar bears during this time (Frank Pokiak, Chair, Inuvialuit Game Council, letter to COSEWIC Terrestrial Mammals Specialist Subcommittee, 19 January 2007; COSEWIC, 2008). The Cape Bathurst area was reported to be an important area for hunting polar bears (Usher, 1976).

During the early 1980s, radio-collared individuals were tracked from the Canadian portion of the Southern Beaufort Sea into the eastern Chukchi Sea of Alaska (Taylor, 1987c; Amstrup et al., 1986; Amstrup & DeMaster, 1988). Telemetry data combined with re-sightings of tagged individuals suggested that bears of the Southern Beaufort Sea comprised a single subpopulation with an eastern boundary between Paulatuk and Baillie Island, NWT, Canada, and a western boundary near Icy Cape, Alaska (Taylor, 1987c; Amstrup et al., 1986; Amstrup and DeMaster, 1988; Stirling et al., 1988). Recognition that bears were shared by Canada and Alaska prompted the *Polar Bear Management Agreement for the Southern* *Beaufort Sea* (the *Agreement*) (Treseder and Carpenter, 1989; Nageak et al., 1991). The *Agreement*, between the Inupiat hunters of Alaska and the Inuvialuit hunters of Canada, was ratified by both parties in 1988. The *Agreement* included provisions to protect bears in dens and females with cubs, and stated that the annual sustainable harvest from the Southern Beaufort Sea would be shared between the two jurisdictions. Harvest levels were to be reviewed annually in light of the best scientific information available (Treseder & Carpenter, 1989; Nageak et al., 1991). Brower et al. (2002) evaluated the effectiveness of the *Agreement* after the first 10 years and concluded that; overall, it had been successful in ensuring that the total harvest and the harvest of adult females remained within what were thought to be sustainable limits. The Inuvialuit and Inupiat harvest quota from SB was 80 bears but the Joint Commissioner recommended a reduction to 70 in 2013 while NB/SB boundary changes were under discussion between co-management authorities.

Taylor et al. (1987c) and Amstrup et al. (1986) estimated the size of the SB subpopulation to be approximately 1800 bears, with a minimum and maximum of 1300 and 2500 bears, respectively. Amstrup et al. (2001), claiming the previous estimates were unreliable, estimates the total SB subpopulation to number to be >2500 as of 1998. However, Regehr et al. (2006) recalculates that the Amstrup et al. (2001) estimate was actually 2185, and provides a current (2006) SB estimate of 1526 (SE=164) polar bears, and concludes that no trend could be determined. The no-trend finding stands in contrast to PVA analyses that suggest the SB is declining (Hunter et al., 2010; Table 3).

Rates of survival and recruitment have recently been developed for bears of the Southern Beaufort Sea (Regehr et al*.*, 2006, 2010; PBTC, 2007; COSEWIC, 2008; Obbard et al., 2010; S2 and S3). Based on published recruitment and survival rates, the combined U.S.-Canadian harvest of bears in the Southern Beaufort Sea would cause the subpopulation to decline (Hunter et al., 2010; Tables 2a and 2b). Our simulations indicate that if the harvest was reduced to zero, the SB subpopulation would continue to decline to extirpation (Table 4). This perspective is not consistent with current TEK or management perspectives for this subpopulation.

After consultation with the Inupiat, affected Inuvialuit communities and affected Nunavut Kitkmeot Inuit, the NB-SB boundary was shifted west to 133^o^ and the Regehr et al. (2006) SB subpopulation estimate was reduced by 310 individuals to about 1200. A new SB quota of 56 was identified based on the historical estimate of maximum sustained yield (MSY) of 4.5% per year at a 33% female and 67% male sex ratio. This change brings the SB quota into agreement with the Regehr et al. (2006) subpopulation estimate that was less than (but not significantly different from) the previous (Amstrup et al. 1986) estimate of 1800 by 274 individuals. The 2013 PBTC status table (PBTC, 2013) designates the SB subpopulation as stable based on TEK. Additionally, the use of the historical MSY index suggests that user groups feel there has been no decline in SB subpopulation productivity. The boundary and quota change occurred after the 2012/2013 harvest season so our analyses used the 2006 subpopulation estimate (Regehr et al., 2006).

*Southern Hudson Bay (SH)*

Both coastal surveys (Stirling et al., 2004) and Inuit hunting in the SH subpopulation of polar bears reported an increase in the number of bears that have historically occurred in the area (McDonald et al., 1997). The offshore islands of eastern Hudson Bay apparently did not have any bears 50 years ago, and the species was rare around Inukjuak, only appearing “recently” (McDonald et al., 1997). Similarly, in Sanikiluaq, it was rare to kill a polar bear in the 1960s but now the community’s annual quota is filled in approximately three weeks, with increased observations of bears coming into the community (L. Arragutainaq, pers. comm. 2006). In 1986, Crête et al. (1991) found relatively high numbers of bears on the Twin Islands in James Bay during the ice-free season. Cree in western James Bay report increased aggressiveness among bears and an increase in litter size (McDonald et al., 1997). Communities along the Hudson Bay and James Bay coasts in Ontario report an increase in bear encounters and property damage caused by polar bears (M. Carpenter, pers. comm. 2006; A. Solomon, pers. comm. 2006; P. Kapashesit, pers. comm. 2006; COSEWIC, 2008). In the past five years, polar bears have also been observed to travel more frequently during the open water season all the way to the Moosonee area of southern James Bay (approximately one sighting per year). Previously, bears were observed around Moosonee roughly once in five or six years (P. Kapashesit, pers. comm. 2006; A. Solomon, pers. comm. 2006; COSEWIC, 2008). Explanations offered for observations of higher numbers of bears include potential immigration of bears in response to increased ringed seals in the region, extended ice coverage in the area, and under-harvesting by the four aboriginal groups (Nunavut Inuit, Ontario Cree, and Quebec Inuit and Quebec Cree) of the SH subpopulation.

Boundaries of the SH subpopulation of polar bears are currently based on data from movements of marked bears of all sexes and telemetry studies of females (Jonkel et al., 1976; Kolenosky & Prevett, 1983; Kolenosky et al., 1992; Taylor & Lee, 1995). Crompton (2004) suggests that the current boundaries that define the SH subpopulation may need to be revisited, as she observed at least three breeding groups in the southern portion of Hudson Bay (including James Bay). However, the notion of three distinct breeding groups in such a small area devoid of any barriers to movements is inconsistent with other genetic information (Paetkau et al., 1999) and the great mobility of polar bears (COSEWIC, 2008).

Obbard et al. (2007) indicates there has been no significant decline in abundance of polar bears in SH since the 1980s. Obbard et al. (2007) estimated abundance of polar bears in SH as 641 (95% CI: 401–881) in 1986 and 681 (95% CI: 401–961) in 2005. These estimates are lower than previously stated for the SH (e.g., 1000 bears). Obbard et al. (2007:6) states that “the goodness of fit analysis did not detect any over-dispersion in the SH data nor any heterogeneity that could not be explained with covariates”. However, the 2009 PBSG status report (Obbard et al., 2010) states that the 1986 estimate was 634 (95% CI 390-878) and the 2005 estimate was 673 (95% CI: 396-950), and cites Obbard et al. (2007) as the reference. In contrast to the Obbard et al. (2007) determination that the estimates were accurate, the PBSG status report (Obbard et al., 2010) states that 2007 estimates are likely an underestimate because of lack of complete coverage of the subpopulation (e.g., only coastal coverage on Ontario’s north coast and no coverage in James Bay). Obbard et al. (2007) and the PBSG 2009 status report (Obbard et al., 2010) lists the current SH estimate as 900 individuals. We find these “corrections” to be unsupported and arbitrary. However, taking the middle of the range given for James Bay as: 90 (SE = 10), and adding this value to the “accurate” Obbard et al. (2007) estimate for north coastal Ontario (681, SE = 280) yields 771 (SE = 300). The SH subpopulation is listed as stable in both the COSEWIC (2008) status report and the PBSG 2009 status report (Obbard et al., 2010) essentially on the strength of no trend between the 1985 and 2005 north coastal M-R estimates. When considered in a PVA context neither the subpopulation estimates nor the survival rate estimates from either of these studies are consistent with “stable” status for SH polar bears at existing harvest levels, suggesting that any perspective on trend drawn from these data is speculative.

Stirling et al. (2004), in their recent analysis of coastal survey data, suggested that the abundance of polar bears in SH has been increasing in recent years, although numbers in WH have remained about constant. A decline in body condition in the WH polar bear subpopulation has been correlated to a decline in sea ice, survival, and recruitment (Stirling et al., 1999; Regehr et al., 2007a, 2007b). A decline in body condition was also documented for the SH subpopulation when comparing bears captured in 1984–1986 with those captured in 2000–2004 (Obbard et al., 2006; PBTC, 2006; Obbard et al., 2007); however, unlike the WH subpopulation; there has been no concurrent decline in sea ice, survival or recruitment estimates (Obbard et al., 2007). Increased density may be the cause of the decline in condition; however, that notion is not supported by the subpopulation estimates which, in contrast to Stirling et al. (2004), show no trend. Although overall abundance in SH appears to have been stable in at least north coastal Ontario since the 1980s, both the 1986 and 2005 subpopulation estimates are ambiguous because only portions of the SH subpopulation summer retreat area were sampled. Thus both survival and subpopulation estimates may be biased and low, including the summed value for subpopulation numbers listed above.

A 2012 aerial survey of the SH subpopulation (Obbard et al., 2013) estimated the subpopulation to number 969 (SE=202), suggesting that mark recapture results under-estimate subpopulation number and survival rates, and thus under-estimate subpopulation trend.

*Viscount Melville Sound (VM)*

Only in the past 30 years have polar bears of the Viscount Melville Sound experienced modern levels of hunting pressure. Farquharson (1976) noted that by the mid-1970s, hunters from the Holman area had expanded their traditional hunting range to kill polar bears along the western and northern coasts of Victoria Island to Wyanniatt Bay. At the same time, Inuit from Cambridge Bay began travelling by land or air to reach northern Victoria Island to hunt polar bears in Hadley Bay. In response to increased interest in hunting bears of the VM subpopulation, the Government of the Northwest Territories established quotas. When quotas were originally allocated in the 1970s, the size and productivity of the VM subpopulation was overestimated. Polar bear density is lower in VM compared to other regions because of large expanses of multi-year ice and low densities of ringed seals (Kingsley et al., 1985). The consequence of overestimating abundance when quotas were first established was substantial over-harvest of bears in the region during the 1980s and early 1990s (e.g., 1985–1990 mean of 19.6 bears per year; Taylor et al., 2002).

A five-year moratorium on hunting was enacted in 1994/1995. Hunting resumed in 1999/2000 with an annual quota of four bears. In 2004/2005 the annual quota was increased to seven bears per year (Northwest Territories 4, Nunavut 3) to accommodate hunters on both sides of the new territorial border. Polar bear numbers in the VM are anticipated to still be increasing with this increase in quotas. The current (increased quota) kill is less that the sustainable yield using the 1996 subpopulation estimates with for the PVA (Table 2a).

A five-year study of movements and size of the VM subpopulation of polar bears using satellite telemetry and mark and recapture sampling was completed in 1992 (Messier et al., 1992, 1994; Taylor et al., 2002). Current boundaries are based on observed movements of females with satellite radio-collars and movements of bears tagged inside and outside of the study area (Bethke et al., 1996; Taylor et al., 2001a). The published 1996 abundance estimate of 215 bears (SE = 58) in Taylor et al. (2002) was based on the 1993 estimate plus three years of simulated subpopulation growth. Polar bears in VM are likely to benefit from a warming climate (at least over the short-term), which may increase the abundance and accessibility of seals by reducing amounts of multi-year ice.

*Western Hudson Bay (WH)*

The distribution, abundance, and boundaries of the WH subpopulation of polar bears have been studied since the late 1960s (e.g., Stirling et al., 1977; Derocher & Stirling, 1990, 1992, 1995a, 1995b; Taylor & Lee, 1995; Lunn et al., 1997, 2006). Between 60–80% of adults have been marked at any given time and there are extensive records from mark recapture studies and the return of tags from bears killed by Inuit hunters, and from the ongoing and long-term Polar Bear Alert Program of the Government of Manitoba. This subpopulation appears to be geographically segregated during the open-water season, although it mixes with those of SH and FB on the Hudson Bay sea ice during the winter and spring (Stirling et al., 1977; Derocher & Stirling, 1990; Stirling & Derocher, 1993; Taylor & Lee, 1995; Stirling et al., 2004).

Nirlungayuk (G. Nirlungayuk, pers. comm., 2008; COSEWIC, 2008) summarizes Nunavut Inuit TEK that polar bear numbers in the areas of Western Hudson Bay are considerably higher at the present time than any time previously. One explanation for the increase is supplemental feeding by garbage (around Churchill). For example, prior to the increase in the population of humans in Churchill in the 1940s, polar bears were best hunted nearer Wager Bay, Southampton Island, and Coates Island. After polar bear hunting regulations were initiated in 1968, if people wanted to be guaranteed a polar bear, they would travel down to south of Arviat. Observing polar bears by boat in Western Hudson Bay was once a rare event; now “lots of bears are there.” Concurrently but contrary to the scientific M-R re-assessment of abundance (below), Inuit along the western coast of Hudson Bay recently reported seeing greater numbers of polar bears, which they interpreted as evidence of an increasing subpopulation (McDonald et al., 1997; Dowsley & Taylor, 2006b). Polar bears have been reported as “numerous” at Chesterfield Inlet in September and have been increasing in that area since 1988. Bears have been present for several years near Arviat, from September to December, but have recently increased in number according to TEK, especially in September.

The dangers posed by on-shore polar bears in the region are a concern to Inuit, and numbers are believed to be “too high” by some people (G. Nirlungayuk, pers. comm. 2008; COSEWIC, 2008). Encounters in the region have increased through the 1970s and 1980s. Since the 1980s, Arviat has been warning hunters to not to go out alone to ensure safety from bears. Nunavut Tunngavik Incorporated (NTI) recently collaborated with five experienced hunters from communities in Western Hudson Bay to complete a series of interviews and a workshop (NTI, 2005). TEK indicates an increasing number of bears in the Arviat area since the 1970s and around Whale Cove and Rankin Inlet since the 1980s. This increase in numbers has also been noted by Inuit of Chesterfield Inlet. In Arviat, the recent increase has been noted in all seasons except winter, while Inuit of other areas report an increase in all seasons. In the Chesterfield Inlet area, groups (gatherings) of polar bears have been observed recently, something that was apparently rare in the past.

Over the past 30 years, the condition of adults and the proportion of independent yearlings captured during the open-water season have declined significantly in the WH subpopulation (Derocher & Stirling, 1992, 1995b; Stirling & Lunn, 1997; Stirling et al., 1999; COSEWIC, 2008). Over the same period, the average date of break-up of the sea ice has advanced by three weeks (Stirling et al., 1999, 2004; Ferguson et al*.*, 2005). Stirling et al. (1999) documented that the earlier the timing of break-up, the poorer the condition of adult females. Inuit confirm the scientific information on changes in sea ice in Western Hudson Bay (G. Nirlungayuk, pers. comm. 2008; COSEWIC, 2008).

The number of polar bears in the WH subpopulation was recently estimated by Regehr et al. (2007a, 2007b). Regehr et al. (2007a, 2007b) estimated that abundance has declined from 1194 (95% CI = 1020–1368) to 935 (95% CI = 794-1076) between 1987 and 2004, a reduction of approximately 22%. Progressive declines in the condition and survival of cubs, subadults, and bears 20 years of age and older contributed to the decline in the size of the subpopulation. Once the subpopulation productivity began to decline, the existing harvest was no longer sustainable, and thus contributed to the reduction in the size of the WH subpopulation. The harvest sex ratio of 2M:1F in WH has resulted in a sex ratio that is 58% female and 42% male (Derocher et al., 1997b, Taylor et al., 2008d).

In summer 2007, the Government of Nunavut conducted a M-R survey of bears from Churchill to Chesterfield Inlet to determine whether or not there were large numbers of bears along the Kivalliq coast during the summer as suggested by TEK (Peacock & Taylor, 2007). The survey included those areas identified by TEK as being areas where polar bears were becoming more common. A total of 25 bears were captured during the three-day survey that ended north of the CWS study area. The proportion of marked individuals in the capture sample (*p* = 0.46, SE = 0.11) was lower but not statistically different from the proportion of marked animals in the Canadian Wildlife Service (CWS) capture sample (*p* = 0.59, SE = 0.01). However, the mean time since marked bears were last handled was significantly greater for marked polar bears captured outside the CWS “study area”. Statistical power was low for this analysis due to the small sample size and because the capture effort only extended to the mouth of the Seal River (~59 degrees latitude). These results suggest that actual numbers of bears in WH and annual survival rates could thus be higher (due to un-modelled heterogeneity) than estimated by Regehr et al. (2007a, 2007b). Peacock and Taylor (2007) recommend that in future years, CWS capture teams work north to Arviat to capture polar bears in the entire area where polar bears summer.

Climate change reductions to sea ice with consequent reductions in body condition, survival and recruitment and resulting over-hunting is considered to be the major threat to the WH subpopulation. The subpopulation is believed to be declining at a substantial rate (Regehr et al., 2007a, 2007b; Table 2b), and the quota for hunting polar bears in WH was reduced to eight animals in 2008–2009; however, PVA analysis using demographic rates from Regehr et al. (2007a, 2007b) indicate that the WH subpopulation will decline with no removals for control or harvest.

TEK perspectives and scientific perspectives are qualitatively different for WH. Currently the WH subpopulation is the only subpopulation of 19 circumpolar subpopulations where a decline due to climate warming can be demonstrated (Regehr et al., 2007a, 2007b; Obbard et al., 2010). An aerial survey covering the entire WH summer retreat area was undertaken in fall of 2011. The result of the 2011 aerial survey (N=1013, SE=151) confirms Inuit knowledge that this subpopulation has not declined and may have increased (Stapleton et al., 2014). Independent of the aerial survey results, it does appear that body condition, survival rate, and recruitment rates have declined. An alternative hypothesis to climate mediated decline in sea ice as the main cause for reduced subpopulation productivity is density effects as suggested by the asymptotic behaviour of the WH abundance index provided by annual beach transects (Stirling et al., 2004).

**S1 Table S1.** Baffin Bay (BB) mortality rates were applied to this RISKMAN stable-age distribution as a consistency check between the Peacock et al., 2012 estimated survival rates and the BB reported harvest (S1 Table S2). The BB RISKMAN stable age-distribution uses the 1997 estimate of abundance (N=2074, SE=265) from Taylor et al., 2005.

| **Age** | **Males** | **Females Unencumbered** | **Females Encumbered** | | | | | |
| --- | --- | --- | --- | --- | --- | --- | --- | --- |
| 0 | 161.9169 | 166.5149 |  |  |  |  |  |  |
| 1 | 100.9874 | 104.4790 |  |  |  |  |  |  |
| 2 | 63.04853 | 65.6201 |  |  |  |  |  |  |
| 3 | 52.61324 | 52.8113 |  |  |  |  |  |  |
| 4 | 43.90512 | 38.4224 | 1.6851 | 2.3951 |  |  |  |  |
| 5 | 36.63829 | 4.3424 | 11.2512 | 15.9915 | 1.7609 | 0.8602 |  |  |
| 6 | 35.5692 | 5.9151 | 1.7174 | 2.4410 | 13.9899 | 6.8340 | 1.4919 | 0.3676 |
| 7 | 34.5313 | 5.9722 | 3.0748 | 4.3703 | 2.1355 | 1.0432 | 11.8523 | 2.9206 |
| 8 | 33.5237 | 2.2277 | 8.2047 | 11.6614 | 3.8233 | 1.8677 | 1.8092 | 0.4458 |
| 9 | 32.5455 | 5.2515 | 1.7729 | 2.5198 | 10.2017 | 4.9835 | 3.2391 | 0.7982 |
| 10 | 31.5959 | 4.5986 | 3.6737 | 5.2215 | 2.2044 | 1.0769 | 8.6430 | 2.1298 |
| 11 | 30.6739 | 2.5334 | 6.0794 | 8.6407 | 4.5679 | 2.2314 | 1.8676 | 0.4602 |
| 12 | 29.7789 | 4.5322 | 1.9226 | 2.7326 | 7.5591 | 3.6926 | 3.8700 | 0.9536 |
| 13 | 28.9100 | 3.6923 | 3.7002 | 5.2591 | 2.3906 | 1.1678 | 6.4042 | 1.5781 |
| 14 | 28.0664 | 2.6146 | 4.6173 | 6.5626 | 4.6009 | 2.2475 | 2.0253 | 0.4991 |
| 15 | 27.2474 | 3.8603 | 2.0324 | 2.8887 | 5.7412 | 2.8045 | 3.8979 | 0.9605 |
| 16 | 26.4524 | 3.0721 | 3.4482 | 4.9010 | 2.5272 | 1.2345 | 4.8640 | 1.1985 |
| 17 | 25.6805 | 2.5471 | 3.6127 | 5.1348 | 4.2875 | 2.0944 | 2.1410 | 0.5276 |
| 18 | 24.9312 | 3.2745 | 2.0628 | 2.9319 | 4.4921 | 2.1944 | 3.6324 | 0.8951 |
| 19 | 24.2037 | 2.6248 | 3.0857 | 4.3857 | 2.5649 | 1.2530 | 3.8057 | 0.9378 |
| 20 | 23.4974 | 2.3914 | 2.9142 | 4.1419 | 3.8368 | 1.8742 | 2.1730 | 0.5355 |
| 21 | 22.8118 | 2.7811 | 2.0170 | 2.8668 | 3.6235 | 1.7701 | 3.2505 | 0.8010 |
| 22 | 22.1462 | 2.2825 | 2.7023 | 3.8408 | 2.5080 | 1.2251 | 3.0699 | 0.7565 |
| 23 | 21.5000 | 2.1910 | 2.4160 | 3.4339 | 3.3600 | 1.6414 | 2.1248 | 0.5236 |
| 24 | 20.8726 | 2.3718 | 1.9140 | 2.7203 | 3.0041 | 1.4675 | 2.8467 | 0.7015 |
| 25 | 20.2636 | 2.0054 | 2.3413 | 3.3278 | 2.3798 | 1.1625 | 2.5451 | 0.6271 |
| 26 | 19.6723 | 1.9748 | 2.0478 | 2.9105 | 2.9112 | 1.4221 | 2.0162 | 0.4968 |
| 27 | 19.0983 | 2.0337 | 1.7749 | 2.5227 | 2.5462 | 1.2438 | 2.4664 | 0.6078 |
| 28 | 18.5410 | 1.7712 | 2.0202 | 2.8713 | 2.2069 | 1.0781 | 2.1572 | 0.5316 |
| 29 | 18.0000 | 1.7606 | 1.7639 | 2.5070 | 2.5119 | 1.2271 | 1.8697 | 0.4607 |
| 30 | 17.4747 | 1.7534 | 1.6180 | 2.2997 | 2.1932 | 1.0714 | 2.1281 | 0.5244 |

**S1 Table S2.** Baffin Bay (BB) mortality rates (natural, total, and harvest) were calculated based on the natural and total survival rates reported in Peacock et al., 2012. The application of BB mortality rates to a RISKMAN stable-age distribution (S1 Table S1) suggested an inconsistency between the Peacock et al., 2012 estimated survival rates and the BB reported harvest (S1 Tables S3; S4 Tables S1a, S1b, and S1c). For comparison, the average BB harvest for the 2003-2009 period was ~215 bears (S1 Table S3), while the estimated annual harvest removal from Peacock et al., 2012 was only ~25 bears per year.

| **Age Class** | **Total Bears** | **Natural Mortality Rate** | **Natural Mortality** | **Total  Mortality Rate** | **Total Mortality** | **Harvest Mortality Rate** | **Harvest Mortality** |
| --- | --- | --- | --- | --- | --- | --- | --- |
| Juvenile Male | 262.9043 | 0.1170 | 30.7598 | 0.1440 | 37.8582 | 0.0270 | 7.0984 |
| Juvenile Female | 270.9939 | 0.1290 | 34.9582 | 0.1410 | 38.2101 | 0.0120 | 3.2519 |
| Subadult Male | 159.5669 | 0.2410 | 38.4556 | 0.2980 | 47.5509 | 0.0570 | 9.0953 |
| Subadult Female | 160.9340 | 0.2680 | 43.1303 | 0.2940 | 47.3146 | 0.0260 | 4.1843 |
| Adult Male | 674.2259 | 0.3340 | 225.1915 | 0.3350 | 225.8657 | 0.0010 | 0.6742 |
| Adult Female | 545.3742 | 0.3300 | 179.9735 | 0.3310 | 180.5189 | 0.0010 | 0.5454 |
| **Total** | 2073.9990 | N/A | 552.4689 | N/A | 577.3184 | N/A | 24.8495 |

**S1 Table S3.** Baffin Bay (BB) marked/unmarked bears by jurisdiction (Nunavut and Greenland) for the 1998-2009 period. BB harvest statistics were taken from the PBTC harvest records and PBSG status reports (Lunn et al., 2002; Aars et al., 2006; Obbard et al., 2010).

| **Year** | **Nunavut Marked** | **Nunavut Unmarked** | **Nunavut Total** | **Greenland Marked** | **Greenland Unmarked** | **Greenland Total** |
| --- | --- | --- | --- | --- | --- | --- |
| 1998 | 14 | 83 | 97 | 7 | 89 | 96 |
| 1999 | 5 | 59 | 64 | 14 | 83 | 97 |
| 2000 | 7 | 44 | 51 | 4 | 64 | 68 |
| 2001 | 8 | 54 | 62 | 2 | 95 | 97 |
| 2002 | 5 | 58 | 63 | 3 | 115 | 118 |
| 2003 | 5 | 56 | 61 | 6 | 200 | 206 |
| 2004 | 3 | 69 | 72 | 5 | 159 | 164 |
| 2005 | 7 | 90 | 97 | 2 | 153 | 155 |
| 2006 | 6 | 92 | 98 | 2 | 133 | 135 |
| 2007 | 8 | 91 | 99 | 4 | 75 | 79 |
| 2008 | 6 | 93 | 99 | 3 | 63 | 66 |
| 2009 | 6 | 97 | 103 | 7 | 66 | 73 |

**S1 Table S4.** A Fisher’s Exact Test comparison of Nunavut versus Greenland Baffin Bay recoveries for the 1998-2000, 1998-2001, 1998-2002, and 2003-2009 time bins. The results suggested that recoveries were not drawn from the same subpopulation for the intervals 1998-2002 (p< 0.0103) and 1998-2001 p< 0.0424; but were drawn from the same subpopulation for the 1998-2000 interval (p<0.3579, power=(1-β)=0.67).

| 1998-2000: p<0.3579, power=(1-β)=0.67 | | |
| --- | --- | --- |
| **Jurisdiction** | Marked | Unmarked |
| Nunavut | 26 | 186 |
| Greenland | 25 | 236 |
| 1998-2001: p<0.0424 | | |
| **Jurisdiction** | Marked | Unmarked |
| Nunavut | 34 | 240 |
| Greenland | 27 | 331 |
| 1998-2002: p<0.0103 | | |
| **Jurisdiction** | Marked | Unmarked |
| Nunavut | 39 | 298 |
| Greenland | 30 | 446 |
| 2003-2009 p<0.0041 | | |
| **Jurisdiction** | Marked | Unmarked |
| Nunavut | 41 | 588 |
| Greenland | 29 | 849 |

**Supplementary 2.** Canadian Polar Bear Subpopulation Survival Rates.

Age specific survival rates vary among subpopulations. Polar bear survival rates have been estimated using the following age strata definitions: cubs-of-the-year (COYs), yearlings and subadults (ages 1–4), prime-age adults (ages 5–20), and senescent adults (ages 21-30); except for (Western Hudson Bay) where Regehr et al. (2007a, 2007b) defined prime-age adults as ages 5-19 and senescent adults as ages 20-30. Survival rates by age strata class for each Canadian subpopulation are provided in S2 Tables S1 and S2. Total survival rates (S2 Table S1) include harvest mortality and are calculated without correction for harvest levels in some estimates. Natural survival rates (S1 Table S2) are corrected for harvest mortality and are the values used to determine the effect of a given harvest rate on subpopulation growth rate in a PVA simulation.

**S2 Table S1.** Mean (standard error [SE] in parentheses) of total (i.e., harvested) annual survival rates for age and sex classes of subpopulations of Canadian polar bears.

|  | **Males** | | | | | **Females** | | | | |
| --- | --- | --- | --- | --- | --- | --- | --- | --- | --- | --- |
| **Subpopulation** | **Total Survival / SE** | | | | | **Total Survival / SE** | | | | |
| **(primary data source)** | **0** | **1** | **2 - 4** | **5 – 20** | **>20** | **0** | **1** | **2 - 4** | **5 - 20** | **>20** |
| Baffin Bay  (Taylor et al., 2005) | 0.538 | 0.879 | 0.879 | 0.923 | 0.874 | 0.600 | 0.901 | 0.901 | 0.940 | 0.913 |
|  | (0.094) | (0.049) | (0.049) | (0.024) | (0.062) | (0.096) | (0.045) | (0.045) | (0.021) | (0.047) |
| Davis Strait (Peacock et al., 2013)^1^ | 0.538 | 0.879 | 0.879 | 0.923 | 0.874 | 0.600 | 0.901 | 0.901 | 0.940 | 0.913 |
|  | (0.094) | (0.049) | (0.049) | (0.024) | (0.062) | (0.096) | (0.045) | (0.045) | (0.021) | (0.047) |
| Foxe Basin^2^ | N/A | N/A | N/A | N/A | N/A | N/A | N/A | N/A | N/A | N/A |
|  | N/A | N/A | N/A | N/A | N/A | N/A | N/A | N/A | N/A | N/A |
| Gulf of Boothia  (Taylor et al., 2008c) | 0.8889 | 0.883 | 0.883 | 0.917 | 0.917 | 0.889 | 0.883 | 0.883 | 0.919 | 0.919 |
|  | (0.179) | (0.087) | (0.087) | (0.041) | (0.041) | (0.179) | (0.087) | (0.087) | (0.044) | (0.044) |
| Kane Basin  (Taylor et al., 2008a) | 0.308 | 0.617 | 0.617 | 0.957 | 0.957 | 0.374 | 0.686 | 0.686 | 0.967 | 0.967 |
|  | (0.172) | (0.180) | (0.180) | (0.046) | (0.046) | (0.180) | (0.157) | (0.157) | (0.043) | (0.043) |
| Lancaster Sound  (Taylor et al., 2008b)^3^ | 0.633 | 0.790 | 0.790 | 0.892 | 0.653 | 0.749 | 0.879 | 0.879 | 0.936 | 0.758 |
|  | (0.123) | (0.073) | (0.073) | (0.030) | (0.085) | (0.105) | (0.050) | (0.050) | (0.019) | (0.054) |
| M’Clintock Channel  (Taylor et al., 2006a) | 0.620 | 0.900 | 0.900 | 0.880 | 0.880 | 0.620 | 0.900 | 0.900 | 0.900 | 0.900 |
|  | (0.150) | (0.040) | (0.040) | (0.040) | (0.040) | (0.150) | (0.040) | (0.040) | (0.040) | (0.040) |
| Northern Beaufort Sea  (Stirling et al., 2011)^4^ | 0.516 | 0.328 | 0.823 | 0.825 | 0.401 | 0.537 | 0.333 | 0.905 | 0.906 | 0.575 |
|  | (0.349) | (0.311) | (0.148) | (0.145) | (0.304) | (0.285) | (0.314) | (0.094) | (0.092) | (0.283) |
| Norwegian Bay  (Taylor et al., 2008b)^3^ | 0.633 | 0.790 | 0.790 | 0.892 | 0.653 | 0.749 | 0.879 | 0.879 | 0.936 | 0.758 |
|  | (0.123) | (0.073) | (0.073) | (0.030) | (0.085) | (0.105) | (0.050) | (0.050) | (0.019) | (0.054) |
| Southern Beaufort Sea  (Regehr et al., 2006; 2010) | 0.430 | 0.920 | 0.920 | 0.920 | 0.920 | 0.430 | 0.920 | 0.920 | 0.920 | 0.920 |
|  | (0.110) | (0.040) | (0.040) | (0.040) | (0.040) | (0.110) | (0.040) | (0.040) | (0.040) | (0.040) |
| Southern Hudson Bay  (Obbard et al., 2007)^5^ | 0.492 | 0.644 | 0.811 | 0.811 | 0.293 | 0.485 | 0.645 | 0.892 | 0.892 | 0.444 |
|  | (0.141) | (0.141) | (0.075) | (0.075) | (0.132) | (0.133) | (0.133) | (0.051) | (0.051) | (0.146) |
| Viscount Melville Sound  (Taylor et al., 2002) | 0.448 | 0.774 | 0.774 | 0.774 | 0.774 | 0.693 | 0.905 | 0.905 | 0.905 | 0.905 |
|  | (0.216) | (0.081) | (0.081) | (0.081) | (0.081) | (0.183) | (0.026) | (0.026) | (0.026) | (0.026) |
| Western Hudson Bay  (Regehr et al., 2007a, 2007b)^6,7^ | 0.620 | 0.620 | 0.810 | 0.900 | 0.750 | 0.700 | 0.700 | 0.860 | 0.930 | 0.810 |
|  | (0.020) | (0.020) | (0.015) | (0.005) | (0.020) | (0.020) | (0.020) | (0.015) | (0.005) | (0.015) |

^1^ Davis Strait uses Baffin Bay total survival values due to meta-analysis. See comments.

^2^ No survival rates are available for Foxe Basin.

^3^ Survival rates pooled for Lancaster Sound and Norwegian Bay (see Taylor et al., 2008b).

^4^ 2003–2005 means. Estimated SE is the difference between the mean estimate and mean upper confidence limit, divided by 1.96.

^5^ 2003-2004 means. Estimated SE is the confidence interval, divided by 3.92. Results may differ from COSEWIC (2008) due to errors found in correspondence with Obbard et al. (2007).

^6^ Regehr et al. (2007a, 2007b) present total apparent survival rates for Western Hudson Bay polar bears as 95% CI. Estimated SE is the difference between the estimate and upper CL, divided by 1.96. Survival rates presented for 2-4 and 20+ adults are those that are not reduced from capture events around Churchill (see Regehr et al. [2007a, 2007b]). Survival rates for 2-4 and ≥20 age categories in Western Hudson Bay may be as low as 0.72 and 0.65 for males and 0.78 and 0.72 for females, respectively. The true survival rates for subadult and senescent bears in Western Hudson Bay likely lie somewhere between the rates in the table and those stated in the previous sentence (pers. comm..Regehr, E. 2007; COSEWIC, 2008).

^7^ Age strata classes from Regehr et al. (2007a, 2007b) differ from the other papers, adhering to the following groupings: 0-1, 2-4, 5-19, and 20-30.

**S2 Table S2.** Mean (standard error [SE] in parentheses) of natural (i.e., unharvested) annual survival rates for age and sex classes of subpopulations of Canadian polar bears.

|  | **Males** | | | | | **Females** | | | | |
| --- | --- | --- | --- | --- | --- | --- | --- | --- | --- | --- |
| **Subpopulation** | **Natural Survival / SE** | | | | | **Natural Survival / SE** | | | | |
| **(primary data source)** | **0** | **1** | **2 - 4** | **5 – 20** | **>20** | **0** | **1** | **2 - 4** | **5 - 20** | **>20** |
| Baffin Bay  (Taylor et al., 2005) | 0.570 | 0.938 | 0.938 | 0.947 | 0.887 | 0.620 | 0.938 | 0.938 | 0.953 | 0.919 |
|  | (0.094) | (0.045) | (0.045) | (0.022) | (0.060) | (0.095) | (0.042) | (0.042) | (0.020) | (0.046) |
| Davis Strait  (Peacock et al., 2013) | 0.916 | 0.934 | 0.923 | 0.955 | 0.897 | 0.916 | 0.934 | 0.931 | 0.962 | 0.911 |
|  | (0.057) | (0.032) | (0.034) | (0.020) | (0.073) | (0.057) | (0.032) | (0.033) | (0.019) | (0.070) |
| Foxe Basin (Taylor et al., 2005)^1^ | 0.570 | 0.938 | 0.938 | 0.947 | 0.887 | 0.620 | 0.938 | 0.938 | 0.953 | 0.919 |
|  | (0.094) | (0.045) | (0.045) | (0.022) | (0.060) | (0.095) | (0.042) | (0.042) | (0.020) | (0.046) |
| Gulf of Boothia  (Taylor et al., 2009) | 0.889 | 0.897 | 0.897 | 0.955 | 0.955 | 0.889 | 0.897 | 0.897 | 0.955 | 0.955 |
|  | (0.179) | (0.078) | (0.078) | (0.036) | (0.036) | (0.179) | (0.078) | (0.078) | (0.035) | (0.035) |
| Kane Basin  (Taylor et al., 2008a) | 0.345 | 0.663 | 0.663 | 0.997 | 0.997 | 0.410 | 0.756 | 0.756 | 0.997 | 0.997 |
|  | (0.200) | (0.197) | (0.197) | (0.026) | (0.026) | (0.200) | (0.159) | (0.159) | (0.026) | (0.026) |
| Lancaster Sound  (Taylor et al., 2008b)^3^ | 0.634 | 0.838 | 0.838 | 0.974 | 0.715 | 0.750 | 0.898 | 0.898 | 0.946 | 0.771 |
|  | (0.123) | (0.075) | (0.075) | (0.030) | (0.095) | (0.104) | (0.005) | (0.005) | (0.018) | (0.054) |
| M’Clintock Channel  (Taylor et al., 2006a) | 0.620 | 0.983 | 0.983 | 0.977 | 0.977 | 0.619 | 0.983 | 0.983 | 0.921 | 0.921 |
|  | (0.150) | (0.034) | (0.034) | (0.033) | (0.033) | (0.151) | (0.034) | (0.034) | (0.046) | (0.046) |
| Northern Beaufort Sea  (Stirling et al., 2011)^4^ | 0.457 | 0.930 | 0.892 | 0.872 | 0.676 | 0.443 | 0.930 | 0.958 | 0.932 | 0.584 |
|  | (0.481) | (0.040) | (0.156) | (0.152) | (0.351) | (0.344) | (0.040) | (0.098) | (0.094) | (0.323) |
| Norwegian Bay  (Taylor et al., 2008b)^3^ | 0.634 | 0.838 | 0.838 | 0.974 | 0.715 | 0.750 | 0.898 | 0.898 | 0.946 | 0.771 |
|  | (0.123) | (0.075) | (0.075) | (0.030) | (0.095) | (0.104) | (0.005) | (0.005) | (0.018) | (0.054) |
| Southern Beaufort Sea  (Regehr et al., 2006; 2010)^5^ | 0.430 | 0.930 | 0.930 | 0.930 | 0.930 | 0.430 | 0.930 | 0.930 | 0.930 | 0.930 |
|  | (0.11) | (0.040) | (0.040) | (0.040) | (0.040) | (0.11) | (0.040) | (0.040) | (0.040) | (0.040) |
| Southern Hudson Bay  (Obbard et al., 2007) ^6^ | 0.492 | 0.672 | 0.928 | 0.892 | 0.556 | 0.485 | 0.650 | 0.972 | 0.951 | 0.523 |
|  | (0.141) | (0.141) | (0.075) | (0.075) | (0.132) | (0.133) | (0.133) | (0.051) | (0.051) | (0.146) |
| Viscount Melville Sound  (Taylor et al., 2002) | 0.448 | 0.924 | 0.924 | 0.924 | 0.924 | 0.693 | 0.957 | 0.957 | 0.957 | 0.957 |
|  | (0.216) | (0.109) | (0.109) | (0.109) | (0.109) | (0.183) | (0.028) | (0.028) | (0.028) | (0.028) |
| Western Hudson Bay  (Regehr et al., 2007a, 2007b)^7,8^ | 0.710 | 0.710 | 0.940 | 0.940 | 0.820 | 0.730 | 0.730 | 0.930 | 0.930 | 0.820 |
|  | (0.020) | (0.020) | (0.015) | (0.005) | (0.020) | (0.020) | (0.020) | (0.015) | (0.005) | (0.015) |

^1^ No survival rates are available for Foxe Basin. Natural survival rates from a nearby subpopulation (BB) have been substituted in order to perform the simulations.

^3^ Survival estimates pooled for Lancaster Sound and Norwegian Bay (see Taylor et al., 2008b).

^4^ Natural survival estimates for NB were estimated by: (1) subtracting the difference between NB total survival and NB natural survival from the 2008 COSEWIC status report (COSEWIC, 2008), then (2) subtracting this difference from the NB total survival estimates in Stirling et al. (2011).

^5^ Natural survival estimates for SB were estimated by: (1) subtracting the difference between SB total survival and SB natural survival from the 2008 COSEWIC status report (COSEWIC, 2008), then (2) subtracting this difference from the SB total survival estimates in Regehr et al. (2006).

^6^ Natural survival estimates for SH were estimated by: (1) subtracting the difference between SH total survival and SH natural survival from the 2008 COSEWIC status report, then (2) subtracting this difference from the SH 2003-2004 pooled total survival estimates in Obbard et al. (2007).

^7^ Natural survival rates provided in Regehr et al. (2007a, 2007b) include two estimates for the 2-4 and 20+ age categories. One estimate is for the mark-recapture model that excludes a capture effect on mortality of handling bears in Churchill; the other estimate is rates that are reduced to reflect heterogeneity in the data associated with captures around Churchill by the Manitoba Department of Conservation. We used the former of these two estimates. Regehr et al. (2007a, 2007b) present no error estimates with these rates; for simulations the errors associated with total survival rates were used.

^8^ Age classifications from Regehr et al. (2007a, 2007b) differ from the other papers, adhering to the following groupings: 0-1, 2-4, 5-19, and 20-30.

**Supplementary 3.** Canadian Polar Bear Recruitment Rates.

The three year reproduction cycle of polar bears requires a different parameterization than the usual annual birth-pulse lifetable m_x_ values. The time of census is the same as for survival estimates (S2). The mean and standard error of the proportion of males in a litter, litter size (includes males and females), and age-specific probabilities of litter production for available females are provided in S3 Table S1. Available females are females with no cubs or females with 2-year olds. Encumbered females with cubs of the year (COYs) or yearlings do not engage in breeding and do not produce litters the following year.

**S3 Table S1.** Estimated means (and standard errors [SE] in parentheses) of post-den emergence litter size and age-specific probabilities of litter production (LPR) for lone females or females with dispersing (2-year-old) cubs (because of the 3-year reproductive cycle of polar bears, females with cubs-of-the-year or yearlings are not available to mate and are not included in the LPR computation).

| **Subpopulation (primary data source)** | **Cub (age 0) litter size/  SE** | **Age 4 LPR/ SE** | **Age 5 LPR/ SE** | **Age 6 LPR/ SE** | **Age 7+ LPR/ SE** | **Proportion of Males at Birth/  SE** |
| --- | --- | --- | --- | --- | --- | --- |
| Baffin Bay (Taylor et al., 2005) | 1.587 | 0.096 | 0.880 | 1.000 | 1.000 | 0.493 |
|  | (0.073) | (0.120) | (0.400) | (0.167) | (0.167) | (0.029) |
| Davis Strait (Peacock et al., 2013)^1^ | 1.487 | 0.069 | 0.543 | 0.338 | 0.441 | 0.550 |
|  | (0.140) | (0.064) | (0.203) | (0.976) | (0.107) | (0.040) |
| Foxe Basin (Taylor et al., 2005)^2^ | 1.587 | 0.096 | 0.880 | 0.850^3^ | 0.850^3^ | 0.493 |
|  | (0.073) | (0.120) | (0.400) | (0.167) | (0.167) | (0.029) |
| Gulf of Boothia (Taylor et al., 2008c) | 1.648 | 0.000 | 0.194 | 0.467 | 0.970 | 0.460 |
|  | (0.098) | (0) | (0.178) | (0.168) | (0.300) | (0.090) |
| Kane Basin (Taylor et al., 2008a) | 1.667 | 0.000 | 0.000 | 0.357 | 0.978 | 0.426 |
|  | (0.083) | (0) | (0) | (0.731) | (0.083) | (0.029) |
| Lancaster Sound (Taylor et al., 2008b) | 1.688 | 0.000 | 0.107 | 0.312 | 0.954 | 0.531 |
|  | (0.012) | (0) | (0.050) | (0.210) | (0.083) | (0.048) |
| M'Clintock Channel (Taylor et al., 2006a)^4^ | 1.680 | 0.000 | 0.11 | 0.29 | 0.93 | 0.550 |
|  | (0.147) | (0) | (0.11) | (0.47) | (0.33) | (0.060) |
| Northern Beaufort Sea (PBTC, 2007) | 1.756 | 0.118 | 0.283 | 0.883 | 0.883 | 0.502 |
|  | (0.166) | (0.183) | (0.515) | (0.622) | (0.622) | (0.035) |
| Norwegian Bay (Taylor et al., 2008b) | 1.714 | 0.000 | 0.000 | 0.000 | 0.689 | 0.544 |
|  | (0.080) | (0) | (0) | (0) | (0.053) | (0.066) |
| Southern Beaufort Sea (Regehr et al., 2006)^5^ | 1.750 | 0.000 | 0.470 | 0.470 | 0.470 | 0.520 |
|  | (0.170) | (0) | (0.090) | (0.090) | (0.090) | (0.040) |
| Southern Hudson Bay (PBTC, 2007)^6^ | 1.575 | 0.087 | 0.966 | 0.967 | 0.967 | 0.467 |
|  | (0.116) | (0.202) | (0.821) | (0.022) | (0.022) | (0.086) |
| Viscount Melville Sound (Taylor et al., 2002) | 1.640 | 0.000 | 0.623 | 0.872 | 0.872 | 0.535 |
|  | (0.125) | (0) | (0.414) | (0.712) | (0.712) | (0.118) |
| Western Hudson Bay (Aars et al., 2006; PBTC, 2007)^7^ | 1.540 | 0.000 | 0.257 | 0.790 | 0.790 | 0.480 |
|  | (0.110) | (0) | (0.442) | (0.180) | (0.180) | (0.110) |

^1^ DS reproductive rates were also provided through correspondence with Dr. Lily Peacock.

^2^ No reproductive rates are available for Foxe Basin. Reproductive rates from a nearby subpopulation (BB) have been substituted in order to perform the simulations.

^3^ Baffin Bay adult litter production rates were reduced from 1.0 to 0.85 to produce a simulation that resulted in ~2700 individuals in 2010 (to match the Stapleton et al. (2016) aerial survey estimate), and used these empirically corrected Baffin Bay demographic rates for the Foxe Basin PVA.

^4^ Results may differ from COSEWIC (2008) due to errors found in correspondence with Taylor et al. (2006a).

^5^ No mean LPR for an age category is presented in Regehr et al. (2007a, 2007b). Selected values provided by E. Regehr (USGS, Alaska Science Centre, Anchorage, AK) for the 2007 meeting of the PBTC.

^6^ Also presented in Aars et al. (2006).

^7^ Data presented in Table 3 of Aars et al. (2006), updated online version only.

**Supplementary 4.** Total Human-Caused Mortality Rates for Canadian Polar Bear Subpopulations.

Total anthropogenic mortalities (harvest, defense, accidental, and illegal) are monitored and reported for all subpopulations within or shared by Canada. The total anthropogenic mortality and the sex and age distribution of the harvest are available by harvest season for each Canadian polar bear subpopulation from the year of most recent estimate of abundance to the 2011/2012 harvest season. These are provided in S4 Tables S1**a**, S1**b**, and S1**c** (updated from York, 2012 Tables 4.5 and 4.6).

**S4 Table S1a.** Total anthropogenic (harvest, defense, accidental, and illegal) mortality rates (Kill) and the proportion that were females (Prop F) for each Canadian subpopulation are summarized by harvest season for the 1993/1994 to 1999/2000 interval (York, 2012; PBTC, 2013). Anthropogenic mortality rates are presented starting from the last published subpopulation estimate to the present; intervals relevant to our pre-2013 PVA simulations (see Methods).

| Season | *93/94* | | *94/95* | | *95/96* | | *96/97* | | *97/98* | | *98/99* | | *99/00* | |
| --- | --- | --- | --- | --- | --- | --- | --- | --- | --- | --- | --- | --- | --- | --- |
| Subpop. | *Kill* | *Prop F* | *Kill* | *Prop F* | *Kill* | *Prop F* | *Kill* | *Prop F* | *Kill* | *Prop F* | *Kill* | *Prop F* | *Kill* | *Prop F* |
| Baffin Bay |  |  |  |  |  |  |  |  | 193 | 0.31 | 161 | 0.37 | 119 | 0.34 |
| Davis Strait |  |  |  |  |  |  |  |  |  |  |  |  |  |  |
| Foxe Basin | 100 | 0.485 | 118 | 0.31 | 95 | 0.35 | 97 | 0.33 | 76 | 0.49 | 82 | 0.24 | 95 | 0.37 |
| Gulf of Boothia |  |  |  |  |  |  |  |  |  |  |  |  | 33 | 0.39 |
| Kane Basin |  |  |  |  |  |  |  |  | 11 | 0.27 | 11 | 0.27 | 10 | 0.3 |
| Lancaster Sound |  |  |  |  |  |  |  |  | 76 | 0.28 | 74 | 0.20 | 75 | 0.25 |
| M'Clintock Channel |  |  |  |  |  |  |  |  |  |  |  |  | 22 | 0.27 |
| Northern Beaufort Sea |  |  |  |  |  |  |  |  |  |  |  |  |  |  |
| Norwegian Bay |  |  |  |  |  |  |  |  | 3 | 0.33 | 4 | 0.25 | 4 | 0.25 |
| Southern Beaufort Sea |  |  |  |  |  |  |  |  |  |  |  |  |  |  |
| Southern Hudson Bay |  |  |  |  |  |  |  |  |  |  |  |  |  |  |
| Viscount Melville Sound |  |  |  |  |  |  |  |  |  |  | 0 | 0 | 4 | 0.25 |
| Western Hudson Bay |  |  |  |  |  |  |  |  |  |  |  |  |  |  |

**S4 Table S1b.** Total anthropogenic (harvest, defense, accidental, and illegal) mortality rates (Kill) and the proportion that were females (Prop F) for each Canadian subpopulation are summarized by harvest season for the 2000/2001 to 2006/2007 interval (York, 2012; PBTC, 2013). Anthropogenic mortality rates are presented starting from the last published subpopulation estimate to the present; intervals relevant to our pre-2013 PVA simulations (see Methods).

| Season | *00/01* | | *01/02* | | *02/03* | | *03/04* | | *04/05* | | *05/06* | | *06/07* | |
| --- | --- | --- | --- | --- | --- | --- | --- | --- | --- | --- | --- | --- | --- | --- |
| Subpop. | *Kill* | *Prop F* | *Kill* | *Prop F* | *Kill* | *Prop F* | *Kill* | *Prop F* | *Kill* | *Prop F* | *Kill* | *Prop F* | *Kill* | *Prop F* |
| Baffin Bay | 159 | 0.43 | 181 | 0.29 | 267 | 0.33 | 236 | 0.33 | 252 | 0.35 | 233 | 0.32 | 178 | 0.35 |
| Davis Strait |  |  |  |  |  |  |  |  |  |  |  |  | 58 | 0.24 |
| Foxe Basin | 99 | 0.27 | 98 | 0.35 | 96 | 0.38 | 95 | 0.26 | 94 | 0.27 | 103 | 0.38 | 102 | 0.41 |
| Gulf of Boothia | 41 | 0.34 | 43 | 0.32 | 38 | 0.47 | 41 | 0.39 | 66 | 0.32 | 65 | 0.31 | 72 | 0.36 |
| Kane Basin | 11 | 0.36 | 10 | 0.3 | 10 | 0.3 | 8 | 0.38 | 11 | 0.36 | 25 | 0.2 | 9 | 0.22 |
| Lancaster Sound | 62 | 0.16 | 71 | 0.25 | 71 | 0.21 | 79 | 0.28 | 87 | 0.23 | 81 | 0.22 | 94 | 0.21 |
| M'Clintock Channel | 12 | 0.33 | 0 | 0 | 1 | 0 | 0 | 0 | 2 | 0.5 | 3 | 0 | 3 | 0.00 |
| Northern Beaufort Sea |  |  |  |  |  |  |  |  |  |  |  |  | 31 | 0.35 |
| Norwegian Bay | 4 | 0.25 | 1 | 0 | 1 | 0 | 3 | 0.33 | 4 | 0 | 3 | 0 | 4 | 0.00 |
| Southern Beaufort Sea |  |  |  |  |  |  |  |  |  |  |  |  | 38 | 0.34 |
| Southern Hudson Bay |  |  |  |  |  |  |  |  |  |  | 38 | 0.36 | 38 | 0.28 |
| Viscount Melville Sound | 4 | 0.5 | 4 | 0 | 4 | 0 | 5 | 0.2 | 5 | 0.4 | 4 | 0.25 | 6 | 0.33 |
| Western Hudson Bay |  |  |  |  |  |  |  |  | 43 | 0.30 | 37 | 0.44 | 58 | 0.31 |

**S4 Table S1c.** Total anthropogenic (harvest, defense, accidental, and illegal) mortality rates (Kill) and the proportion that were females (Prop F) for each Canadian subpopulation are summarized by harvest season for the 2007/2008 to the 2011/2012 interval (York, 2012; PBTC, 2013). Anthropogenic mortality rates are presented starting from the last published subpopulation estimate to the present; intervals relevant to our pre-2013 PVA simulations (see Methods). Five-year mean human-caused mortality rates are also presented for the post-2013 PVA simulations (see Methods).

| Season | *07/08* | | *08/09* | | *09/10* | | *10/11* | | *11/12* | | 5-year mean | |
| --- | --- | --- | --- | --- | --- | --- | --- | --- | --- | --- | --- | --- |
| *Subpopulation* | *Kill* | *Prop F* | *Kill* | *Prop F* | *Kill* | *Prop F* | *Kill* | *Prop F* | *Kill* | *Prop F* | *Kill* | *Prop F* |
| Baffin Bay | 165 | 0.448 | 176 | 0.341 | 155 | 0.303 | 163 | 0.337 | 161 | 0.354 | 164.0 | 0.357 |
| Davis Strait | 68 | 0.235 | 76 | 0.368 | 69 | 0.377 | 86 | 0.395 | 107 | 0.402 | 81.2 | 0.356 |
| Foxe Basin | 107 | 0.626 | 109 | 0.284 | 109 | 0.367 | 107 | 0.374 | 112 | 0.366 | 108.8 | 0.403 |
| Gulf of Boothia | 56 | 0.411 | 72 | 0.403 | 57 | 0.439 | 45 | 0.400 | 69 | 0.275 | 59.8 | 0.385 |
| Kane Basin | 5 | 0.800 | 7 | 0.143 | 3 | 0.667 | 5 | 0.400 | 5 | 0.4 | 5.0 | 0.482 |
| Lancaster Sound | 74 | 0.257 | 94 | 0.383 | 73 | 0.370 | 84 | 0.238 | 98 | 0.306 | 84.6 | 0.311 |
| M'Clintock Channel | 3 | 0.333 | 2 | 0.000 | 3 | 0.000 | 3 | 0.333 | 3 | 0.333 | 2.8 | 0.200 |
| Northern Beaufort Sea | 18 | 0.389 | 34 | 0.471 | 13 | 0.538 | 45 | 0.311 | 52 | 0.346 | 32.4 | 0.411 |
| Norwegian Bay | 4 | 0.000 | 0 | 0.000 | 1 | 0.000 | 3 | 0.000 | 1 | 0 | 1.8 | 0.000 |
| Southern Beaufort Sea | 28 | 0.429 | 31 | 0.032 | 24 | 0.500 | 51 | 0.412 | 50 | 0.28 | 36.8 | 0.331 |
| Southern Hudson Bay | 34 | 0.294 | 37 | 0.351 | 62 | 0.387 | 104 | 0.346 | 49 | 0.265 | 57.2 | 0.329 |
| Viscount Melville Sound | 3 | 0.000 | 5 | 0.000 | 3 | 0.000 | 7 | 0.429 | 4 | 0.5 | 4.4 | 0.186 |
| Western Hudson Bay | 32 | 0.438 | 14 | 0.357 | 18 | 0.167 | 15 | 0.400 | 29 | 0.31 | 21.6 | 0.334 |

**Supplementary 5.** Effect of Truncated Iterations on Monte Carlo Estimates of Subpopulation Growth Rate (λ).

The simulation protocol for occasions when the initial subpopulation random deviate was ≤ 0, or the harvest could not be satisfied by the subpopulation, or all individuals remaining in the subpopulation did not survive (individual based model) was to set the simulation N_t_ = 0 for the remaining years (t). Subsequent estimates of subpopulation growth rate (λ) were undefined because the denominator of N_t+1_/N_t_ was zero, so post-truncation λ was set to zero. This protocol caused Monte Carlo mean N_t_ to be over-estimated (positive bias) because the upper values were not bounded, but the lower values were bounded at 0 (S5 Fig. S1). Additionally, direct Monte Carlo estimates (mean of iteration values) of both arithmetic and geometric λ^t^ were biased (under-estimated) by either including truncated runs as λ=0 (S5 Table S1; S5 Fig. S2). The effect of a constant harvest (quota) accelerates when the subpopulation declines and decelerates when the subpopulation increases. The abrupt decline to λ=0 when the harvest cannot be satisfied or the projected number is less than 0 has a large negative effect on Monte Carlo mean λ_t_ relative to simulation with no truncations. The estimate of mean λ^t^ as N_t_/N_0_ also had a positive bias because of the positive bias in N_t_ estimates from truncated runs mentioned above.

The Monte Carlo estimate of N_t_ could be argued to be unbiased when truncations occur because subpopulations cannot be less than 0. However, in a PVA context, estimates of λ and N_t_ are viewed as summary parameters of a given survival, recruitment, and harvest schedule. PVA implicitly assumes that these summary parameters estimate not only subpopulation performance over some interval of time, but also provide an expectation of future performance providing all things remain equal. Although an argument could be made that the arithmetic mean λ for the last time interval is the best indication of subpopulation performance for that interval, that estimate is the λ associated with the extant (non-truncated) runs only, which are by definition the most optimistic of the simulation set. The Monte Carlo geometric λ estimate and the “N-based” (mean N_t_/mean N_0_) estimate are only unbiased when there are no truncated Monte Carlo runs. For consistency (all subpopulations considered with the same criteria) we report subpopulation status only as the probability of decline from 2013 (Table 2b) because this metric is not affected by truncations.

**S5 Table S1.** The effect of including/excluding truncated runs in the calculations of geometric mean subpopulation growth rates (λ). Each subpopulation was simulated from a current estimate of abundance (2013) for a 20 year period, using natural survival estimates and the 2007-2012 mean annual removals (S4 Table S3). The “Monte Carlo” geometric λ estimate and the “N-Based” (mean N_t_/mean N_0_) estimate are provided for each subpopulation are provided for both scenarios.

|  | Truncated Runs Included | | Truncated Runs Excluded | |  |  |
| --- | --- | --- | --- | --- | --- | --- |
|  | Monte Carlo | N-based | Monte Carlo | N-based |  | |
| **Subpop.** | **λ** | **Λ** | **λ** | **λ** | **Probability of Decline** | **Proportion Of Truncated Runs** |
|  |  |  |  |  |  |  |
| Baffin Bay | 0.2594 | 1.0330 | 1.0312 | 1.1073 | 0.788 | 0.7484 |
| Davis Strait | 1.0001 | 1.0137 | 1.0058 | 1.0135 | 0.3894 | 0.0056 |
| Foxe Basin | 0.8464 | 1.0420 | 1.0322 | 1.0522 | 0.2892 | 0.180 |
| Gulf of Boothia | 0.9400 | 1.0694 | 1.0540 | 1.0766 | 0.2016 | 0.107 |
| Kane Basin | N/A | N/A | N/A | N/A | N/A | N/A |
| Lancaster Sound | 0.8815 | 1.0204 | 1.0141 | 1.0272 | 0.3632 | 0.1312 |
| M'Clintock Channel | 0.9700 | 1.0322 | 1.0168 | 1.0345 | 0.3178 | 0.0458 |
| Northern Beaufort Sea | 0.2707 | 0.9944 | 1.0104 | 1.0574 | 0.8348 | 0.7328 |
| Norwegian Bay | 0.9943 | 1.0097 | 1.0050 | 1.0105 | 0.4034 | 0.0106 |
| Southern Beaufort Sea | 0.4827 | 0.9564 | 0.9659 | 0.9886 | 0.889 | 0.5008 |
| Southern Hudson Bay | 0.0306 | 0.8904 | 1.0036 | 1.0554 | 0.9816 | 0.9696 |
| Viscount Melville Sound | 0.9412 | 1.0745 | 1.0599 | 1.0820 | 0.1884 | 0.1106 |
| Western Hudson Bay | 0.2395 | 0.8984 | 0.9469 | 0.9629 | 0.9954 | 0.747 |

**Supplementary 6.** Eight Instances Where TEK Identified a Polar Bear Subpopulation Trend or Biological Feature before Science Could Identify or Confirm It.

1. Cambridge and Gjoa Haven requested quota reductions in the 1993 polar bear M’Clintock Channel (MC) Memoranda of Understanding (MOU) (M. Taylor, pers. comm. 1986-2008) because they felt numbers were declining in the eastern portion of MC. Taloyoak hunts from the MC subpopulation core had not seen reductions in their hunting area; and did not request a quota reduction for their community.
2. The Foxe Basin (FB) elders advised (through Hunters and Trappers’ Organization reps) that FB numbers had declined, after caribou were lost from Southampton Island and polar bear hunting increased. The input from the elders allowed the 1993 FB MOUs completed, even though FB communities had their polar bear quotas reduced. The recent FB aerial survey confirms that the FB subpopulation did increase slowly (as planned) and that the quota increase identified in 2004 FB MOUs (by TEK) were sustainable. A comparison of the PVA trajectory and the aerial survey estimate for FB is displayed in Figure 8.
3. Grise Fiord was aware of unsustainable (and sometimes illegal) hunting by Greenlanders in Kane Basin (KB) and requested that NWT, Greenland, and Canada try to do something about it. The KB study in the mid-1990's (Taylor et al., 2009) confirmed their impressions.
4. Hunters in Davis Strait (DS) were aware of the subpopulation increase in DS well before the recent study was done. The DS subpopulation was reported as declining by the Polar Bear Specialist Group (PBSG) in their 2009 status report (Obbard et al., 2010), but the PBSG status report and subsequent status report that draw from it are in error (Peacock et al., 2013). The subpopulation was projected to increase at about 0.6 % at current harvest levels (Peacock et al., 2013), while we estimate a 38.94% probability of decline (Table 2b).
5. Gulf of Boothia (GB) communities advised in 1993 and 1996 that their subpopulation had increased and was being under-utilized. This was confirmed by the GB study (Taylor et al., 2009) that finished in 2000.
6. Inuvialuit advised that Viscount Melville Sound (VM) had been over-hunted, and supported (1993 VM MOUs) which specified a five-year moratorium to allow recovery then reduced harvesting to allow continued recovery afterwards.
7. Lancaster Sound (LS) communities agreed that historical quotas had been sustained, and did not report a subpopulation increase or request a quota increase. This perspective was supported by the LS inventory in the mid-1990's (Taylor et al., 2008b).
8. Inuit advised that Norwegian Bay (NW) bears were distinct from those in surrounding areas (Taylor et al., 2008b), and genetic analysis (Paetkau et al., 1999) confirmed that NW bears were the most distinct of any extant subpopulation of polar bears.

**Supplementary 7.** Evaluation of Global Temperature, Arctic temperature, Global Ocean Heat Content (0-700 m), Arctic Sea Ice Extent Trends, and the Effects of Climate Change on Canadian Polar Bears.

The Arctic is expected to warm more rapidly and to a greater extent than the rest of the globe (Manabe and Stouffer, 1980; Screen and Simmonds, 2010). During the late 1990s and the 2000s, climate warming has coincided with a decline in seasonal and perennial Arctic sea ice cover (Kwok et al., 2009). Several authors have recently suggested that climate warming with consequent sea ice reduction poses the most significant threat to polar bears as a species (Amstrup et al., 2007, 2008, 2010; Hunter et al., 2010; Stirling, 2011; Stirling & Derocher, 2012; Derocher et al., 2013). Management activities aimed towards other threats (e.g., overhunting) have been viewed as secondary, claiming that they are unlikely to make a difference in the prognosis for polar bears as a species in a warming climate due to increased greenhouse gas emissions (Amstrup et al., 2011). A number of recent papers on polar bear distribution, subpopulation status, nutrition, and even genetics assume explicitly that current General Circulation Models (GCM) can accurately forecast future climate (IPCC, 2007; IPCC, 2013), and thus allow reliable predictions about how climate impacts will affect polar bear subpopulations (Amstrup et al., 2007, 2008; Hunter et al., 2010; Obbard et al., 2010; Stirling, 2011; Stirling & Derocher, 2012; Derocher et al., 2013). The recent polar bear literature is replete with phrases stating that the Arctic is rapidly warming and sea ice is rapidly declining (e.g., “The sea ice habitat upon which polar bears depend for successful foraging is rapidly declining in response to greenhouse gas (GHG)-driven global warming”, Derocher et al., 2013). In considering the relevance of our demographic perspectives on polar bear subpopulations in this time of “rapid climate warming”, we looked empirically at the recent trends in global atmospheric temperature, Arctic atmospheric temperature, ocean heat content, and Arctic sea ice extent.

Global temperatures (HadCRUT4), upper ocean (0-700 meters) temperatures (National Oceanic Data Center), Arctic temperatures (Remote Sensing Systems, Inc.), and Arctic sea ice extent (National Snow and Ice Data Center) have been estimated and are available for the 1980-2013 period (S7 Tables S1 and S2); where reliable data is available for each of the attributes. We used break-point linear regression to investigate trends for each dataset. Null hypotheses of no trends or no correlations were rejected at *p* < 0.05. We also estimated the power (1-β) of the test using G-Power software (Faul et al., 2007).

Both the atmosphere and the oceans have warmed (IPCC, 2007, 2013; Levitus et al., 2012; S7 Tables S1, S2, and S3; S7 Fig. S1, S2, and S3) and Arctic sea ice has declined since satellite records began in 1978 (Parkinson et al., 1999; Comiso, 2008; S7 Fig. S4). The 1980-2013 trends in global atmospheric temperature (p≤ 0.001), Arctic atmospheric temperature (p≤ 0.001), and ocean heat content (p≤ 0.001) were all statistically significant. Global atmospheric temperatures have been increasing at a rate of approximately 0.16°C per decade since 1980 (S7 Table S3). Arctic atmospheric temperatures have also experienced a statistically significant (p≤ 0.001) long-term warming at a rate of 0.32°C per decade since 1980 (S7 Table S4). The 1980-2013 trend in ocean heat content indicates that the amount of heat stored in the upper ocean has increased at a rate of 0.445 10^22 Joules per decade (S7 Table S3; S7 Fig. S3). As expected during a period of climate warming, the trend in Arctic sea ice extent (as an annual average) for this interval is declining (0.55 10^6 km^2^ per year (p<0.001) (S7 Table S3). However, in recent years all of these trends are reduced except for upper ocean warming; and many are no longer significant over time spans longer than a decade (e.g., global atmospheric temperature trend has no significant trend since 1997 (p = 0.179) (S7 Table S3; S7 Fig. S1).

Sea ice has declined more in the warmer months than the colder months (Parkinson et al., 1999; S7 Tables S1, S2, and S4; S7 Fig. S5). Trends in sea ice extent for the warmer months (i.e., June-October) and the colder months (i.e., November-May) were all numerically negative and all statistically significant since 1980 (S7 Table S4). However, breakpoint regression from 2013 identified no significant trend in sea ice extent by month for periods ranging from 8 to 19 years (S7 Tables S5 and S6) with the November-June average no-trend length being 12.5 years and the July-September average no-tend length being 8.5 years (p<0.028). Failure to reject Ho: no trend could be because there is no trend, or because the power of the test was insufficient to detect an existing trend. Our protocol also examined the power of the test to detect a trend as large as or larger than the trend identified from least squares regression for the “no-trend” interval (S7 Table S5).

The correlation between annual sea ice extent and global temperature for the interval 1980-2013 was highly significant for all months of the year, while the same comparison for the 1997-2013 interval was not significant for any months of the year (S7 Tables S7 and S8; S7 Fig. S6). The lack of correlation could occur because the two measures were uncoupled, or because they were out of phase (lag effect).

Breakpoint regression of Arctic temperatures since 1980 (S7 Table S3; S7 Fig. S2) reveals that the interval from 1980 to 2013 can be divided into two intervals with no significant trend (1980-1998 and 1998-2013, with a highly significant difference of -0.616°C (p<0.001) between the mean temperatures of these two intervals (S7 Table S9). The perspective of climate change occurring as a series of shifting climate states rather than a single (constant) incremental long-term climate trends has also been identified for global atmospheric temperatures (Swanson and Tsonis, 2009) and ocean temperatures (Douglas & Knox, 2012).

Does the evidence for reduced warming rates in recent decades indicate that the rapid Arctic sea ice decline over the first decade of the 21^st^ century could be driven by factors other than CO_2_ driven global climate change? Or is this apparent “pause” in global surface warming, Arctic sea ice decline, and Arctic warming due to global surface temperatures being dampened by other climate factors such as heat sequestering in the oceans (e.g., Levitus et al., 2012; Meehl et al., 2011)? These questions are relevant to the long-term prognosis for polar bear subpopulations, but beyond the scope of this paper. The empirical evidence we have summarized indicates that warming has not “paused” because ocean temperatures have continued to increase while no trends were apparent in the recent global atmospheric mean annual temperature, Arctic mean annual temperature, and Arctic mean annual and monthly sea ice extent (S7 Table S3). We were surprised at the low power associated with the highly publicized no-trend findings for recent global atmospheric temperature. We contrast the relatively high power (β=0.933) associated with the 1980-1997 positive trend for global atmospheric temperature (17 years) with the low power (β=0.282) associated with the recent (1997-2013, 17 years), no-trend interval (S7 Table S3; S7 Fig. S1). Our interpretation for this time series would be no *detectable* trend for the recent interval because of the reduced slope and underlying environmental variance. We interpret these finding as evidence that global atmospheric warming has slowed, but the evidence is insufficient to conclude that atmospheric warming has stopped or paused. The same pattern is not evident for Arctic atmospheric warming. We see two distinct intervals (1980-1998 and 1998-2013), with the more recent interval being warmer by about 0.616°C (p<0.001) (S7 Table S3; S7 Fig. S2). As detailed above, recent Arctic sea ice trends appear to have two distinct seasonal strata; with July-Oct ice declining and no trend (approximately medium power, Cohen, 1988) for Nov-June sea ice.

Recent changes in Arctic sea ice were not predicted by GCMs, and we are not aware of a comprehensive explanation for this behaviour even in the context of trends in global atmospheric temperature, Arctic atmospheric temperature and upper (0-700 m) ocean warming. Combined with recent unexpected behaviour in Antarctic sea ice (Parkinson and Cavalieri, 2012) we suggest that the science is not “settled” with regards to the sea ice response to transient climate change. In this context, we question the certainty with which GCM projections have been used to predict polar bear response by 2050 (e.g., Amstrup et al., 2007) given the complex climate response observed just 6 years later. In our opinion, the use of GCM simulations of sea ice cover to predict polar bear subpopulation trend and persistence over the next several decades is essentially hypothetical.

Although GCM predictions have been steadily improving over the past two decades (Maslowski et al., 2012), discrepancies between observed trends and GCM forecasts of sea ice extent suggest that the treatment of sea ice in these models remains problematic. A comparison of individual GCM forecasts for sea ice and global temperature to current sea ice extent and current global temperature (Kirtman et al., 2013; S7 Fig. S7) shows that while global warming has been on the low end of GCM projections for this period, predictions of summer Arctic sea ice decline have been too conservative (Stroeve et al., 2007; S7 Fig. S8 and S9). One reason could be that ocean currents have caused greater heat transfer from the tropics to the poles than the 2007 GCMs anticipated (Stroeve et al., 2012). The oceans hold about 2100 times more heat than the atmosphere, and at least 70% of stored heat occurs in the upper 700 m of the oceans (Levitus et al., 2012). Current GCMs may have underestimated the contribution of ocean heat to convective Arctic sea ice reduction.

With respect to climate effects on polar bears, the lack of Arctic sea ice decline in the late fall to early summer months in the last 10 years demonstrates that seasonal Arctic sea ice regeneration in cold months can occur even as Arctic sea ice extent in warmer seasons declines (S7 Fig. S5); even when multiyear ice has been reduced (Maslanik et al., 2011). Polar bears are hyperphagic in the late spring and early summer (Stirling, 1998, 2011; Cherry et al., 2013) so this recent slowing of sea ice decline in these months may reduce the impact of sea ice in the “open-water” months in areas where sea ice did decline during the “open water” season.

While warming and sea ice decline have occurred throughout Arctic regions, the magnitude of these effects differs regionally (Amstrup et al., 2008; Thiemann et al., 2008). Even adjacent regions can be qualitatively different. For example, the Canadian Archipelago, where approximately one third of Canada’s polar bears reside (COSEWIC, 2008; Obbard et al., 2010; Table 2a), continues to have about the same multi-year and annual sea ice in all seasons (Sou & Flato, 2009), as opposed to the adjacent Arctic Basin region which annual sea ice has declined in the summer months and Arctic Basin sea ice mass has declined due to loss of both annual and multi-year ice (Maslanik et al., 2011). As a result, some researchers have hypothesized that the effects of climate warming and sea ice decline on polar bears will not be uniform across the Arctic based on differential climate and sea ice dynamics (Amstrup et al., 2008; Thiemann et al., 2008).

GCM modeling of Arctic sea ice tends to focus on the Arctic Basin beyond the Canadian Archipelago (Sou & Flato, 2009). This large basin of distinct land, sea and multiyear ice surfaces is easier to resolve (model) in GCMs. Polar bears use habitats differently depending on both physical and biological factors. Annual sea ice is preferred for hunting, but heavy multi-year sea ice is also used for denning in some areas (Lentfer, 1975; Amstrup & Gardner, 1994). Annual sea ice located over the continental shelf is preferred habitat for polar bears because of higher biological productivity compared to deep-water regions and greater accessibility to prey species (Derocher et al., 2004; Durner et al., 2004, 2009; Harwood et al., 2012). Over half of the world’s polar bear subpopulations and more than half of the world’s polar bears occur within or adjacent to the Canadian Archipelago, thus almost all Canadian polar bears (and most polar bears throughout the circumpolar basin) occur in in preferred habitat regions that are not well-resolved by GCMs and that have received less research attention. How climate warming is experienced by polar bears within the Canadian Archipelago will depend on the onset and duration of seasonal open water there (Derocher et al., 2004; Stirling & Derocher, 2012); and especially on sea ice state during the late spring and early summer hyperphagic hunting season (Stirling, 1998, 2011; Cherry et al., 2013). Changes in critical aspects of sea ice characteristics in polar bear subpopulations are difficult to anticipate because the ecological circumstances for each subpopulation are distinct (Thiemann et al., 2008), and because predictive sea ice models for the Archipelago are still developing (Sou & Flato, 2009). Given the uncertainty of future sea ice dynamics, especially in the Canadian Archipelago, and the uncertainty of demographic response of polar bear subpopulations to these changes; we suggest that predictions of declines in polar bear numbers and productivity based on current GCM forecasts are premature and unreliable.

The current view of the IPCC and most climate scientists is that long term climatic changes are dependent on the future path of greenhouse gas emissions (IPCC, 2007; Moss et al., 2010; IPCC; 2013). If emissions are limited – whether through concerted mitigation action (c.f. van Vuuren et al., 2011), exhaustion of fossil fuel reserves (c.f. Nel & Cooper, 2009), or some combination of the two – along the lines of the B1 (or RCP2.6) scenario then severe impacts on most subpopulations will be avoided (Amstrup et al., 2010). Given the apparent lack of numerical impact on polar bears after 130 years of anthropogenic global warming, and the ability of polar bears to survive warmer temperatures during the last interglacial (Ingólfsson & Wiig 2009; Lindqvist et al., 2010; Edwards et al., 2011); it seems unlikely that polar bears are at risk as a species (Fig. 2). There is good evidence for condition and recruitment impacts from reduced sea ice in the Baffin Bay, Southern Beaufort Sea, Southern Hudson Bay, and Western Hudson Bay subpopulations (Obbard et al., 2006; Regehr et al., 2006; 2007a, 2007b; Obbard et al., 2007; Peacock et al., 2012), however we show that M-R evidence for subpopulation decline in these areas is suspect, and also contradicted by other evidence (Tables 5, 6, 7, and 9; Fig. 5, 6, 7, and 8). We agree that these subpopulations should be monitored preferentially because they appear to be the most vulnerable to climatological changes in Arctic sea ice state (Stirling et al., 1999; 2004; Stirling & Parkinson, 2006; Regehr et al., 2007a, 2007b).

It is generally accepted that polar bears have increased in numbers as they recovered from over-hunting prior to the International Agreement on the Conservation of Polar Bears (Prestrud & Stirling, 1994; Lunn et al., 2002). Although contemporary management approaches did not explicitly recognize and accommodate progressive environmental effects until recently, the efforts to manage the harvest have resulted in secure and productive subpopulations of polar bears throughout most of Canada. Increased monitoring and adaptive management are warranted; however, trade restrictions and ultra-precautionary status designations that precede any actual decline in numbers or loss of range works against conservation because such practices reduce the credibility of polar bear management practices to aboriginal people who continue to harvest at sustainable levels for nutritional, cultural, and economic purposes.

**S7 Table S1.** HadCRUT4 global temperature, REMSS Arctic temperature, NSIDC sea ice extent, and NOAA-NODC ocean heat content data for the 1980-1996 period.

|  | *HADCRUT Annual Global Temp.* | *REMSS Annual Arctic Temp.* | *Sea Ice Extent 10^6 km sq.* | | | | | | | | | | | | *Ocean Heat Content* |
| --- | --- | --- | --- | --- | --- | --- | --- | --- | --- | --- | --- | --- | --- | --- | --- |
| **YEAR** | **TEMP (°C)** | | **JAN** | **FEB** | **MAR** | **APR** | **MAY** | **JUN** | **JUL** | **AUG** | **SEP** | **OCT** | **NOV** | **DEC** | **10^22^ Joules** |
| 1980 | 0.093 | 0.259 | 14.96 | 15.98 | 16.13 | 15.49 | 14.04 | 12.31 | 10.39 | 8.04 | 7.85 | 9.46 | 11.69 | 13.72 | 1.091 |
| 1981 | 0.1415 | 0.561 | 15.03 | 15.65 | 15.61 | 15.12 | 13.9 | 12.57 | 10.62 | 7.86 | 7.25 | 9.19 | 11.17 | 13.74 | 0.122 |
| 1982 | 0.0115 | -0.404 | 15.26 | 16.06 | 16.15 | 15.57 | 14.17 | 12.69 | 10.75 | 8.26 | 7.45 | 9.98 | 11.91 | 13.83 | -2.306 |
| 1983 | 0.190 | 0.084 | 15.1 | 16.02 | 16.1 | 15.3 | 13.54 | 12.36 | 10.91 | 8.36 | 7.52 | 9.64 | 11.64 | 13.44 | -2.763 |
| 1984 | -0.0145 | -0.079 | 14.61 | 15.32 | 15.62 | 15.15 | 13.68 | 12.2 | 10.15 | 7.87 | 7.17 | 8.84 | 11.29 | 13.18 | -0.459 |
| 1985 | -0.0285 | -0.14 | 14.86 | 15.67 | 16.06 | 15.34 | 14.23 | 12.4 | 10.09 | 7.46 | 6.93 | 8.88 | 11.39 | 13.19 | 0.11 |
| 1986 | 0.0465 | -0.223 | 15.02 | 15.89 | 16.08 | 15.15 | 13.52 | 12.1 | 10.47 | 8.01 | 7.54 | 9.89 | 11.78 | 13.4 | -1.037 |
| 1987 | 0.1855 | -0.442 | 15.2 | 16.11 | 15.95 | 15.33 | 13.81 | 12.57 | 9.98 | 7.69 | 7.48 | 9.29 | 11.52 | N/A | -0.893 |
| 1988 | 0.201 | 0.125 | N/A | 15.61 | 16.13 | 15.21 | 13.69 | 12.02 | 10.04 | 7.9 | 7.49 | 9.47 | 11.69 | 13.78 | 1.088 |
| 1989 | 0.121 | -0.143 | 15.12 | 15.56 | 15.52 | 14.44 | 12.98 | 12.31 | 10.38 | 7.92 | 7.04 | 9.52 | 11.5 | 13.47 | 0.903 |
| 1990 | 0.2925 | -0.195 | 14.95 | 15.56 | 15.88 | 14.68 | 13.3 | 11.68 | 9.62 | 6.82 | 6.24 | 9.35 | 11.31 | 13.27 | 0.177 |
| 1991 | 0.253 | 0.208 | 14.46 | 15.26 | 15.5 | 14.93 | 13.51 | 12.23 | 9.68 | 7.4 | 6.55 | 9.16 | 11.12 | 13.17 | 2.646 |
| 1992 | 0.102 | -0.542 | 14.72 | 15.5 | 15.47 | 14.7 | 13.25 | 12.13 | 10.61 | 7.86 | 7.55 | 9.6 | 11.87 | 13.46 | 0.572 |
| 1993 | 0.1435 | -0.186 | 15.08 | 15.73 | 15.88 | 15.18 | 13.54 | 11.99 | 9.66 | 7.29 | 6.5 | 9.18 | 11.73 | 13.52 | 0.684 |
| 1994 | 0.2045 | -0.060 | 14.82 | 15.61 | 15.58 | 14.95 | 13.73 | 12.1 | 10.22 | 7.61 | 7.18 | 9.48 | 11.3 | 13.53 | 1.51 |
| 1995 | 0.323 | 0.581 | 14.62 | 15.24 | 15.32 | 14.59 | 13.04 | 11.55 | 9.15 | 6.68 | 6.13 | 8.94 | 10.97 | 12.98 | 2.264 |
| 1996 | 0.1775 | 0.233 | 14.21 | 15.17 | 15.13 | 14.22 | 13.06 | 12.1 | 10.36 | 8.17 | 7.88 | 9.39 | 10.56 | 13.14 | 4.544 |

**S7 Table S2.** HADCRUT4 global temperature, REMSS Arctic temperature, NSIDC sea ice extent, and NOAA-NODC ocean heat content data for the 1997-2013 period.

|  | *HADCRUT Annual Global Temp.* | *REMSS Annual Arctic Temp.* | *Sea Ice Extent 10^6 km sq.* | | | | | | | | | | | | *Ocean Heat Content* |
| --- | --- | --- | --- | --- | --- | --- | --- | --- | --- | --- | --- | --- | --- | --- | --- |
| **YEAR** | **TEMP (°C)** | | **JAN** | **FEB** | **MAR** | **APR** | **MAY** | **JUN** | **JUL** | **AUG** | **SEP** | **OCT** | **NOV** | **DEC** | **10^22^ Joules** |
| 1997 | 0.392 | 0.313 | 14.47 | 15.52 | 15.58 | 14.59 | 13.32 | 11.91 | 9.59 | 7.3 | 6.74 | 8.76 | 10.91 | 13.29 | 3.245 |
| 1998 | 0.530 | 0.397 | 14.81 | 15.77 | 15.66 | 14.89 | 13.8 | 11.85 | 9.62 | 7.49 | 6.56 | 8.85 | 10.75 | 13.26 | 4.304 |
| 1999 | 0.301 | 0.186 | 14.47 | 15.37 | 15.4 | 15.13 | 13.86 | 12.1 | 9.59 | 7.38 | 6.24 | 9.1 | 10.99 | 12.88 | 5.943 |
| 2000 | 0.295 | 0.511 | 14.41 | 15.18 | 15.27 | 14.63 | 13.18 | 11.71 | 9.75 | 7.21 | 6.32 | 8.92 | 10.54 | 12.81 | 5.857 |
| 2001 | 0.4385 | 0.443 | 14.31 | 15.27 | 15.61 | 14.86 | 13.72 | 11.69 | 9.22 | 7.47 | 6.75 | 8.59 | 10.92 | 12.84 | 4.117 |
| 2002 | 0.494 | 0.508 | 14.45 | 15.36 | 15.44 | 14.37 | 13.12 | 11.69 | 9.49 | 6.53 | 5.96 | 8.81 | 10.78 | 12.82 | 6.789 |
| 2003 | 0.505 | 0.841 | 14.46 | 15.25 | 15.49 | 14.57 | 13 | 11.77 | 9.46 | 6.85 | 6.15 | 8.65 | 10.29 | 12.82 | 9.952 |
| 2004 | 0.4455 | 0.307 | 14.03 | 14.93 | 15.05 | 14.11 | 12.58 | 11.51 | 9.6 | 6.83 | 6.05 | 8.48 | 10.65 | 12.72 | 10.24 |
| 2005 | 0.5415 | 1.093 | 13.66 | 14.36 | 14.74 | 14.07 | 12.99 | 11.29 | 8.93 | 6.3 | 5.57 | 8.45 | 10.47 | 12.47 | 8.412 |
| 2006 | 0.496 | 0.705 | 13.6 | 14.42 | 14.43 | 13.97 | 12.62 | 11.06 | 8.67 | 6.52 | 5.92 | 8.33 | 9.84 | 12.27 | 10.43 |
| 2007 | 0.4845 | 0.821 | 13.77 | 14.53 | 14.65 | 13.87 | 12.89 | 11.49 | 8.13 | 5.36 | 4.3 | 6.77 | 10.05 | 12.39 | 9.478 |
| 2008 | 0.3885 | 0.502 | 14.05 | 15.01 | 15.22 | 14.42 | 13.19 | 11.36 | 8.99 | 6.05 | 4.73 | 8.42 | 10.62 | 12.52 | 10.052 |
| 2009 | 0.495 | 0.427 | 14.08 | 14.85 | 15.14 | 14.57 | 13.4 | 11.46 | 8.8 | 6.28 | 5.39 | 7.52 | 10.27 | 12.51 | 10.126 |
| 2010 | 0.5475 | 1.175 | 13.8 | 14.59 | 15.11 | 14.7 | 13.11 | 10.82 | 8.36 | 6.01 | 4.93 | 7.71 | 9.88 | 12.02 | 10.367 |
| 2011 | 0.4085 | 0.791 | 13.57 | 14.38 | 14.58 | 14.16 | 12.81 | 10.99 | 7.91 | 5.55 | 4.63 | 7.14 | 10 | 12.4 | 10.869 |
| 2012 | 0.451 | 0.949 | 13.77 | 14.59 | 15.24 | 14.72 | 13.12 | 10.92 | 7.93 | 4.71 | 3.63 | 7.07 | 9.92 | 12.2 | 10.941 |
| 2013 | 0.488 | 0.486 | 13.66 | 14.66 | 14.99 | 14.3 | 13.04 | 11.43 | 8.23 | 6.05 | 5.24 | 7.4 | 9.95 | 12.18 | 12.601 |

**S7 Table S3.** Examining Ho: slope = 0 for world ocean heat content, global atmospheric temperature, Arctic (60-82.5 N) atmospheric temperature, and annual Arctic sea ice extent vs. time. The probability of a Type II error (false negative rate) is β, and the power is equal to (1−β).

|  |  |  | Bivariate Correlation | | Linear Regression | | | | Power |
| --- | --- | --- | --- | --- | --- | --- | --- | --- | --- |
| **Variable** | **Period** | **Sample Size** | **Pearson’s R** | **p ≤** | **R Square** | **Intercept** | **Slope** | **p ≤** | **(1- β)** |
| Ocean Heat | 1980 to 2013 | 34 | 0.951 | 0.001 | 0.904 | -884.227 | 0.445 | 0.001 | 1.0 |
| Ocean Heat | 2010 to 2013 | 4 | 0.90 | 0.10 | 0.810 | -1351.396 | 0.677 | 0.10 | 0.583 |
| Ocean Heat | 1980 to 2010 | 31 | 0.936 | 0.001 | 0.877 | -879.417 | 0.493 | 0.001 | 1.0 |
| Global Temp | 1980 to 2013 | 34 | 0.870 | 0.001 | 0.757 | -31.185 | 0.016 | 0.001 | 1.0 |
| Global Temp | 1997 to 2013 | 17 | 0.342 | 0.179 | 0.117 | -9.767 | 0.005 | 0.179 | 0.282 |
| Global Temp | 1980 to 1997 | 18 | 0.646 | 0.004 | 0.418 | -27.178 | 0.014 | 0.004 | 0.933 |
| Arctic Temp | 1980 to 2013 | 34 | 0.723 | 0.001 | 0.523 | -63.506 | 0.032 | 0.001 | 1.0 |
| Arctic Temp | 1998 to 2013 | 16 | 0.488 | 0.055 | 0.367 | -78.339 | 0.029 | 0.055 | 0.548 |
| Arctic Temp | 1980 to 1998 | 19 | 0.20 | 0.412 | 0.040 | -22.978 | 0.012 | 0.412 | 0.141 |
| Sea Ice Extent | 1980 to 2013 | 34 | -0.930 | 0.001 | 0.865 | 120.710 | 0.055 | 0.001 | 1.0 |
| Sea Ice Extent | 2004 to 2013 | 10 | -0.587 | 0.074 | 0.345 | 108.851 | -0.049 | 0.074 | 0.531 |
| Sea Ice Extent | 1980 to 2004 | 25 | -0.885 | 0.001 | 0.782 | 90.842 | -0.040 | 0.001 | 1.0 |

**S7 Table S4.** Examining Ho: slope = 0 for monthly Arctic sea ice extent vs. time for the January 1980 to December 2013 period. The probability of a Type II error (false negative rate) is β, and the power is equal to (1−β).

|  |  | January 1980 to December 2013 | | | | | | |
| --- | --- | --- | --- | --- | --- | --- | --- | --- |
|  |  | Bivariate Correlation | | Linear Regression | | | | Power |
| **Month** | **Sample Size** | **Pearson’s R** | **p ≤** | **R Square** | **Intercept** | **Slope** | **p ≤** | **(1- β)** |
| *January* | 33/34 | -0.893 | 0.001 | 0.797 | 108.258 | -0.047 | 0.001 | 1.0 |
| *February* | 34 | -0.863 | 0.001 | 0.745 | 103.805 | -0.044 | 0.001 | 1.0 |
| *March* | 34 | -0.818 | 0.001 | 0.669 | 91.854 | -0.038 | 0.001 | 1.0 |
| *April* | 34 | -0.764 | 0.001 | 0.583 | 84.690 | -0.035 | 0.001 | 1.0 |
| *May* | 34 | -0.698 | 0.001 | 0.487 | 73.218 | -0.030 | 0.001 | 1.0 |
| *June* | 34 | -0.895 | 0.001 | 0.802 | 100.107 | -0.044 | 0.001 | 1.0 |
| *July* | 34 | -0.892 | 0.001 | 0.796 | 158.457 | -0.075 | 0.001 | 1.0 |
| *August* | 34 | -0.860 | 0.001 | 0.740 | 163.214 | -0.078 | 0.001 | 1.0 |
| *September* | 34 | -0.861 | 0.001 | 0.741 | 192.759 | -0.093 | 0.001 | 1.0 |
| *October* | 34 | -0.838 | 0.001 | 0.702 | 147.599 | -0.070 | 0.001 | 1.0 |
| *November* | 34 | -0.895 | 0.001 | 0.802 | 126.766 | -0.058 | 0.001 | 1.0 |
| *December* | 33/34 | -0.915 | 0.001 | 0.837 | 107.764 | -0.047 | 0.001 | 1.0 |

**S7 Table S5.** Examining Ho: slope = 0 for monthly Arctic sea ice extent vs. time for the period at which the relationship between the two variables was no longer significant. The probability of a Type II error (false negative rate) is β, and the power is equal to (1−β).

|  |  |  | Bivariate Correlation | | Linear Regression | | | | Power |
| --- | --- | --- | --- | --- | --- | --- | --- | --- | --- |
| **Period** | **Month** | **Sample Size** | **Pearson’s R** | **p ≤** | **R Square** | **Intercept** | **Slope** | **p ≤** | **(1- β)** |
| 2003 to 2013 | *January* | 12 | -0.515 | 0.105 | 0.265 | 97.830 | -0.042 | 0.105 | 0.434 |
| 2002 to 2013 | *February* | 13 | -0.548 | 0.065 | 0.300 | 117.014 | -0.051 | 0.065 | 0.538 |
| 1999 to 2013 | *March* | 15 | -0.467 | 0.080 | 0.218 | 89.026 | -0.037 | 0.080 | 0.477 |
| 1994 to 2013 | *April* | 20 | -0.394 | 0.086 | 0.155 | 60.655 | -0.023 | 0.086 | 0.441 |
| 1995 to 2013 | *May* | 19 | -0.365 | 0.124 | 0.133 | 58.820 | -0.023 | 0.124 | 0.371 |
| 2003 to 2013 | *June* | 11 | -0.558 | 0.074 | 0.312 | 111.134 | -0.050 | 0.074 | 0.517 |
| 2005 to 2013 | *July* | 9 | -0.661 | 0.052 | 0.438 | 212.352 | -0.101 | 0.052 | 0.629 |
| 2004 to 2013 | *August* | 10 | -0.622 | 0.055 | 0.387 | 259.402 | -0.126 | 0.055 | 0.596 |
| 2004 to 2013 | *September* | 10 | -0.607 | 0.063 | 0.368 | 306.557 | -0.150 | 0.063 | 0.563 |
| 2005 to 2013 | *October* | 9 | -0.578 | 0.103 | 0.335 | 273.838 | -0.132 | 0.103 | 0.454 |
| 2003 to 2013 | *November* | 11 | -0.578 | 0.063 | 0.334 | 114.770 | -0.052 | 0.063 | 0.552 |
| 2005 to 2013 | *December* | 9 | -0.492 | 0.179 | 0.242 | 74.273 | -0.031 | 0.179 | 0.314 |

**S7 Table S6.** Examining Ho: slope = 0 for monthly Arctic sea ice extent vs. time for the period prior to the breakpoint, at which the relationship between the two variables was no longer significant. The probability of a Type II error (false negative rate) is β, and the power is equal to (1−β).

|  |  |  | Bivariate Correlation | | Linear Regression | | | | Power |
| --- | --- | --- | --- | --- | --- | --- | --- | --- | --- |
| **Period** | **Month** | **Sample Size** | **Pearson’s R** | **p ≤** | **R Square** | **Intercept** | **Slope** | **p ≤** | **(1- β)** |
| 1980 to 2003 | *January* | 23/24 | -0.740 | 0.001 | 0.547 | 78.659 | -0.032 | 0.001 | 0.999 |
| 1980 to 2002 | *February* | 23 | -0.654 | 0.001 | 0.428 | 71.241 | -0.028 | 0.001 | 0.990 |
| 1980 to 1999 | *March* | 20 | -0.675 | 0.001 | 0.455 | 86.238 | -0.035 | 0.001 | 0.965 |
| 1980 to 1994 | *April* | 15 | -0.633 | 0.011 | 0.401 | 103.098 | -0.044 | 0.011 | 0.825 |
| 1980 to 1995 | *May* | 16 | -0.657 | 0.006 | 0.432 | 113.960 | -0.050 | 0.006 | 0.889 |
| 1980 to 2003 | *June* | 24 | -0.781 | 0.001 | 0.609 | 80.861 | -0.035 | 0.001 | 1.0 |
| 1980 to 2005 | *July* | 26 | -0.780 | 0.001 | 0.608 | 116.345 | -0.053 | 0.001 | 1.0 |
| 1980 to 2004 | *August* | 25 | -0.701 | 0.001 | 0.491 | 104.096 | -0.048 | 0.001 | 0.996 |
| 1980 to 2004 | *September* | 25 | -0.712 | 0.001 | 0.507 | 123.479 | -0.059 | 0.001 | 0.999 |
| 1980 to 2005 | *October* | 26 | -0.706 | 0.001 | 0.498 | 86.022 | -0.039 | 0.001 | 0.999 |
| 1980 to 2003 | *November* | 24 | -0.761 | 0.001 | 0.579 | 109.043 | -0.049 | 0.001 | 1.0 |
| 1980 to 2005 | *December* | 25/26 | -0.828 | 0.001 | 0.686 | 91.649 | -0.039 | 0.001 | 1.0 |

**S7 Table S7.** Examining: correlations for annual global temperatures vs. monthly Arctic sea ice extent for the 1980-2013 period.

|  |  | January 1980 to December 2013 | | | | | |
| --- | --- | --- | --- | --- | --- | --- | --- |
|  |  | Bivariate Correlation | | Linear Regression | | | |
| **Month** | **Sample Size** | **Pearson’s R** | **p ≤** | **R Square** | **Intercept** | **Slope** | **p ≤** |
| *January* | 33/34 | -0.749 | 0.001 | 0.562 | 15.118 | -2.161 | 0.001 |
| *February* | 34 | -0.710 | 0.001 | 0.503 | 15.894 | -2.010 | 0.001 |
| *March* | 34 | -0.691 | 0.001 | 0.477 | 15.994 | -1.783 | 0.001 |
| *April* | 34 | -0.697 | 0.001 | 0.486 | 15.270 | -1.763 | 0.001 |
| *May* | 34 | -0.621 | 0.001 | 0.386 | 13.814 | -1.472 | 0.001 |
| *June* | 34 | -0.808 | 0.001 | 0.653 | 12.491 | -7.752 | 0.001 |
| *July* | 34 | -0.786 | 0.001 | 0.617 | 10.650 | -3.622 | 0.001 |
| *August* | 34 | -0.738 | 0.001 | 0.738 | 8.195 | -3.698 | 0.001 |
| *September* | 34 | -0.734 | 0.001 | 0.734 | 7.688 | -4.389 | 0.001 |
| *October* | 34 | -0.710 | 0.001 | 0.503 | 9.741 | -3.247 | 0.001 |
| *November* | 34 | -0.832 | 0.001 | 0.693 | 11.808 | -2.976 | 0.001 |
| *December* | 33/34 | -0.782 | 0.001 | 0.612 | 13.676 | -2.218 | 0.001 |

**S7 Table S8.** Examining correlations for annual global temperatures vs. monthly Arctic sea ice extent for the period at which the relationship between the two variables was no longer significant. The probability of a Type II error (false negative rate) is β, and the power is equal to (1−β).

|  |  |  | Bivariate Correlation | | Linear Regression | | | |
| --- | --- | --- | --- | --- | --- | --- | --- | --- |
| **Period** | **Month** | **Sample Size** | **Pearson’s R** | **p ≤** | **R Square** | **Intercept** | **Slope** | **p ≤** |
| 1995 to 2013 | *January* | 19 | -0.328 | 0.170 | 0.108 | 14.671 | -1.284 | 0.170 |
| 1994 to 2013 | *February* | 20 | -0.435 | 0.055 | 0.189 | 15.731 | -1.729 | 0.055 |
| 1993 to 2013 | *March* | 21 | -0.413 | 0.063 | 0.170 | 15.736 | -1.28- | 0.063 |
| 1992 to 2013 | *April* | 22 | -0.422 | 0.050 | 0.178 | 14.968 | -1.123 | 0.050 |
| 1987 to 2013 | *May* | 27 | -0.362 | 0.063 | 0.131 | 13.570 | -0.900 | 0.063 |
| 1997 to 2013 | *June* | 17 | -0.406 | 0.106 | 0.165 | 12.371 | -1.977 | 0.106 |
| 1997 to 2013 | *July* | 17 | -0.318 | 0.214 | 0.101 | 10.199 | -2.734 | 0.214 |
| 1997 to 2013 | *August* | 17 | -0.267 | 0.301 | 0.071 | 7.747 | -2.824 | 0.301 |
| 1996 to 2013 | *September* | 18 | -0.455 | 0.058 | 0.207 | 7.831 | -4.806 | 0.058 |
| 1996 to 2013 | *October* | 18 | -0.466 | 0.055 | 0.211 | 9.842 | -3.645 | 0.055 |
| 1995 to 2013 | *November* | 19 | -0.445 | 0.056 | 0.198 | 11.227 | -1.819 | 0.056 |
| 1996 to 2013 | *December* | 18 | -0.455 | 0.058 | 0.207 | 13.404 | -1.737 | 0.058 |

**S7 Table S9.** Comparing Arctic atmospheric temperature means for the time periods identified by a breakpoint regression of Arctic temperatures since 1980 (Table H3).

| **Period** | **N** | **Mean (SE)** | **t-test; Ho: difference = 0** | | | **Levene's Test for Equality of Variances** | |
| --- | --- | --- | --- | --- | --- | --- | --- |
|  |  |  | **Mean Difference** | **SE Difference** | **p ≤** | **F** | **p ≤** |
| 1980-1998 | 19 | 0.018 (0.075) | -0.616 | 0.104 | 0.001 | 0.380 | 0.542 |
| 1998-2013 | 16 | 0.634 (0.071) |  |  |  |  |  |

REFERENCES

Aars J, Lunn NJ, Derocher AE (2006) Polar Bears: Proceedings of the 14th Working Meeting of the IUCN/SSC Polar Bear Specialist Group, 20–24 June 2005, Seattle, Washington, USA, v + 191 pp.

Amstrup SC, Stirling I, Lentfer J (1986) Size and trends of Alaskan polar bear populations. Wildlife Society Bulletin, 14, 251-254.

Amstrup SC, DeMaster DP (1988) Polar bear—Ursus maritimus. Selected marine mammals of Alaska: Species accounts with research and management recommendations. In: Marine Mammal Commission (eds. Lentfer JW) pp. 39-56, Washington, DC.

Amstrup SC, Gardner C (1994) Polar bear maternity denning in the Beaufort Sea. The Journal of Wildlife Management, 58, 1-10.

Amstrup SC (1995) Movements, distribution, and population dynamics of polar bears in the Beaufort Sea. Ph.D. Dissertation, University of Alaska Fairbanks, Fairbanks.

Amstrup SC, McDonald TL, Stirling I (2001) Polar bears in the Beaufort Sea: a 30-year mark-recapture case history. Journal of Agricultural, Biological, and Environmental Statistics 6:221-234.

Amstrup SC, McDonald TL, Durner GM (2004) Using satellite radio telemetry data to delineate and manage wildlife populations. Wildlife Society Bulletin, 32, 661-679.

Amstrup SC, Marcot BG, Douglas DC (2007) Forecasting the Range-wide Status of Polar Bears at Selected Times in the 21st Century. USGS Science Strategy to Support U.S. Fish and Wildlife Service. Polar Bear Listing Decision, Administrative Report, US Department of the Interior/US Geological Survey, Virginia, USA, vi + 126 pp.

Amstrup SC, Marcot BG, Douglas DC (2008) A Bayesian Network Modeling Approach to Forecasting the 21st Century Worldwide Status of Polar Bears. In: Arctic Sea Ice Decline: Observations, Projections, Mechanisms, and Implications. Geophysical Monograph, 180 (eds. DeWeaver ET, Bitz CM, Tremblay LB), pp. 213-268. American Geophysical Union, Washington DC.

Amstrup SC, DeWeaver ET, Douglas DC, Marcot BG, Durner GM, Bitz CM, Bailey DA (2010) Greenhouse gas mitigation can reduce sea-ice loss and increase polar bear persistence. Nature, 468, 955-958.

Amstrup SC, Stirling I, Lentfer JW, Gardner C, Durner GM, Manly B (2011) Polar bears and climate change: Certainties, uncertainties, and hope in a warming world. Gyrfalcons and Ptarmigan in a Changing World. The Peregrine Fund, Boise, Idaho, USA.

Atatahak G, Banci V (eds.) (2001) Traditional knowledge polar bear report. Prepared for the Department of Sustainable Development, Kugluktuk, NU, 15 pp.

Bethke R, Taylor MK, Amstrup S, Messier F (1996) Population delineation of polar bears using satellite collar data. Journal of Applied Ecology, 6, 311-317.

Born EW (2002) Research on polar bears in Greenland, 1997–2001. pp. 71 in Lunn N, Schliebe S, Born EW (eds). Polar Bear Specialist Group, 23–28 June 2001, Nuuk, Greenland. Occassional Paper of the IUCN Species Survival Commission No. 26. IUCN, Gland, Switzerland and Cambridge, UK.

Born EW (2005) The catch of polar bears in Greenland, 1993–2004. Report to the Canadian Polar Bear Technical Committee’s meeting, Edmonton, Canada. Greenland Institute of Natural Resources, Nuuk, Greenland.10 pp.

Born EW, Sonne C (2005) Research on polar bears in Greenland 2001 to 2005. Report to the 14th Meeting of the IUCN Polar Bear Specialist Group. Greenland Institute of Natural Resources, Nuuk, Greenland, 14 pp.

Brice-Bennett C (1976) Inuit land use in the east-central Canadian arctic. Department of Indian and Northern Affairs, Ottawa, ON. In: Inuit land use and occupancy project, Vol. 1. (ed. Freeman M) pp. 63-81, Department of Indian and Northern Affairs, Ottawa, ON.

Brody, H. 1976. Inuit land use in North Baffin Island and Northern Foxe Basin. In: Inuit land use and occupancy project, Vol. 1. (ed. Freeman M) pp. 153-171, Department of Indian and Northern Affairs, Ottawa, ON.

Brower CD, Carpenter A, Branigan ML, Calvert W, Evans T, Fischbach AS, Nagy JA, Schliebe S, Stirling I (2002) The polar bear management agreement for the southern Beaufort Sea: an evaluation of the first ten years of a unique conservation agreement. Arctic, 55, 362–372.

Burnham KP (1993) A theory for combined analysis of ring recovery and recapture data. In: Marked individuals in the study of bird populations (eds. Lebreton JD, North PM) pp. 199–213, Basel, Switzerland: Birkhäuser Verlag.

Cherry SG, Derocher AE, Thiemann GW, Lunn NJ (2013) Migration phenology and seasonal fidelity of an Arctic marine predator in relation to sea ice dynamics. Journal of Animal Ecology, 82, 912-921.

Cohen J (1988) Statistical Power Analysis for the Behavioural Sciences (2^nd^ ed.) Hillsdale, NJ: Lawrence Erlbaum Associates, Publishers.

Comiso JC, Parkinson CL, Gersten R, Stock L (2008) Accelerated decline in the Arctic sea ice cover. Geophysical Research Letters, 35, L01703.

Committee on the Status of Endangered Wildlife in Canada (COSEWIC) (2008) COSEWIC assessment and update status report on the polar bear Ursus maritimus in Canada. Committee on the Status of Endangered Wildlife in Canada, Ottawa, vii + 75 pp.

Crête M, Vandal D, Rivest LP, Potvin F (1991) Double counts in aerial surveys to estimate polar bear numbers in the ice-free season. Arctic, 44, 275-278.

Crompton A (2004) A genetic assignment of the population structure of polar bears (*Ursus maritimus*) in the greater Hudson Bay ecosystem. M.Sc. Thesis, Trent University, Peterborough, Ontario.

DeMaster DP, Kingsley MCS, Stirling I (1980) A multiple mark and recapture estimate applied to polar bears. Canadian Journal of Zoology, 58, 644-658.

Derocher AE, Stirling I. (1990) Distribution of polar bears (Ursus maritimus) during the ice-free period in western Hudson Bay. Canadian Journal of Zoology, 68, 1395-1403.

Derocher AE, Stirling I (1992) The population dynamics of polar bears in western Hudson Bay. In: Wildlife 2001: Populations (eds. McCullough DR, Barrett RH), pp. 1150-1159, Elsevier, Amsterdam.

Derocher AE, Stirling I (1995a) Temporal variation in reproduction and body mass of polar bears in western Hudson Bay. Canadian Journal of Zoology, 73, 1657-1665.

Derocher AE, Stirling I (1995b) Mark-recapture estimation of population size and survival rates for polar bears in western Hudson Bay. Journal of Wildlife Management, 59, 215-221.

Derocher AE, Stirling I, Calvert W (1997b) Male-biased harvesting of polar bears in Western Hudson Bay*.* Journal of Wildlife Management, 61, 1075-1082.

Derocher AE, Lunn NJ, Stirling I (2004) Polar bears in a warming climate. Integrative and Comparative Biology, 44, 163-176.

Derocher AE, Aars J, Amstrup SC et al. (2013) Rapid ecosystem change and polar bear conservation. Conservation Letters, 6, 368-375.

Douglass DH, Knox RS (2012) Ocean heat content and Earth’s radiation imbalance. II. Relation to climate shifts. Physics Letters A, 376, 1226–1229.

Dowsley M (2005) Inuit knowledge regarding climate change and the Baffin Bay polar bear population. Government of Nunavut, Department of Environment, Final Wildlife Report 1, Iqaluit, Nunavut, 43 pp.

Dowsley M, Taylor, MK (2006a) Community consultations with Qikiqtarjuaq, Clyde River and Pond Inlet on management concerns for the Baffin Bay (BB) polar bear population: A summary of Inuit knowledge and community consultations. Nunavut Wildlife Research Group Final Report, 83 pp.

Dowsley M, Taylor MK (2006b) Management consultations for the Western Hudson Bay (WH) polar bear population (01-02 December 2005). Nunavut Wildlife Research Group Final Report, 55 pp.

Durner GM, Amstrup SC, Nielson R, McDonald T (2004) Using discrete choice modeling to generate resource selection functions for female polar bears in the Beaufort Sea. In Resource selection methods and applications. In: Resource selection methods and applications (ed. Huzurbazar S) pp. 107–120, Proceedings of the First International Conference on Resource Selection, 13–15 January 2003, Laramie, Wyoming, USA.

Durner GM, Douglas DC, Nielson RM et al. (2009) Predicting 21st-century polar bear habitat distribution from global climate models. Ecological Monographs, 79, 25–58.

Edwards CJ, Suchard MA, Lemey P, et al. (2011) Ancient Hybridization and an Irish Origin for the Modern Polar Bear Matriline. Current Biology, 21, 1251-1258.

Farquharson DR (1976) Inuit land use in the west-central Canadian arctic. In: Inuit land use and occupancy project, Vol. 1. (ed. Freeman M) pp. 33-61, Department of Indian and Northern Affairs, Ottawa, ON.

Faul F, Erdfelder E, Lang AG, Buchner A (2007) G* Power 3: A flexible statistical power analysis program for the social, behavioral, and biomedical sciences. Behavior research methods, 39, 175-191.

Ferguson SH, Stirling I, McLoughlin PD (2005) Climate change and ringed seal (Phoca hispida) recruitment in western Hudson Bay*.* Marine Mammal Science, 21, 121-135.

Furnell DJ, Schweinsburg RE (1984) Population dynamics of central Arctic polar bears. Journal of Wildlife Management, 48, 722-728.

Hadley Centre of the UK Met Office (HadCRUT4). Met Office Hadley Centre observations datasets. <http://www.metoffice.gov.uk/hadobs/hadcrut4/data/current/download.html> (accessed 20 January 2014).

Harwood LA, Smith TG, Auld JC (2012) Fall migration of ringed seals (Phoca hispida) through the Beaufort and Chukchi seas, 2001–02. Arctic, 65, 35-44.

Hunter CM, Caswell H, Runge MC, Regehr EV, Amstrup SC, Stirling I (2010) Climate change threatens polar bear populations: a stochastic demographic analysis. Ecology, 9, 2883–2897.

Ingólfsson Ó, Wiig Ø (2008) Late Pleistocene fossil find in Svalbard: the oldest remains of a polar bear (Ursus maritimus Phipps, 1744) ever discovered. Polar Research, 28, 455-462.

Intergovernmental Panel on Climate Change (IPCC) (2007) Climate Change 2007: Synthesis Report. Contribution of Working Groups I, II and III to the Fourth Assessment Report of the Intergovernmental Panel on Climate Change (eds. Pachauri RK, Reisinger A) IPCC, Geneva, Switzerland, 104 pp.

Intergovernmental Panel on Climate Change (IPCC) (2013) Climate Change 2013: The Physical Science Basis. Contribution of Working Group I to the Fifth Assessment Report of the Intergovernmental Panel on Climate Change (eds. Stocker TF, Qin D, Plattner GK et al.) pp.1535, Cambridge University Press, Cambridge, United Kingdom and New York, NY, USA. Jonkel, C., Smith, P., Stirling, I., and Kolenosky, G.B. 1976. The present status of the polar bear in the James Bay and Belcher Islands area. Occasional Paper No. 26, Canadian Wildlife Service, Ottawa, ON.

Jonkel C, Smith P, Stirling I, Kolenosky GB (1976) The present status of the polar bear in the James Bay and Belcher Islands area Occ. Pap. No. 26. Canadian Wildlife Service, Ottawa, ON.

Keith D, Arqvik J, Kamookak L, Ameralik J, the Gjoa Haven Hunters’ and Trappers’ Organization (2005) Inuit Qaujimaningit Nanurnut: Inuit knowledge of polar bears. Edmonton, AB: Gjoa Haven Hunters’ and Trappers’ Organization and CCI Press, vii + 242 pp.

Kingsley MCS, Stirling I, Calvert W (1985) The distribution and abundance of seals in the Canadian High Arctic, 1980–82. Canadian Journal of Fisheries and Aquatic Sciences, 42, 1189-1210.

Kirtman B, Power SB, Adedoyin JA et al. (2013) Near-term climate change: Projections and predictability. In: Climate Change 2013: The Physical Science Basis. Contribution of Working Group I to the Fifth Assessment Report of the Intergovernmental Panel on Climate Change (eds. Stocker TF, Qin D, Plattner GK et al.) pp. 953-1028, Cambridge University Press, Cambridge, United Kingdom and New York, NY, USA.

Kolenosky GB, Prevett JP (1983) Productivity and maternity denning of polar bears in Ontario. International Conference on Bear Research and Management, 5, 238-245.

Kolenosky GB, Abraham KF, Greenwood CJ (1992) Polar bears of Southern Hudson Bay. Polar Bear Project, 1984-88. Final Report. Unpublished report, Ontario Ministry of Natural Resources, Maple, Ontario, 107 pp.

Kwok R, Cunningham GF, Wensnahan M, Rigor I, Zwally HJ, Yi D (2009) Thinning and volume loss of the Arctic Ocean sea ice cover: 2003–2008. Journal of Geophysical Research 114: C07005, 16 pp.

Lentfer JW (1975) Polar bear denning on drifting sea ice. Journal of Mammalogy, 56, 716.

Levitus S, Antonov JI, Boyer TP et al. (2012) World ocean heat content and thermosteric sea level change (0–2000 m), 1955–2010. Geophysical Research Letters, 39, 1-5.

Lindqvist C, Schuster SC, Sun Y et al. (2010) Complete mitochondrial genome of a Pleistocene jawbone unveils the origin of polar bear. PNAS, 1-5. Proceedings of the National Academy of Sciences of the United States of America (PNAS), 107, 5053-5057 pp.

Lunn NJ, Stirling I, Andriashek D (1995) Movements and distribution of polar bears in the northeastern Beaufort Sea and McClure Strait. Final Report by Canadian Wildlife Service to the Inuvialuit Wildlife Management Advisory Committee. Inuvik, NWT, 65 pp.

Lunn NJ, Stirling I, Andriashek D, Kolenosky GB (1997) Re-estimating the size of the polar bear population in western Hudson Bay. Arctic, 50, 234-240.

Lunn NJ, Schliebe S, Born EW (2002) Proceedings of the 13th Working Meeting of the IUCN/SSC Polar Bear Specialist Group, 23–28 June 2001, Nuuk, Greenland, vii + 155 pp.

Lunn NJ, Stirling I, Richardson E, Andriashek D, Calvert W, Thiemann G, Davis C (2006) Canadian Wildlife Service report. Report to the Canadian Polar Bear Technical Committee, St. Johns, Newfoundland, Canada. Canadian Wildlife Service, Edmonton, AB, 17 pp.

Manabe S, Stouffer RJ (1980) Sensitivity of a global climate model to an increase of CO_2_ concentration in the atmosphere. Journal of Geophysical Research, 85, 5529–5554.

Maslanik J, Stroeve J, Fowler C, Emery W (2011) Distribution and trends in Arctic sea ice age through spring 2011. Geophysical Research Letters, 38, 1-6.

Maslowski W, Clement Kinney J, Higgins M, Roberts A (2012) The future of arctic sea ice. Annual Review of Earth and Planetary Sciences, 40, 625-654.

McDonald M, Arragutainaq L, Novalinga Z (eds.) (1997) Voices from the Bay: Traditional ecological knowledge of Inuit and Cree in the Hudson Bay Bioregion. Canadian Arctic Resources Committee and the Environmental Committee of the Municipality of Sanikiluaq, Ottawa, ON.

Meehl GA, Arblaster JM, Fasullo JT, Hu A, Trenberth KE (2011) Model-based evidence of deep-ocean heat uptake during surface-temperature hiatus periods. Nature Climate Change 1, 360-364.

Messier F, Taylor MK, Ramsay MA (1992) Seasonal activity patterns of female polar bears in the Canadian Arctic as revealed by satellite telemetry. Journal of Zoology, 226, 219-229.

Messier F, Taylor MK, Ramsay MA (1994) Denning ecology of polar bears in the Canadian Arctic Archipelago. Journal of Mammalogy, 75, 420-430.

Moss RH, Edmonds JA, Hibbard KA et al. (2010) The next generation of scenarios for climate change research and assessment. Nature, 463, 747-756.

Nageak BP, Brower CDN, Schliebe SL (1991) Polar bear management in the southern Beaufort Sea: An agreement between the Inuvialuit Game Council and North Slope Burrough Fish and Game Committee. Transactions from the North American Wildlife and Natural Resources Conference, 59, 337-343.

National Oceanographic Data Center (NODC). World Ocean Heat Content (0-700m) <http://data.nodc.noaa.gov/woa/DATA_ANALYSIS/3M_HEAT_CONTENT/DATA/basin/yearly/h22-w0-700m.dat> (accessed 20 January 2014).

National Snow and Ice Data Center (NSIDC). Sea Ice Index. 3.1.6 Monthly Sea Ice Extent and Area Data Files. ftp://sidads.colorado.edu/DATASETS/NOAA/G02135/ (accessed 20 January 2014).

Nel WP, Cooper CJ (2009) Implications of fossil fuel constraints on economic growth and global warming, Energy Policy, 37, 166-180.

Nunavut Tunngavik Incorporated (NTI) (2005) What if the winter doesn’t come? Inuit perspectives on climate change adaptation challenges in Nunavut. Summary Workshop Report, 15-17 March 2005. Iqaluit, NU.

Obbard, ME, Cattet MRL, Moody T, Walton LR, Potter D, Inglis J, Chenier C (2006) Temporal trends in the body condition of Southern Hudson Bay polar bears. Climate Change Research Information Note, No. 3. Applied Research and Development Branch, Ontario Ministry of Natural Resources, Sault Ste. Marie, ON.

Obbard ME, McDonald TL, Howe EJ, Regehr EV, Richardson ES (2007) Trends in abundance and survival for polar bears from Southern Hudson Bay, Canada, 1984–2005. USGS Alaska Science Center, Anchorage, Administrative Report, 36 pp.

Obbard ME, Thiemann GW, Peacock E, DeBruyn TD (2010) Polar Bears: Proceedings of the 15th Working Meeting of the IUCN/SSC Polar Bear Specialist Group, 29 June-3 July 2009, Copenhagen, Denmark, vii + 235 pp.

Obbard ME, Middel KR, Stapleton S, Thibault I, Brodeur V, Jutras C (2013) Estimating abundance of the Southern Hudson Bay polar bear subpopulation using aerial surveys, 2011 and 2012. Ontario Ministry of Natural Resources, Science and Research Branch, Wildlife Research Series 2013-01. 33pp

Paetkau D, Amstrup SC, Born EW et al. (1999) Genetic structure of the world’s polar bear populations. Molecular Ecology, 8, 1571-1584.

Parks Canada (2004) Paulatuuq oral history project: Inuvialuit elders share their stories. Parks Canada, Western Arctic Field Unit, Inuvik, NT.

Parkinson CL, Cavalieri DJ, Gloersen P, Zwally HJ, Comiso JC (1999) Arctic sea ice extents, areas and trends, 1978-1996. Journal of Geophysical Research, 104, 20837-20856.

Parkinson CL, Cavalieri DJ (2012) Antarctic sea ice variability and trends, 1979–2010. The Cryosphere Discussions, 6, 931-956.

Peacock E, Taylor MK (2007) Polar bears of western Hudson Bay: survey extension investigation. NWMB Project # 2-07-19, Igloolik, NU, 16 pp.

Peacock E, Laake J, Laidre KL, Born EW, Atkinson SN (2012) The utility of harvest recoveries of marked individuals to assess polar bear (Ursus maritimus) survival. Arctic, 65, 391-400.

Peacock E, Taylor MK, Laake J, Stirling I (2013) Population ecology of polar bears in Davis Strait, Canada and Greenland. Journal of Wildlife Management, 77, 463–476.

Polar Bear Technical Committee (PBTC) (2006) Minutes of the 2006 Polar Bear Technical Committee Meeting, St. John’s, Newfoundland and Labrador, February 2006. Canadian Wildlife Service, Edmonton, AB, 25 pp.

Polar Bear Technical Committee (PBTC) (2007) Minutes of the 2007 Polar Bear Technical Committee Meeting, Edmonton, Alberta, February 2007. Canadian Wildlife Service, Edmonton, AB, 31 pp.

Polar Bear Technical Committee (PBTC) (2008) Minutes of the 2008 Polar Bear Technical Committee Meeting, Inuvik, Northwest Territories, February 2008. Canadian Wildlife Service, Inuvik, NWT, 72 pp.

Polar Bear Technical Committee (PBTC) (2010) Minutes of the 2010 Polar Bear Technical Committee Meeting, Ottawa, Ontario, February 2010. Canadian Wildlife Service, Ottawa, ON, 29 pp. + status table appendix.

Polar Bear Technical Committee (PBTC) (2013) Meeting Minutes of the 2013 Polar Bear Technical Committee Meeting, Iqaluit, Nunavut, 5-7 February 2013.

Prestrud P, Stirling I (1994) The International Polar Bear Agreement and the current status of polar bear conservation. Aquatic Mammalogy, 20, 113–124.

Regehr EV, Amstrup SC, Stirling I (2006) Polar bear population status in the southern Beaufort Sea: U.S. Geological Survey Open-File Report 2006-1337, 30 pp.

Regehr EV, Lunn NJ, Amstrup SC, Stirling I (2007a) Effects of earlier sea ice breakup on survival and population size of polar bears in Western Hudson Bay. Journal of Wildlife Management, 71, 2673–2683.

Regehr EV, Lunn NJ, Amstrup SC, Stirling I (2007b) Supplemental materials for the analysis of capture-recapture data for polar bears in Western Hudson Bay, Canada, 1984–2004. U.S. Geological Survey Data Series, 304, 13 pp.

Regehr EV, Hunter CM, Caswell H, Amstrup SC, Stirling I (2010) Survival and breeding of polar bears in the southern Beaufort Sea in relation to sea ice. Journal of Animal Ecology, 79, 117–127.

Remote Sensing Systems (REMSS). Data REMSS. http://www.remss.com/data/msu/monthly_time_series/RSS_Monthly_MSU_AMSU_Channel_TLT_Anomalies_Land_and_Ocean_v03_3.txt (accessed 20 January 2014).

Riewe R (1976) Inuit land use in the High Canadian Arctic. In: Inuit land use and occupancy project, Vol. 1. (ed. Freeman M) pp. 173-184, Department of Indian and Northern Affairs, Ottawa, ON.

Rode KD, Amstrup SC, Regehr EV (2007) Polar bears in the southern Beaufort Sea III: Stature, Mass and Cub Recruitment in Relationship to time and sea ice extent between 1982 and 2006. United States Geological Survey, 32 p.

Rode KD, Peacock E, Taylor MK, Stirling I, Born E, Laidre K, Wiig Ø (2012) A tale of two polar bear populations: ice habitat, harvest, and body condition. Population Ecology, 54, 3-18.

Rosing-Asvid A, Born EW (1990) Fangst af isbjørn (*Ursus maritimus*) I Avanersuaq og Upernavik kommuner:en interviewundersøgelse. With English summary. Teknisk rapport – Grønlands Hjemmestyre, Miljø- og Naturforvaltning. Rapport nr. 23, December 1990, 64 pp.

Rosing-Asvid A (2002) The polar bear hunt in Greenland. Technical report No. 45. Greenland Institute of Natural Resources, Nuuk, 25 pp.

Schweinsburg RE, Lee LJ, Latour PB (1982) Distribution, movement, and abundance of polar bears in Lancaster Sound, Northwest Territories. Arctic, 35, 159–169.

Screen JA, Simmonds, I (2010) The central role of diminishing sea ice in recent Arctic temperature amplification. Nature, 464, 1334-1337.

Sou T, Flato G (2009) Sea ice in the Canadian Arctic Archipelago: Modeling the past (1950-2004) and the future (2041-60). Journal of Climate, 22, 2181-2198.

Stapleton S, Atkinson S, Hedman D, Garshelis D (2014) Revisiting Western Hudson Bay: Using aerial surveys to update polar bear abundance in a sentinel population. Biological Conservation, 170: 38-47.

Stapleton S, Peacock E, Garshelis D (2016) Aerial surveys suggest long‐term stability in the seasonally ice‐free Foxe Basin (Nunavut) polar bear population. Marine Mammal Science, 32(1): 181-201.

Stirling I, Andriashek D, Latour P, Calvert W (1975) The distribution and abundance of polar bears in the eastern Beaufort Sea. Final Report to the Beaufort Sea Project. Fisheries and Marine Service, Department of Environment. Victoria, BC, 59 pp.

Stirling I, Jonkel C, Smith P, Robertson R, and Cross D (1977) The ecology of the polar bear (Ursus maritimus) along the western coast of Hudson Bay. Occasional Paper. No. 33, Canadian Wildlife Service, Ottawa, ON.

Stirling I, Kiliaan HPL (1980) Population ecology studies of the polar bear in Northern Labrador. CWS Occasional Paper No. 42, Canadian Wildlife Service, 21 pp.

Stirling I, Calvert W, Andriashek D (1980) Population ecology studies of the polar bear in the area of southeastern Baffin Island. Occasional Paper No. 44. Canadian Wildlife Service, 30 p.

Stirling I, Andriashek D, Spencer C, Derocher AE (1988) Assessment of the polar bear population in the eastern Beaufort Sea. Final Report to the Northern Oil and Gas Assessment Program. Canadian Wildlife Service, Edmonton, AB, 81 p.

Stirling I, Derocher AE (1993) Possible impacts of climatic warming on polar bears. Arctic, 46, 240-245.

Stirling I, Lunn NJ (1997) Environmental fluctuations in Arctic marine ecosystems as reflected by variability in reproduction of polar bears and ringed seals. In: Ecology of arctic environments (eds. Woodin SJ, Marquiss M) pp. 167-181, Blackwell, Oxford, England.

Stirling I (1998) Polar Bears. Michigan: The University of Michigan, 220 p.

Stirling I, Lunn NJ, Iacozza J (1999) Long-term trends in the population ecology of polar bears in Western Hudson Bay in relation to climatic change. Arctic, 52, 294-306.

Stirling I, Lunn NJ, Iacozza J, Elliott C, Obbard ME (2004) Polar bear distribution and abundance on the Southwestern Hudson Bay Coast during open water season, in relation to population trends and annual ice patterns. Arctic, 57, 15-26.

Stirling I, Parkinson CL (2006) Possible effects of climate warming on selected populations of polar bears (*Ursus maritimus*) in the Canadian Arctic. Arctic, 59, 261-275.

Stirling I (2011) Polar Bears: The natural history of a threatened species. Markham, Ontario: Fitzhenry and Whiteside, 300 p.

Stirling I, McDonald TL, Richardson ES, Regehr EV, Amstrup SC (2011) Polar bear population status in the northern Beaufort Sea, Canada, 1971–2006. Ecological Applications, 21, 859–876.

Stirling I, Derocher AE (2012) Effects of climate warming on polar bears: a review of the evidence. Global Change Biology, 18, 2694–2706.

Stroeve J, Holland MM, Meier W, Scambos T, Serreze M (2007) Arctic sea ice decline: Faster than forecast. Geophysical Research Letters, 34, 1-5.

Stroeve JC, Serreze MC, Holland MM, Kay JE, Malanik J, Barrett AP (2012) The Arctic’s rapidly shrinking sea ice cover: a research synthesis. Climatic Change, 110, 1005-1027.

Swanson, KL, Tsonis AA (2009) Has the climate recently shifted? Geophysical Research Letters, 36, L06711.

Taylor MK, Bunnell F, DeMaster D, Schweinsburg R (1987c) Modelling the sustainable harvest of female polar bears. Journal of Wildlife Management, 51, 811-820.

Taylor MK, Lee LJ (1994) Tetracycline as a biomarker for polar bears. Wildlife Society Bulletin, 22, 83-89.

Taylor MK, Lee LJ (1995) Distribution and abundance of Canadian polar bear populations: a management perspective. Arctic, 48, 147–154.

Taylor MK, Akeeagok S, Andriashek D et al. (2001a) Delineating Canadian and Greenland polar bear (Ursus maritimus) populations by cluster analysis of movements. Canadian Journal of Zoology, 79, 690–709.

Taylor MK, Laake J, Cluff HD, Ramsay M, Messier F (2002) Managing the risk from hunting for the Viscount Melville Sound polar bear population. Ursus, 13, 185-202.

Taylor MK, Laake J, McLoughlin PD et al. (2005) Demography and viability of a hunted population of polar bears. Arctic, 58, 203-214.

Taylor MK, Laake J, McLoughlin PD, Cluff HD, Messier F (2006a) Demographic parameters and harvest-explicit population viability analysis for polar bears in M'Clintock Channel, Nunavut, Canada. Journal of Wildlife Management, 70, 1667-1673.

Taylor MK, Lee J, Laake J, McLoughlin PD (2006b) Estimating population size of polar bears in Foxe Basin, Nunavut using tetracycline biomarkers. File report to the Department of the Environment, Government of Nunavut, 13 pp.

Taylor MK, Dowsley M (2008) Demographic and ecological perspectives on the status of polar bears. Science and Public Policy Institute Special Report, 50 pp.

Taylor MK, Laake J, McLoughlin PD, Cluff HD, Born EW, Rosing-Asvid A, Messier F (2008a) Population parameters and harvest risks for polar bears (Ursus maritimus) of Kane Basin, Canada and Greenland. Polar Biology, 31, 491-499.

Taylor MK, Laake J, McLoughlin PD, Cluff HD, Messier F (2008b) Mark-recapture and stochastic population models for polar bears of the high arctic. Arctic, 61, 143-152.

Taylor MK, Laake J, McLoughlin PD, Cluff HD, Messier F (2008c) Demography and population viability of polar bears in the Gulf of Boothia, Nunavut. Marine Mammal Science, 25, 778-796.

Taylor MK, McLoughlin PD, Messier F (2008d) Sex-selective harvesting of polar bears. Wildlife Biology, 14, 52-60.

Taylor MK, Laake J, McLoughlin PD, Cluff HD, Messier F (2009) Demography and population viability of polar bears in the Gulf of Boothia, Nunavut. Marine Mammal Science, 25, 778-796.

Thiemann GW, Iverson SJ, Stirling I (2008) Polar bear diets and arctic marine food webs: insights from fatty acid analysis. Ecological Monographs, 78, 591-613.

Treseder L, Carpenter A (1989) Polar bear management in the southern Beaufort Sea. Information North, 15, 2-4.

Usher P (1976) Inuit land use in the west-central Canadian arctic. In: Inuit land use and occupancy project, Vol. 1. (ed. Freeman M) pp. 21-31, Department of Indian and Northern Affairs, Ottawa, ON.

van Vuuren DP, Stehfest E, den Elzen MG et al. (2011) RCP2.6: Exploring the possibility to keep global mean temperature increase below 2°C. Climatic Change, 109, 95-116.

Welch HE, Bergmann MA, Siferd TD et al. (1992) Energy flow through the marine ecosystem of the Lancaster Sound Region, Arctic Canada. Arctic, 45, 343-357.

York JC (2012) Status of Canadian polar bear subpopulations: A 2012 Status Report.

HBA Thesis, Lakehead University, Department of Geography, Thunder Bay, Ontario**.** 103 pp.

FIGURE LEGEND

**S1 FIG. S1.** Baffin Bay (BB) subpopulation trajectories from 1997-2013. A comparison of the effect of different BB survival rates (Taylor et al., 2005 [Natural]; Peacock et al., 2012 [Natural and Total]) for producing a subpopulation trajectory for the 2003-2013 period.

**S5 FIG. S1.** The potential effect of truncated runs on subpopulation abundance was estimated from a series of RISKMAN simulations using increasing initial subpopulation variance (CV) for the Viscount Melville Sound (VM) subpopulation. The simulations were run for 5000 iterations over a 20 year period under a harvest moratorium.

**S5 FIG. S2.** The potential effect of truncated runs on geometric subpopulation growth rate estimated from a set of 100 Monte Carlo iterations for the Viscount Melville Sound (VM) subpopulation for 20 year period under a harvest moratorium.

**S7 FIG. S1.** HadCRUT4 annual global temperature for the 1980-2013 period. A breakpoint regression was used to divide the period into two distinct intervals (Table G3).

**S7 FIG. S2.** REMSS annual Arctic temperature for the 1980-2013 period. A breakpoint regression was used to divide the period into two distinct intervals (Table G3). The difference between the two interval means was -0.616 (0.104) (Table G9).

**S7 FIG. S3.** NOAA-NODC global ocean heat content (0-700 meters) for the 1980-2013 period. A breakpoint regression was used to divide the period into two distinct intervals (Table G3).

**S7 FIG. S4.** NSIDC annual Arctic sea ice extent for the 1980-2013 period. A breakpoint regression was used to divide the period into two distinct intervals (Table G3).

**S7 FIG. S5.** Observed monthly sea ice extent (NSIDC) for the Arctic from January 1980 to December 2013.

**S7 FIG. S6.** Annual global temperature (HadCRUT4) and monthly sea ice extent (NSIDC) for the Arctic during the January 1980 to December 2013 period.

**S7 FIG. S7.** Global mean temperature near−term projections relative to 1986−2005 (From: Kirtman et al., (2013) Figure 11.25(a)).

**S7 FIG. S8.** Arctic September sea ice extent (x10^6^ km^2^) from observations (thick red line) and 13 IPCC AR4 climate models, together with the multi-model ensemble mean (solid black line) and standard deviation (dotted black line). Models with more than one ensemble member are indicated with an asterisk. Inset shows 9-year running means (From Stroeve et al., 2007, Fig. 1).

**S7 FIG. S9.** Arctic March sea ice extent (x10^6^ km^2^) from observations (thick red line) and 18 IPCC AR4 climate models together with the multi-model ensemble mean (solid black line) and standard deviation (dotted black line). Models with more than one ensemble member are indicated with an asterisk. Inset shows 9-year running means (From Stroeve et al., 2007, Fig. 2).
